# A Retinoblastoma Orthologue Is a Major Regulator of S-Phase, Mitotic, and Developmental Gene Expression in *Dictyostelium*

**DOI:** 10.1371/journal.pone.0039914

**Published:** 2012-06-29

**Authors:** Kimchi Strasser, Gareth Bloomfield, Asa MacWilliams, Adriano Ceccarelli, Harry MacWilliams, Adrian Tsang

**Affiliations:** 1 Biology Department and Centre for Structural and Functional Genomics, Concordia University, Montreal, Canada; 2 MRC Laboratory of Molecular Biology, Cambridge, United Kingdom; 3 Wellcome Trust Sanger Institute, Hinxton, United Kingdom; 4 Siemens Corporate Technology, Munich, Germany; 5 Neuroscience Institute Cavalieri Ottolenghi, University of Torino, Torino, Italy; 6 Biozentrum der Ludwig-Maximilans-Universität, Munich, Germany; University of Cambridge, United Kingdom

## Abstract

**Background:**

The retinoblastoma tumour suppressor, Rb, has two major functions. First, it represses genes whose products are required for S-phase entry and progression thus stabilizing cells in G1. Second, Rb interacts with factors that induce cell-cycle exit and terminal differentiation. *Dictyostelium* lacks a G1 phase in its cell cycle but it has a retinoblastoma orthologue, *rblA.*

**Methodology/Principal Findings:**

Using microarray analysis and mRNA-Seq transcriptional profiling, we show that RblA strongly represses genes whose products are involved in S phase and mitosis. Both S-phase and mitotic genes are upregulated at a single point in late G2 and again in mid-development, near the time when cell cycling is reactivated. RblA also activates a set of genes unique to slime moulds that function in terminal differentiation.

**Conclusions:**

Like its mammalian counterpart *Dictyostelium*, RblA plays a dual role, regulating cell-cycle progression and transcriptional events leading to terminal differentiation. In the absence of a G1 phase, however, RblA functions in late G2 controlling the expression of both S-phase and mitotic genes.

## Introduction

*Dictyostelium* is not a traditional model system for cell-cycle studies. Most of the work done on the cell cycle of this organism was motivated by other questions, particularly the desire to understand *Dictyostelium* development. In the latter process, amoebae differentiate into either stalk cells or spores. Pathway choice is determined by the cell-cycle position when development begins [1]. Regulation of the eukaryotic cell cycle is fairly well understood, but this has brought few insights about developmental-pathway choice in social amoebae largely because the *Dictyostelium* cell cycle is atypical. In *Dictyostelium* the G1 phase is very short or non-existent [2]. The G1/S transition is the major point at which the cell cycle is controlled in most eukaryotes. Since *Dictyostelium* appears to bypass this point, it has been unclear how the *Dictyostelium* cell cycle is organized.

Despite this unusual configuration *Dictyostelium* has a relatively normal-appearing set of genes regulating the cell cycle. There is a single cyclin-dependent protein kinase (*cdk1*) of the cell-cycle clade, three cell-cycle cyclins, a clearly recognizable anaphase promoting complex, a polo kinase, an aurora kinase [3], several *nimA*-related molecules, three *wee* kinases, and one *cdc25* [4]. *Dictyostelium* even possesses genes that are associated with the G1/S transition in higher cells. In particular, there is an orthologue of the retinoblastoma susceptibility protein, Rb, as well as the activator E2F (gene ID **DDB_G0284129**), the transcription factor with which Rb characteristically interacts, and the E2F-dimerization partner DP (gene ID **DDB_G0276799**) [4].

Rb has two well-known roles in animal and plant cells, one in regulating transcription at the G1/S transition, the other in facilitating terminal differentiation [5]. The *Dictyostelium* Rb orthologue, *rblA,* is upregulated 200-fold in differentiating spores. Vegetative *rblA*-deficient cells are smaller than wild-type cells and they enter development prematurely. Although *rblA*-nulls develop normal fruiting bodies, they show a preference for the stalk pathway when mixed with wild-type cells [4]. These results suggest that RblA plays a role during growth and late development of *Dictyostelium*.

In the present study, we used comparative transcriptomics to further investigate the role of RblA in *Dictyostelium*. Using DNA microarrays [6], [7] to analyse the transcriptomes of wild-type and *rblA* disruptant cells, we found that the most significant differences occurred in S-phase specific genes. Using massively parallel mRNA sequencing (mRNA-Seq), we identified a larger group of transcripts, representing about 4% of *Dictyostelium* genes, that are overexpressed 2- to 80-fold in the absence of RblA function. The collection includes virtually all *Dictyostelium* genes with known or suspected roles in S phase or mitosis. A further experiment using synchronized wild-type cells showed that most RblA-repressed genes are maximally expressed at a single point in G2, one to two hours before mitosis. We also used an existing mRNA-Seq data set [8] to characterize the expression of RblA-regulated genes in the *Dictyostelium* multicellular stage. Virtually all RblA-repressed cell-cycle genes are upregulated strongly in mid-development; this is consistent with classical and recent reports of cell-cycle activity in *Dictyostelium*’s migrating slugs [2], [9], [10], [11], [12], [13]. During terminal differentiation, most cell-cycle genes are turned off again while a different class of RblA targets, the RblA-activated genes, are upregulated. These results suggest that RblA is important for both the cell cycle and differentiation of *Dictyostelium*. Unlike the role of Rb in the G1/S phase transition of the animal cell cycle, however, *Dictyostelium* RblA exerts its effect in late G2 and is marked by its transcriptional regulation of both mitotic and S-phase specific genes.

## Results

### Congruence of Microarray and mRNA-Seq Data

We prepared mRNA samples from growing and developing cells of a *rblA*-disruptant [4] and the parental strain, then analysed the samples using both microarrays [7] and mRNA-Seq. For all the conditions and strains, a total of three biological and two technical replicates were performed using DNA microarrays and two biological replicates were analysed by RNA-Seq. The number of genes showing significant differences in expression was higher in the mRNA-Seq data set than in the microarrays (367 versus 251 transcripts with *p-*values of <0.05). Many of the transcripts whose differential expression was judged significant in the microarrays were also strongly regulated in the mRNA-Seq data set (**Figure S1)**. Expression changes were similar for the most abundant transcripts. Low abundance transcripts behaved heterogeneously. Expression changes were higher on the average in the mRNA-Seq data set but we also saw some genes, judged significant in the microarrays, which showed little or no regulation in the mRNA-Seq data. These genes included several whose transcripts were not detectable by mRNA-Seq. Such events are presumably microarray false-positives resulting from cross-hybridization and deficiencies in array construction. We also performed qRT-PCR to support this cross-platform validation on selected genes (**Table S1**).

### Overall Structure of the mRNA-Seq Data

**Figure 1:**
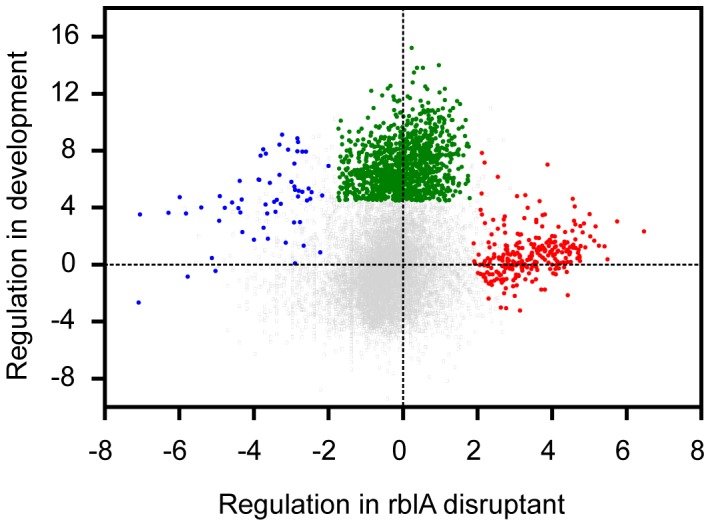
Groups of RblA-regulated genes . Scatter plot of RblA-specific regulation during development (x-axis; scale is log2) compared to developmental regulation (y-axis; scale is log_2_).Each point represents a gene. The red dots are genes repressed by RblA in both growth and development. The green dots are genes induced during development but not significantly regulated by RblA. The blue dots are genes whose activation depends wholly or partly on RblA.

To visualize the mRNA-Seq data set as a whole, we plotted, gene by gene, the regulation factor in the *rblA* disruptant versus the regulation in late development **(** **Figure 1** **).** Most of the points fell near the origin in this plot but three separate clusters of genes were found to depart from this pattern. The largest group consists of developmentally upregulated genes not controlled by RblA (green dots). These genes have been extensively studied in *Dictyostelium* and are not discussed further here. A second group of transcripts (red dots) is upregulated in the disruptant, more so in developing than in growing cells. This group is referred to as the RblA-repressed genes. A third grouping (blue dots) contains genes that are upregulated in development but downregulated in the *rblA* mutant. We call these RblA-activated genes. The genes repressed or activated by RblA are described below.

### RblA-repressed Genes

**Table 1** gives an overview of the genes that are overexpressed in the *rblA* disruptant and thus presumably normally repressed, directly or indirectly, by RblA. All of the RblA-regulated genes with regulation factors of 2 or greater in growing and/or developing cells are listed in **Table S2**. Macros are included to assist the user in graphing expression profiles and gene IDs are linked to DictyBase [14].

**Table 1:** Major groups of RblA-repressed genes.

**Cell cycle controllers**	
Protein-level regulators	*cdk1*, *cks1*, *nek2*, Aurora (*aurK*), *wee* kinase, 5 anaphase-promoting complex subunits, *pich*
Transcriptional modulators	*rblA*, DP (*tfdp2*), *lin9*, *lin54*, *myb0*, SMYD3, *ada2*, L-antigen/PCC1, Ki-67, C/EBP
**S-phase actors or regulators**	
Origin recognition complex	*orcD*
Other initiation factors	*cdc6*, *cdc45*, *gins1-4*, *ctf4*
DNA helicase subunits and interactors	*mcm2*-*7*, Timeless, *wrnip1*
DNA polymerase subunits	all recognizable subunits of pol alpha, delta, and epsilon
Nucleotide biosynthesis genes	*rnrA*&*B*, dCMP deaminase,
Sliding clamp and clamp loaders	*pcna*, *rfc1-5*, *ctf8*, *dcc1*, ELG1
Other replication fork factors	flap endonuclease (*repG*), DNA ligase I (*lig1*), DNA topoisomerase II (*top2*)
Cohesins and interactors	*smc1*,*3,5*&*6*, STAG, Nipped-B, *rad21*, *pds5*, *eco1*
Histone mRNA processing factors	*eriA*, COBRA1
Chromatin disassembly/assembly factors	*ssrp1*, *asf1*, HAT1, HAT2/RbAp48 (*rbbD*), CAF1a&b, 2 SAPs
DNA mismatch repair	*msh2*&*3*&*6*, *myh*, *apnA*
Post replication repair	*rad18*
Double strand break repair (HR)	RepA (*rfa1*), *rad51*, *rad52*
Double strand break repair (NHEJ)	*xrcc4*, *lig4*, putative aprataxin, 53BP1
Intrastrand crosslink repair	*mtmr15*
9-1-1 complex and interactors	*rad9*, *hus1*, Rad53/CHK2 (*fhkA*)
**Mitotic actors or regulators**	
Centrosomal components and interactors	*spc97&98*, CP75, CP91, *mps1*
Centromere and kinetochore	*spc25*, *ndc80*, *nuf2*, Cenp68, Haspin, SPC105
Condensins and interactors	*smc2*&*4*, *ncapD2*, *D3*, *Ga, Gb*, *G2*, *H*&*H2*
Spindle components	*tubC*, *tubC* folding cofactor (*tbcD*) *eb1*, *clasp*, 7 mitotic kinesins (*kif*s)
Spindle checkpoint components	*bub1&3*, Mad2, *cdc20*, *ube2c*, Separase (*espl1*)
Cytokinesis regulators	INCENP (*icpA*), 2 dynamin-related proteins (*dlp*)
**Transposable elements**	
Skipper	9 Skipper POL and GAG genes, plus 2 diverged GAG
Tdd-5	one of two *Dictyostelium* Tdd-5 sequences; this one contains a consensus E2F element
**Conserved proteins associated with proliferation or cancer**	
Shaker-related voltage gated potassium channel correlated with proliferation in vertebrate Schwann cells [20]	
Ube2s, ubiquitylates VHL and thus indirectly upregulates HIFalpha and tumour angiogenesis in vertebrates [23]	
**Other widely conserved proteins**	
Putative orthologue of Monad (*wdr92*) which potentiates apoptosis in response to TNF-alpha [22]	
Putative orthologue of SIVA, an E2F-induced proapoptotic protein in vertebrates [21]	
Vacuolar import and degradation protein Vid27	
CARKD, universally conserved protein of unknown function	

These genes are at least twofold upregulated in the *rblA* disruptant. Details and links to the individual genes are in **Table S2**. In the interest of clarity where the gene name is unclear or cumbersome the name of the corresponding protein is given.

A group of genes found to be upregulated in the *rblA* disruptant code for proteins involved in the overall control of cell-cycle functions. These include *cdk1*, *cks1*, and aurora kinase. At least three functional groups of S-phase genes are overexpressed in the *rblA* disruptant. Genes whose products are directly associated with replication initiation or replication fork progression - including the ATP-binding Cdc6, members of the origin of replication (*orc*) complex, and all six subunits of the mini-chromosome maintenance (mcm) helicase - are upregulated in the mutant. Genes associated with the biosynthesis of deoxyribonucleotides such as the large and small subunits of ribonucleotide reductase and those involved in the disassembly/reassembly of chromatin are also upregulated in *rblA-*deficient cells.

Genes associated with DNA repair and rearrangement repressed by RblA include components of the DNA mismatch repair system. Genes whose products are involved in rescuing stalled replication forks by homologous recombination are also upregulated in the *rblA* disruptant, including the PCNA-targeting E3 ubiquitin ligase RAD18 and the recombination enzymes RAD51 and RAD52. There is however dichotomy in the role of RblA on the non-homologous end joining (NHEJ) pathway. The genes encoding members of the “X4L4” complex are RblA-repressed while several others (*dnapkcs*, *ku70*, *ku80*, *polB*, and *wrn*) are not regulated by RblA.

Mitotic genes are also repressed by RblA. For example, most *Dictyostelium* condensins are upregulated in the *rblA* disruptant. The annotation of this group of genes, consisting of complete sets of both type I and type II condensins, was based initially on the results of this study. Other RblA-repressed M-phase genes include those whose products are important in spindle assembly or spindle mechanics. Notably, the seven overexpressed kinesins correspond exactly to those thought to be involved in mitotic processes [15], [16]; none of the kinesins with putative functions in vesicle transport are regulated by RblA. RblA-repressed genes with probable roles in cytokinesis include the inner centromere protein INCENP and two genes coding for dynamin-like proteins [17]. In addition, five of the ten identifiable subunits of the anaphase promoting complex are repressed by RblA.

Our data show RblA regulation of several highly conserved genes whose functions in the cell cycle are poorly understood. These include *shaker*, and *ube2s* whose expression correlates with normal or abnormal proliferation and two (*wdr92* and SIVA) that are considered to be apoptotic genes in metazoans [18], [19], [20], [21], [22], [23]. *Dictyostelium* may be useful in clarifying the functions of these genes.

Among the genes most strongly upregulated in the *rblA* disruptant are open reading frames (ORFs) of five transposable elements. A collective of 19 Tdd-4 elements is upregulated 25-fold. The *Dictyostelium* database lists two occurrences of the Tdd-5 transposon. One of these (DDB_G0290955) is upregulated in *rblA*-deficient cells 85-fold during growth (p-value <0.01) and 40-fold during development (p-value <0.01). The retrotransposable element Skipper and two divergent Skipper GAG fragments are also upregulated in the *rblA* disruptant.

### Expression of *rblA*-repressed Genes during the Cell Cycle

**Figure 2:**
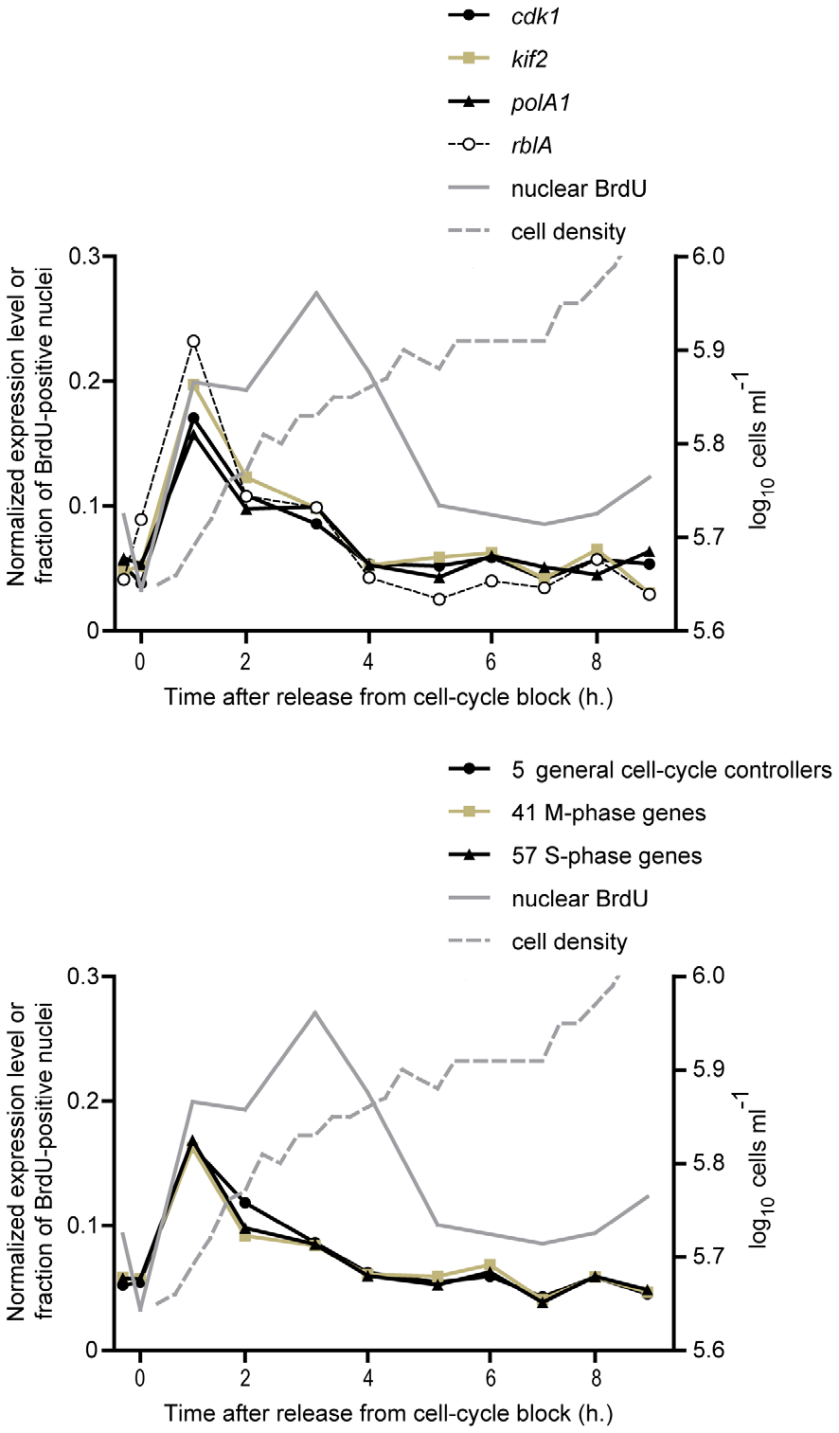
Cell-cycle regulation after cold synchronization. Cells were synchronized using cold arrest and released from the block at 0 h. RNA samples were collected at hourly intervals over a 12 hour period for mRNA sequencing. Cells were counted every 30 minutes (grey dashed line) and pulse-labelled every hour with bromodeoxyuridine to monitor passage through S-phase (grey line). Panel (A) shows the expression pattern of selected genes: *cdk1*, *kif2*, *polA*, and *rblA*. In panel (B), the expression profiles of genes which act at specific phases of the cell cycle are grouped. Genes with RblA-repression factors of 2 or greater whose products are known to play roles in the cell cycle, including 5 general cell cycle controllers, 41 M-phase genes, and 57 S-phase genes were averaged and graphed. Note that because of the incomplete synchronization, modulation of greater than threefold is not expected even if a gene is expressed exclusively at the G2/M transition. The time in hours (x-axis) and normalized regulation level (y-axis) are shown.

Are *rblA*-repressed genes with putative cell-cycle functions regulated during the *Dictyostelium* cell cycle? To answer this question, we synchronized wild-type cells using the temperature block-and-release method [24]. In parallel, we cultured an aliquot of the synchronized cells in the presence of bromodeoxyuridine to monitor progression through S phase (**Figure 2**). The number of bromodeoxyuridine-positive cells peaked 3 hours after release from the cell-cycle block. The duration of the *Dictyostelium* cell cycle is approximately 8 hours with a 10-minute M phase followed immediately by a 30-minute S phase [2]. The levels of most RblA-repressed genes fluctuate during the cell cycle, reaching a maximum one hour after the cells have resumed growth. Since there is no G1 phase [2], [9] this places their accumulation during the cell cycle in late G2 shortly before mitosis. These results agree with those obtained in RNA-blot analysis and reporter-gene studies for *rnrB* [1].

In this experiment, the *rblA* transcript itself behaved very much like the genes that it represses, increasing shortly before S phase. It is therefore probable that the RblA protein is inactivated at this point although this remains to be shown directly. In animal and plant cells, cyclin D initiates Rb inactivation at the G1/S transition [25], [26]. Our cold-synchronization data suggest that it may play a parallel role in *Dictyostelium*; *cycD* mRNA levels increased 7.5-fold during the cold shock (*p*<0.01) and preceded by one hour the maximum expression of RblA-repressed genes.

Further examination of the cell-cycle data revealed a number of genes that show strong cell-cycle regulation but change little in the *rblA* disruptant. Some of these such as *cdc25* and *cycB* are putative cell-cycle regulators. Others, like coronin B or the folliculin-interacting protein orthologue DDB_G0289243, code for proteins not previously recognized as being important in the cell cycle. It thus seems likely that a RblA*-*independent pathway for cell-cycle regulation exists.

### Expression of RblA-regulated Genes in Development

**Figure 3:**
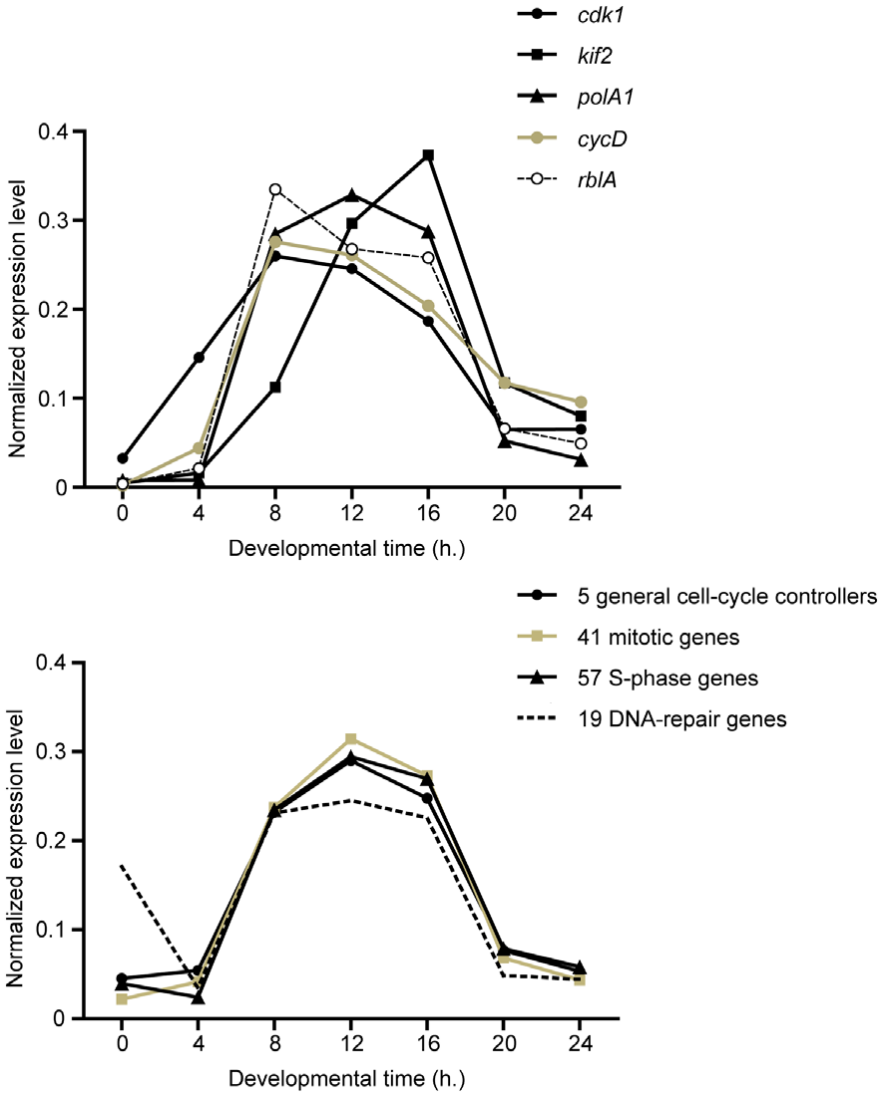
Developmental regulation of cell-cycle genes. (A) Developmental regulation of the selected cell-cycle genes *cdk1*, *kif2*, *polA1*, *cycD*, and *rblA*. (B) Averaged transcriptional profiles during development of groups of cell-cycle genes including 5 general cell cycle controllers, 41 mitotic genes, 57 S-phase genes, and 22 DNA repair genes. These profiles were generated by reanalysing the raw data from Parikh *et al* [8]. The developmental time in hours (x-axis) and normalized mRNA levels (y-axis) are shown.

Cell-cycle activity during *Dictyostelium* multicellular development has been reported [2], [9], [10], [11], [12], [13]. We examined the developmental expression of *Dictyostelium* cell-cycle genes using previously published mRNA-Seq data [8]. In this study, developing cells were harvested for mRNA-Seq analysis at 4 hour-intervals over a 24 hour period until terminal differentiation. To ensure strict comparability between datasets the raw reads were obtained (courtesy of G. Shaulsky and A. Parikh) and reanalysed using the same method used to analyse the cell-cycle dataset. **Figure 3** shows the developmental profiles of *cdk1*, *kif2*, *polA1, cycD,* and *rblA* as well as the averaged profiles from 5 general cell-cycle control genes, 41 mitotic genes, 57 S-phase genes, and 19 DNA-repair genes. Most cell-cycle genes are maximally expressed in mid development at the tipped aggregate and slug stages. Like the cell-cycle experiment, the transcriptional profile of *rblA* is comparable to those of the genes that it represses with a peak between 8 to 16 hours of development [4]. Taken together this suggests that RblA is inactive at the protein level in most cells during this period. Interestingly, the *cycD* transcript peaks shortly but significantly (p<10^−44^, by chi-squared comparison of profiles) before the maximal expression of RblA-repressed genes, again suggesting a role for cyclin D in RblA inactivation.

In metazoans, Rb interacts with other activators of terminal-differentiation genes to facilitate differentiation and stabilize the differentiated state [27], [28]. We identified a group of genes that was upregulated in development but downregulated in the *rblA* disruptant. These genes are listed in **TableS2**. Four genes activated by RblA during development code for transmembrane proteins containing an immunoglobulin E-set domain. Eight others are related to *hssA* (high copy suppressor of STATa). Two of these (sigN173 and sigN175) belong to a family of prestalk-specific genes transcriptionally regulated by the serum-response related factor SrfA [29]. In contrast to the RblA-repressed genes, most of the RblA-activated genes are novel with no recognizable homologues outside the cellular slime moulds. This is not surprising since they presumably code for proteins that are responsible for the genesis of slime mould specific structures or natural products.

**Figure 4:**
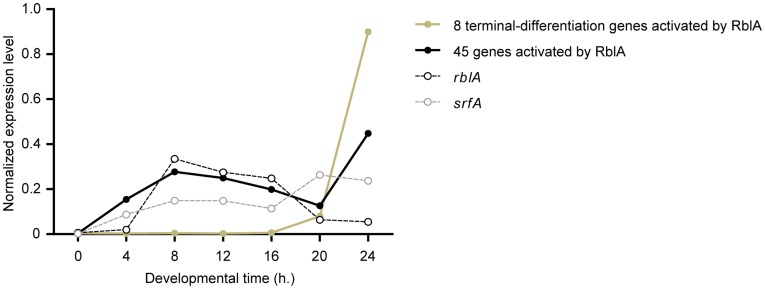
Developmental regulation of RblA-activated genes. Cells were developed for the amount of time indicated and analysed using mRNA-sequencing. The averaged-developmental profiles of 8 terminal-differentiation genes and 45 genes regulated during development by RblA, as well as the profiles for *rblA*, and *srfA*. Raw data from Parikh *et al* reanalysed [8]. The developmental time in hours (x-axis) and normalized mRNA levels (y-axis) are shown.

**Figure 4** shows the average developmental expression profiles the RblA-activated genes. In wild-type cells, eight genes are expressed during terminal differentiation and 45 genes are upregulated during mid-development. In most cases the vegetative mRNA levels are too low to give usable cell-cycle profiles. An exception is the putative GATA-family transcription factor coded by *comH*, which is 100-fold downregulated in the *rblA* disruptant during growth and over 30-fold downregulated in the *rblA* disruptant during development. The *comH* transcript has an expression peak in mid-development and shows strong cell-cycle regulation, but it is expressed in early G2 in a phase that overlaps only slightly with the expression of RblA-repressed genes (**Table S2**).

### A *Dictyostelium* Cell-cycle Transcriptional Network

To our knowledge, a comprehensive picture of cell-cycle transcriptional control exists only for yeast [30]. Unfortunately most transcriptional regulators of the yeast cell cycle have no orthologues in higher eukaryotic cells. Our data suggests that the parallel between metazoans and *Dictyostelium* may be closer. In amoebae, we find orthologues of several histone modifiers and other regulators suspected to control the expression of metazoan cell-cycle genes (**Table 1**). The genes encoding these transcriptional proteins are repressed by RblA, and they are expressed during development in a fashion typical of cell-cycle genes. Prominent among them is a relative of the conserved histone lysine methyltransferase known as the Set and MYnd Domain-containing protein, SMYD3. This protein accumulates in the nucleus of S-phase and G2/M cells, and is upregulated in the majority of colorectal and hepatic cancers [31].

*RbbD* (an orthologue of RpAp48), *lin9*, and *lin54* are putative members of the LINC complex implicated in the regulation of G2/M genes in mammals [32]. These three genes are upregulated in the *rblA* disruptant. DDB_G0280079 codes for a protein similar to ADA2, a subunit of the trimeric histone-acetyltransferase complex SAGA. Levels of the *ada2* transcript are 2.4-fold higher in the *rblA* disruptant in both growing and developing cells. Finally, DDB_G0274491, a gene similar to the budding yeast PCC1, is repressed by RblA and is cell-cycle and developmentally regulated. In yeast PCC1 recruits SAGA to specific promoters including those of genes involved in cell-cycle progression [33]. The human orthologue of PCC1 is the cancer/testis antigen (L-antigen), a gene overexpressed in a wide variety of cancers [34].

**Figure 5:**
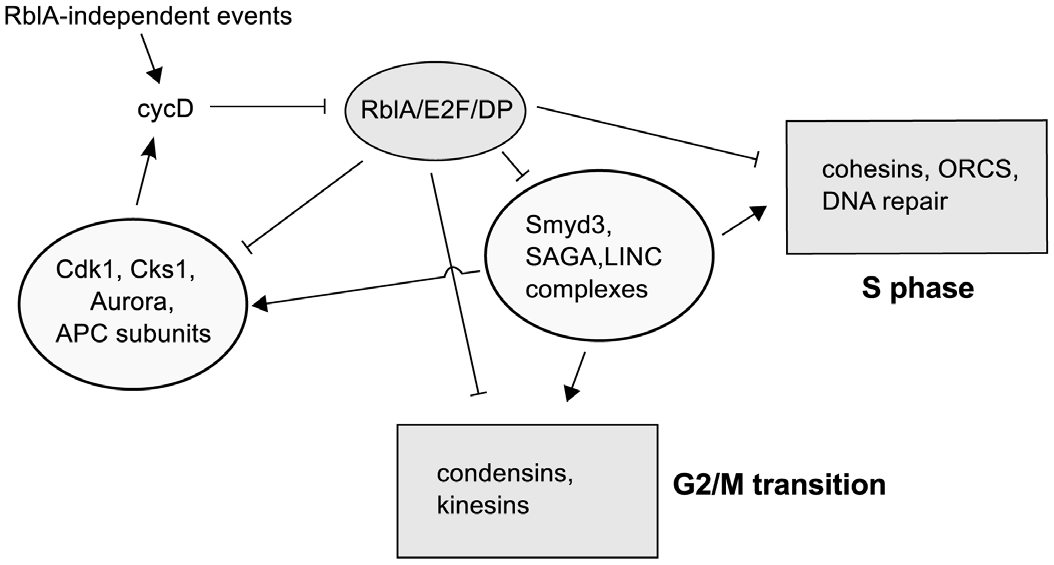
A schematic model of the *Dictyostelium* cell-cycle regulatory network. We propose a model based partly on our gene expression data in which the retinoblastoma homologue RblA (with E2F and DP) represses most of the important S phase and G2/M-phase genes at the transcriptional level. This repression acts directly by down-regulating activators such as *cdk1*, and indirectly by targeting factors that control the expression of genes required for cell-cycle progression. Among the factors repressed by RblA are the SAGA complex and the histone methyl transferase SMYD3, which are predicted to bind to the promoters of many cell-cycle genes. The LINC complex, also RblA-repressed, is implicated in the regulation of G2/M genes in mammals (see citations in the text). RblA-independent regulators, such as Cdc25, CycB, and CycD, acting with Cdk1 help to complete the network.

The control of cell-cycle genes in *Dictyostelium* must involve a variety of pathways. This is certainly true in higher eukaryotes but the overall structure of the system has not been deciphered. In amoebae, where the cell-cycle transcriptional network appears to have RblA at its apex, the situation may be more approachable (**Figure 5**). Using genetic manipulations and mRNA-Seq technology, it may be possible to clarify the downstream network interconnections.

## Discussion

Previous studies on animal and plant cells over- or under-expressing Rb have consistently identified four classes of genes as targets of the Rb/E2F axis. Not surprisingly, the list includes many genes whose products are involved in DNA replication. Genes coding for chromatin-modifying proteins form a second group and DNA-repair genes a third. Finally, Rb and/or E2F have been shown to regulate a number of mitotic genes.

Most of the RblA-repressed genes that we have found in *Dictyostelium* fall into these four categories. However, mRNA-Seq technology allowed us to see these groups more clearly than microarray-based studies. We have recovered essentially all genes with important roles in DNA replication as well as most of the genes known or suspected to be involved in the *Dictyostelium* M phase. We also capture more detail in the groups of chromatin-modifying and DNA-repair molecules. The DNA-repair genes are highly heterogeneous in their response to disruption of *rblA*, and it is primarily the repair pathways linked to DNA replication (“methyl-directed” repair, homologous recombination, and replication fork rescue) that are overexpressed in the absence of RblA function.

In addition to these classes of RblA targets, two DNA transposons and three retrotransposons showed particularly strong overexpression in the *rblA* disruptant. DNA transposons increase in number primarily by transposition in S phase [35] while retrotransposons create double-strand breaks that must be repaired by the cellular machinery [36]. Since transposable elements of both types rely on the DNA-replication machinery for dispersal in the genome, it makes sense that their expression should correlate with that of S-phase genes. Transposition is a major modality of mutagenesis and since Rb is inactivated, directly or indirectly, in most tumours [37] Rb-regulated transposon transcription may contribute to genomic instability in cancer.

RblA also appears to control cell-cycle genes during the *Dictyostelium* multicellular stage. Although initially interrupted, cell cycling resumes at the slug stage [2], [9], [10], [11], [12], [13]. We found that cell-cycle genes are almost universally activated during an 8-hour period preceding slug formation. The upregulation levels are comparable to the overexpression levels in the *rblA*-null vegetative cells, suggesting that RblA is largely inactivated in most cells during mid-development.

Cell-cycle genes are, for the most part, repressed by RblA. A completely different group of genes is activated by RblA during late development. The *rblA* transcript itself is downregulated during this period so transcriptional regulation cannot be responsible for the increased expression of these genes. This implies that *Dictyostelium* RblA is activated at the protein level during late development. The set of *rblA-*activated genes includes two that are transcriptionally regulated by SfrA, a serum-response factor related protein [38]. *srfA* is itself induced in late development but not to the same extent as its known targets. This observation suggests that the genes may be coregulated by SrfA and RblA, perhaps as the result of a direct protein-protein interaction, although a complex of this sort has not been described in vertebrate cells. Alternatively, a further component may be involved. Two *Dictyostelium* GATA family proteins, *comH* (also known as *gtaB*) and *gtaT* are activated by RblA. An interaction between Rb and a GATA-family transcription factor has been reported in mice [39].

The cell cycles of most eukaryotes include a prolonged G1 phase, but Amoebozoans appear to depart from this pattern. Classical studies suggest that a substantial G1 phase is lacking in both *Physarum* [40], [41], [42] and *Acanthaomeba* [43], [44]. *Dictyostelium* amoebae enter S phase directly after mitosis and often before the completion of cytokinesis. When cells are allowed to attach to a substrate and S-phase cells labelled by a short pulse of [^3^H]thymidine or bromodeoxyuridine, marked cells are frequently found in pairs (HKM, unpublished observations) [2]. This is expected if cells that divide after attachment immediately begin DNA synthesis. One also occasionally sees late-telophase cells, sometimes with lagging chromosomes, in which DNA synthesis appears to have already begun [11], [45]. Although one report, based largely on flow cytometry of whole cells, suggests that *Dictyostelium* prespore cells and spores are arrested in G1 [46], this was not confirmed by a study of the DNA contents of individual nuclei [4], [9].

The basis for the lack of G1 in Amoebozoans is obscure. Intriguingly, the replication-licensing factor *cdt1* found in both animals and plants is missing and apparently not necessary in *Dictyostelium*. *Dictyostelium* also lacks identifiable homologues of Cdk inhibitors (CKIs) which play an important role in the G1/S transition of both fungi and metazoans [47]. Another protein missing in *Dictyostelium* is p53. In vertebrates it plays an important role in cell-cycle arrest following DNA damage, which proceeds via p21 to Cdk inhibition ultimately leading to Rb repression of cell-cycle genes [48], [49]. Both p21 and p53 are lacking in *Dictyostelium* but a DNA-damage induced cell cycle checkpoint has been described [9].

In some aspects, regulation of the *Dictyostelium* cell cycle more closely resembles regulation in plants than in metazoans. In metazoan cells, Rb inactivation at the G1/S transition is driven by a positive feedback loop involving cyclin E. Cyclin E binds to Cdk2 and the complex, in turn, phosphorylates Rb. This event frees the transcription factor E2F to activate the *cycE* gene. Plants do not have cyclin E. Rather the switch involves transcriptional upregulation of E2F and its dimerization partner, DP, at the G1/S transition and *cdkB* at G2/M [50], [51]. In *Dictyostelium*, *cdk1* and DP (*tfdp2*) are strongly upregulated in the *rblA* disruptant and both are regulated as cell-cycle genes.

In metazoans, mitotic entry is accompanied by a prominent peak in the transcription of cyclin A. In plants, both A- and D-type cyclins peak at the G2/M transition and in some cases D-type cyclins appear to be rate-limiting [51]. In *Dictyostelium* both cyclins peak shortly before M phase; *cycD* was induced before most cell-cycle genes including *cycA* in our cold-synchronization experiment. In the developmental time course prepared by Parikh and colleagues [8], *cycA* and *cycD* show sharp peaks slightly before the maximum expression of RblA-repressed genes.

In animals, Rb is best known as a stabilizer of G1, blocking S-phase entry and supporting G1 cell-cycle exit to differentiation. There is, however, evidence for an involvement of Rb/E2F at the G2/M transition. Three studies of E2F-overexpressing mammalian cells [52], [53], [54] each identified half-a-dozen mitotic genes as E2F targets, as did a survey of genes whose promoters are precipitable with antibodies to E2F1 or E2F4 [55]. Markey *et al.* [56] found 20 mitotic genes whose activity decreased upon Rb overexpression; Jackson *et al*. [49] found overexpression of 14 mitotic genes in cells lacking the Rb-related proteins p107 and p130. In *Drosophila*, Dimova and colleagues [57] found 8 mitotic genes among Rb/E2F targets. More recently, Date *et al*. [58] showed that the mitotic regulator Borealin is a Rb target in mammalian cells. In green plants, most of the cell-cycle genes regulated by the Rb/E2F axis are associated with the G1/S transition [59] but the G2/M-specific *cdkB* is also a known E2F target [51].

Here we show that RblA represses virtually all S-phase genes in addition to most genes required for entry to, progression through, and exit from mitosis in *Dictyostelium*. We saw 57 S-phase genes and 41 mitotic genes that are strongly regulated by RblA. The degrees of repression are similar and both groups of genes are expressed at a single point in the cell cycle about two hours before entry to M.

The overall picture is one of a regulatory system similar to higher eukaryotic cells but missing a number of components known in metazoans, and reminiscent in some aspects of plants. The *Dictyostelium* cell cycle is thus configured as one might expect in a common ancestor of animals and plants. A recent phylogeny, incorporating new information from sponges and placozoans, places the Amoebozoan branch before the divergence of animals and plants [60]. If correct, this raises the possibility that the G1-less cell cycle of *Dictyostelium*, in which RblA directs the major cell-cycle events, resembles that of a much earlier basal eukaryotic form.

## Materials and Methods

### Preparation of Cells

*Dictyostelium discoideum* strain Ax2 was used for all experiments described here. The *rblA* disruptant has been described previously [4]. Vegetative cells were grown in HL5 and harvested at a density of 2×10^6^ cells ml^−1^. For development cells were dispersed on Whatman 50 filters overlaid on Millipore absorbent pads moistened with LPS [61]. Aggregates were harvested at the early culminant stage just after the spore mass lifted off the substrate and the basal disk became visible; this occurred at about 19 hours in the wild type and 16 hours in the *rblA* disruptant.

Cold synchronization was performed as described [24]. Briefly, cultures between 0.5–1×10^6^ cells ml^−1^ were incubated for 16 hours at 9.5°C then rapidly warmed to 22°C in a 40°C water bath. Changes in culture temperature were followed using an ethanol-washed thermometer. Cell proliferation was monitored using a Beckman-Coulter Z2 particle analyser; BrdU labelling was conducted as described [1], [13]. BrdU-incorporated cells were counted automatically using a custom-written ImageJ macro.

### Isolation of RNA, Microarray Analysis, Real-time PCR Validation, and Sequence Acquisition

Cells were snap-frozen in liquid nitrogen (mutant/parent comparison) or fixed in RNALater® (Ambion) at harvesting. RNA was extracted using Bio-Rad Aurum total RNA spin columns following the manufacturer’s protocol. To minimize column clogging, lysates were drawn-through a 23-gauge needle prior to loading. Samples were labelled for microarray analysis, hybridized to arrays, and data processed as described previously [7]. Three biological and two technical replicates were conducted. The MIAME compliant array data have been deposited in ArrayExpress with accession number E-TABM-1087. Two S-phase genes found to be upregulated in the *rblA* disruptant and two genes that showed no difference in expression between the mutant and parental strain were selected for real-time PCR analysis (see **Table S1**). Primers were designed using the Primer Express Software (Applied Biosystems) and cross-checked with the Finnzymes Multiple Primer Analyzer program. cDNAs were generated from 100 nanograms of the same developmental RNA employed in the microarrays experiments using a RevertAid H Minus First Strand cDNA Synthesis Kit (Fermentas). qPCR reactions were done in triplicate on biological duplicates using SYBR Green PCR mix (Applied Biosystems) and 0.3 micromolars of each oligonucleotide. Efficiencies were determined using a pool of the mutant and wild type cDNAs on five 5-log dilutions. Dissociation curves showed the absence of non-specific products (not shown). The mRNA-Seq protocol of the Illumina/Solexa platform was used for whole transcriptome sequencing at the DNA Core Facility of the University of Missouri in Columbia or at the McGill University/Genome Quebec Innovation Centre in Montreal. Reads of 42 bp (wild type/mutant comparison) or 36 bp (cell-cycle experiment) were obtained. Two biological replicates were conducted for the wild type and rblA-null mutant. Three samples from the cell-cycle synchronization experiment (0 hr, 1 hr, and 3 hr) were sequenced twice and served as technical replicates.

### Processing of Sequence Data

A transcriptome consisting of all predicted protein coding sequences was obtained from www.Dictybase.org; sequences for rRNA intersegments were added. The sequence of DDB_G0270824 which contains homologies to both *tdd-4* and *thugS* transposable elements was split to prevent fusion of the corresponding “gene collectives” (see below).

mRNA-Seq reads were mapped to transcriptional units using software written specifically for this project. The software incorporates two features that are not yet widely used in RNA-Seq read mapping. (1) It eliminates all targets from the mapping process that are identical to other potential targets in all but one base. This obviates mismapping due to single-base sequencing errors. (2) Groups of near-identical genes are recognized automatically and organized into “collectives”. Reads that could be mapped to the collective but not to an individual gene were assigned to the “collective gene”. All reads that could be assigned uniquely to a gene within the collective continued to be so. Among other things, this procedure allowed us to monitor transposon-coded genes, some of which turned out to be very strongly regulated by RblA.

Our software uses a straightforward implementation of open hash tables for mapping. It is implemented in Java and designed so that all data structures for the *Dictyostelium* transcriptome fit in about 1 GB of main memory, allowing fast execution (on the order of minutes) on standard PCs.

The software executes in five phases:

1. Index preparation: As a first step, the transcriptome is logically segmented into 42-base or 36-base potential mapping targets (“snippets”). A status array is constructed with one entry for each target. Snippets are represented as an integer which is their index in the transcriptome: snippet 0 starts at the beginning of the first gene, etc. In phases II-V, these indexes are placed into different hash tables. An additional array is constructed, mapping snippet indexes back to their gene numbers.
2. Intra-gene uniqueness tests: within each gene, all snippets that are identical to other snippets within the same gene are marked as non-mappable within the status array. This uses a small hash table for each gene. This test eliminates the simplest sequences, such as extended trinucleotide repeats, from further processing.
3. [LOOSER]Collective building: all remaining snippets are compared with each other by entering them all into a large hash table. When entering a snippet into the hash table, the algorithm detects whether an identical snippet has already been put there. After the processing of phase II, such a match necessarily must come from a different gene. The number of matches for each pair of genes is tallied in a sparse matrix. If a pair of genes contains more than 50% identical snippets and the two gene lengths differ by no more than a factor of 2, a “collective” is created for this pair of genes. The status and index arrays from phase I are extended to incorporate the new collective genes. 297 collective genes were created by the algorithm, each containing between 2 to 53 genes.
4. Robust uniqueness tests: All snippets that are still marked as mappable are now compared with each other by entering them into a hash table. A snippet that occurs within two or more genes (that are not part of the same collective) is marked as non-mappable in the status array. This process is run 43 times, using 43 slightly different hash and comparison functions. First, only identical snippets are marked as non-mappable. Second, snippets that differ only in the first base are marked, then, those that differ only in the second, and so forth. The remaining snippets are considered to be “robustly unique” and can be used for mapping.
5. Read mapping: all reads are mapped to the remaining snippets in the transcriptome, creating a number of matches per gene and a profile of the match over each gene’s length. The matching process uses the large hash table again. Only exact matches are counted.

The source code is available from the authors on request.

The mRNA-Seq data had been deposited in Gene Expression Omnibus [62] and is available through GEO series accession number GSE30368 http://www.ncbi.nlm.nih.gov/geo/query/acc.cgi?acc=GSE30368.

### Selection of *rblA* Repressed Genes for Detailed Discussion

For all genes, we first calculated normalized hits in each experiment by dividing the number of mappable reads for a particular gene by the total number of fitted reads for the sample. Mutant-specific regulation factors were defined as normalized hits in the *rblA* disruptant divided by the normalized hits to the wild type; these were calculated separately for growing and developing cells using averages of the two biological replicates. The logarithms (base 2) of the growth phase and development-specific regulation factors were then plotted against one another. Two thirds of the points lay within a distance of 0.5 of the origin, corresponding to upregulation or downregulation of 40%. The diagram also revealed a clearly visible subgrouping of points in the northeast quadrant, corresponding to genes that are upregulated in the *rblA* disruptant in both growth and development. Depending on the exact definition of its boundaries, this cluster contains 3–5% of *Dictyostelium* transcript models. Transcripts or collectives with linear induction factors of 2 or more were selected for closer examination. For each of these, a statistical test using edgeR [63] was performed of the null hypothesis that the observed differences could occur by chance. Genes with adjusted *p*-values of <0.05 were considered to be RblA-regulated.

### Data Normalization

Regulation factors presented in this manuscript are expressed as reads per kilobase of exon model per million mappable reads [64]. These were normalized to give a total value of 1 by adding the reads for a particular gene over the entire series (cell-cycle synchronization or developmental time course) and dividing the reads from a given time point by this sum to produce the regulation factor.

### Analytical Strategy

Genes selected as above were first analysed by using the gene-ontology tool GOAT. This procedure revealed little interesting substructure, probably because the cell cycle genes of *Dictyostelium* have not yet been systematically annotated. We then conducted BLAST and literature searches for most of the genes. Many previously unrecognized *Dictyostelium* cell-cycle genes were uncovered in this process (http://wiki.dictybase.org/dictywiki/index.php/MacWilliams_contributions).

## Supporting Information

Figure S1 **Fundamental agreement between microarray and mRNA-Seq data.** Compared are the data for developing cells, where the microarray signals were clearest. Normalized fold-change ratios (*rblA* disruptant mutant/AX2 parental strain) were compared and visualized as scatterplots. The abundance of a particular transcript measured by microarray (x-axis) compared to the abundance of the same transcript measured by mRNA-Seq (y-axis). The linear regression line is in orange and Pearson’s correlation coefficient (*r*) is included in the upper, left-hand corner of each graph. (A) Comparison of the 20 most abundant transcripts. (B) Comparison of 247 genes considered to be strongly differentially expressed between samples in the microarray analysis. (C) Correlation of 175 genes scored as significant (p<0.05) in the mRNA-Seq data with the corresponding microarray measurements.
(TIF)

Table S1 **qPCR validation on developmental RNA**
(DOC).

Table S2 **Microsoft Excel file containing data on** ***rblA*** **-repressed genes as well as a number of additional genes included for comparison.** This table contains raw data on regulation by RblA, developmental regulation, and regulation in the cold-synchronization experiment. Macros are included to assist the user in graphing the expression profiles and calculating error bars (the confidence limits are user-settable). Gene IDs are linked to DictyBase.
(XLS)
