# Supplementary figures and images for: A Retinoblastoma Orthologue Is a Major Regulator of S-Phase, Mitotic, and Developmental Gene Expression in Dictyostelium
Source: PLoS One. 2012 Jun 29;7(6):e39914. doi: 10.1371/journal.pone.0039914 (PMC3386910; doi:10.1371/journal.pone.0039914)

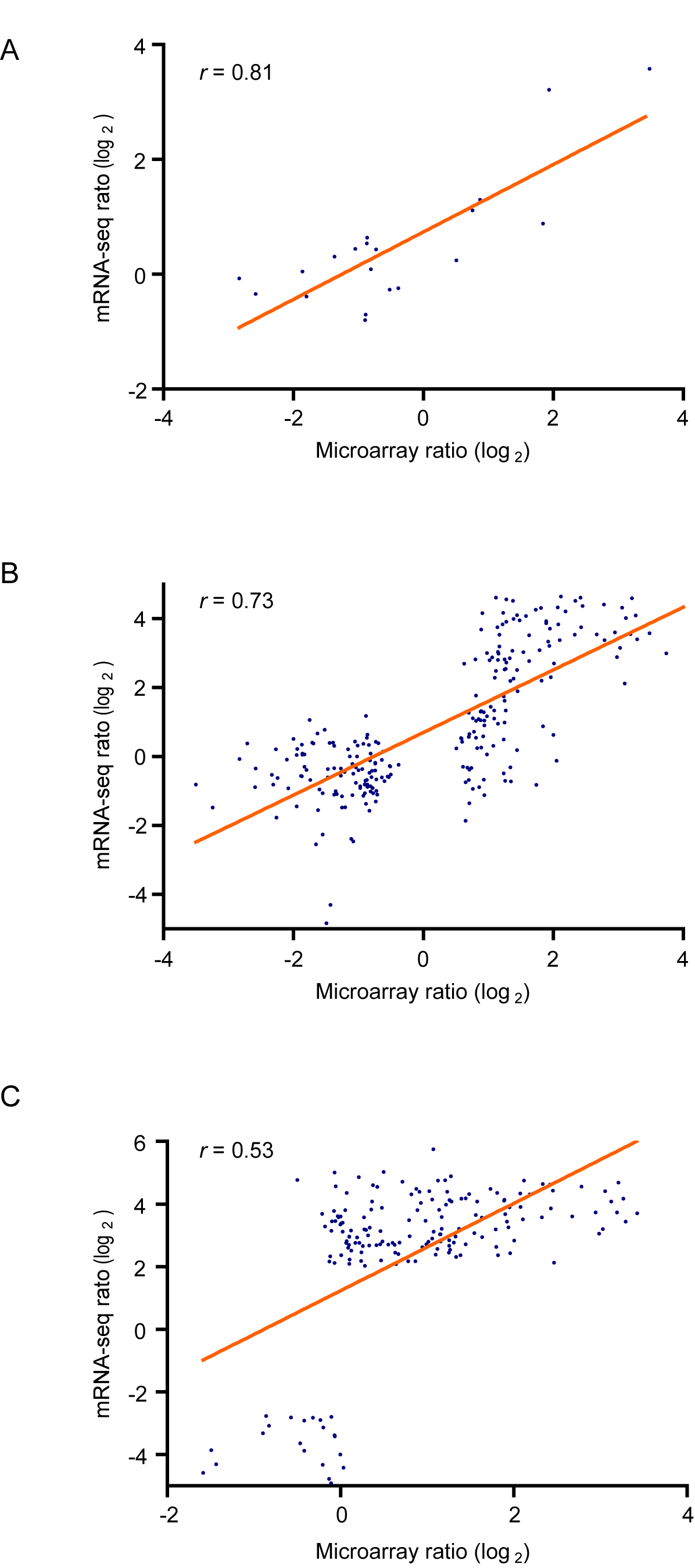

Supplement: Figure S1 — Fundamental agreement between microarray and mRNA-Seq data. Compared are the data for developing cells, where the microarray signals were clearest. Normalized fold-change ratios (rblA disruptant mutant/AX2 parental strain) were compared and visualized as scatterplots. The abundance of a particular transcript measured by microarray (x-axis) compared to the abundance of the same transcript measured by mRNA-Seq (y-axis). The linear regression line is in orange and Pearson’s correlation coefficient (r) is included in the upper, left-hand corner of each graph. (A) Comparison of the 20 most abundant transcripts. (B) Comparison of 247 genes considered to be strongly differentially expressed between samples in the microarray analysis. (C) Correlation of 175 genes scored as significant (p<0.05) in the mRNA-Seq data with the corresponding microarray measurements. (TIF) [file pone.0039914.s001.tif]
