# Supplementary material for: A Retinoblastoma Orthologue Is a Major Regulator of S-Phase, Mitotic, and Developmental Gene Expression in Dictyostelium
Source: PLoS One. 2012 Jun 29;7(6):e39914. doi: 10.1371/journal.pone.0039914 (PMC3386910; doi:10.1371/journal.pone.0039914)
Supplement: Supplementary file 2 [file pone.0039914.s002.doc]

## Sheet: instructions for use

	Table S1 contains raw data on genes discussed in this paper The data are on the next tab (tabs are on the lower left of the screen)  To aid in visualizing the data, this sheet incorporates macros The raw data is accessible without macros If you wish to use the graphing  feature, you will probably need to change your Excel security settings  (go to Extras-Macros-Security, check "Intermediate"); then close and re-open the spreadsheet)  To graph the data for any gene,  click on the Gene Name, then press <cntrl g>     (g stands for graph) To graph the next gene,                 click on the Gene Name, then press <cntrl n>      (n stands for next) To graph the previous gene,      click on the Gene Name,  then press <cntrl p>     (p stands for previous)  To search for a gene of interest,  enter the Gene Name in the search box, then press <ENTER> and >cntrl f>  (f stands for find) To return to the search box, press <cntrl t>  (t stands for top)  The error bars are calculated as confidence intervals for the Poisson distribution (see link below) The pre-set confidence level is  0.95, which corresponds roughly to "plus or minus two standard deviations" You may reset the confidence level. Higher confidence gives larger error bars The error bars will disappear if you set the confidence at 0 The spread sheet works correctly up to confidence level 0.99; at higher confidence levels       problems with the approximations cause unrealistic unsymmetrical error bars Setting the level at 1 causes an algorithm to fail and the bars will not be displayed correctly  This spreadsheet is write-protected to avoid accidents   To unprotect, go to Extras-Sheet-Protection.  No password is necessary
	confidence intervals for Poisson
	Instructions for Macintosh users  This spread sheet should run as described above if you have Office 2004 for Mac. If you have this office version, just follow the directions in the top box  Office 2008 for Mac does not run Visual Basic macros.  Users are invited to use two AppleScript files (available below).  Please store these scripts in the file  /Users/your Username/Documents/Microsoft User Data/Excel Script Menu Items/  When you open Excel, these scripts should appear as menu items in the "Automator" (just to the right of the Help menu)  We suggest that you first scroll to a gene that interests you, click on the gene name, and start the script "graph this gene". Graphs showing the expression in development, in the cell cycle, and in the rblA disruptant should appear at the bottom of the screen  You can also search for a gene  by typing the name of the gene in cell B1 and starting the script "table1_search_editable"  These scripts will not work with some older versions of the AppleScript Editor or the AppleScript Executer   You may have to update these files.  This update should be free 
	 table1_search_editable.scpt
	graph this gene.scpt

## Sheet: rblA_regulated_genes

FIND:	siva
	Gene Name ----------------- grey indicates gene whose transcript is not substantially regulated by RblA but is included for comparison
gene/protein identifiers		expression summary						Functional summary		Data on expression in rblA disruptant														Data from developmental time course																Data from cell-cycle synchronization by incubation in cold
DDB_G0 ID (hyperlinked to Dictybase)	Gene Name ----------------- grey indicates gene whose transcript is not substantially regulated by RblA but is included for comparison	developmental expression ------------------------- green indicates pattern typical for cell cycle genes	cell-cycle regulation -------------------------- green indicates pattern typical for cell cycle genes	time of overexpression in rblA disruptant -------------------------- green indicates pattern typical for cell cycle genes	Fold repression by RblA in wild-type cells during growth	Fold repression by RblA in wildtype cells during development		function	comment (click to read)	mappable reads wt growth phase biological 1	mappable reads wt growth phase biological 2	mappable reads rblA- growth phase biological 1	mappable reads rblA- growth phase biological 2	mappable reads wt development biological 1	mappable reads wt development biological 2	mappable reads rblA- development biological 1	mappable reads rblA- development biological 2	total mappable reads for a particular gene wt	total mappable reads for a particular gene rblA-	p for null hypothesis (no overall effect of rblA) development	p for null hypothesis (no overall effect of rblA) growth			mappable reads 0h biological 1	mappable reads 0h biological 2	mappable reads 4h biological 1	mappable reads 4h biological 2	mappable reads 8h biological 1	mappable reads 8h biological 2	mappable reads 12h biological 1	mappable reads 12h biological 2	mappable reads 16h biological 1	mappable reads 16h biological 2	mappable reads 20h biological 1	mappable reads 20h biological 2	mappable reads 24h biological 1	mappable reads 24h biological 2	total mappable reads for a particular gene		mappable reads asynchronous	mappable reads 0h	mappable reads 0h technical replicate	mappable reads 1h	mappable reads 1h technical replicate1	mappable reads 1h technical replicate2	mappable reads 2h	mappable reads 3h	mappable reads 3h technical replicate	mappable reads 4h
DDB_G0272813	cdk1	max mid devel	max late G2	growth&devel	2.71	10.81		Overall cell-cycle control	cyclin-dependent kinase 1, master mitotic regulator	271	4289	963	4739	151	864	2010	9644	5575	17356	0.000190219438663202	0.999998877031569			63	24	144	444	217	465	391	474	199	382	31	132	74	181	3221		549	255	188	942	476	2014	493	342	159	289
DDB_G0271642	cks1	max mid devel	max late G2	devel only	1.45	4.47	cell cycle overall control	cdk-associated protein, essential part of catalytic complex		66	489	122	300	22	72	139	9644	649	10205	0.0438425251395856	0.999998877031569			1	8	6	41	58	134	105	180	49	110	9	37	16	34	788		61	37	30	97	54	234	57	62	21	48
DDB_G0279343	aurK	max mid devel	max late G2	growth&devel	2.61	6.57		Overall cell-cycle control	Single aurora kinase in Dictyostelium with properties of both auroraA and auroraB of metazoans, see Li et al, Eukaryotic Cell 7, 894-905 (2008); it associates with spindle poles, centromeres, and central spindle	45	641	131	814	0	161	149	699	847	1793	0.0189960693131977	0.999998877031569			4	1	2	7	17	46	93	109	74	138	8	12	3	6	520		78	49	32	161	81	322	93	56	20	45
DDB_G0291133	DDB_G0291133 (wee1)	max mid devel	max late G2	growth&devel	4.92	6.5		Overall cell-cycle control	wee kinase	9	113	50	267	15	61	103	497	198	917	0.00458741605200068	0.456246821830309			0	2	4	14	30	78	55	95	27	62	4	20	17	18	426		25	19	21	34	22	64	19	6	1	12
DDB_G0291842	DDB_G0291842 (wee1)	"repair"	max late G2	growth&devel	2.69	5.21		Overall cell-cycle control	similar to wee kinase; developmental time course typical of DNA repair genes suggesting role in damage-induced G2/M arrest	76	485	240	600	234	722	1667	3176	1517	5683	0.014235313011569	0.999998877031569			765	320	93	224	208	838	896	1026	362	1350	146	655	167	511	7561		101	73	46	173	79	332	121	98	36	77
DDB_G0275983	cdc6	max mid devel	max late G2	growth&devel	4.8	9.22		DNA replication/initiation	replication licensing factor	1	85	7	143	3	26	25	348	115	523	0.000707109069285621	0.999998877031569			2	5	1	0	12	18	16	32	25	21	4	5	4	4	149		7	6	12	14	10	34	3	1	1	4
DDB_G0267748	cdc45	max mid devel	max late G2	growth&devel	4.44	7.68		DNA replication/initiation	required for the initiation of DNA replication 	55	535	303	1036	34	206	389	1108	830	2836	0.00372580907885086	0.476050529833625			0	1	6	4	36	98	121	225	115	201	15	23	5	9	859		134	89	53	263	140	605	135	106	45	86
DDB_G0283073	orcA	late devel	max late G2	growth only	2.99	1.6		DNA replication/initiation	origin recognition complex subunit 1	10	164	34	234	21	126	45	181	321	494	1	0.999998877031569			10	0	8	21	14	58	26	61	31	53	29	75	31	53	470		31	24	11	49	21	109	23	23	8	15
DDB_G0269894	orcB	"repair"	flat	not substantially RblA regulated	1.6	1.64		DNA replication/initiation	origin recognition complex subunit 2, included here for comparison	19	138	38	96	3	21	8	18	181	160	1	0.999998877031569			29	12	1	0	2	4	9	10	5	8	4	0	1	8	93		26	11	5	17	9	26	10	10	4	13
DDB_G0271078	orcC	max mid devel	max late G2	growth&devel	1.97	3.45		DNA replication/initiation	origin recognition complex subunit 3	17	191	21	244	13	80	43	318	301	626	0.134507405954735	0.999998877031569			2	6	1	11	31	65	75	121	56	86	8	13	10	18	503		36	19	11	45	23	122	23	20	5	25
DDB_G0271562	orcD	max mid devel	flat	growth&devel	2.66	4.03		DNA replication/initiation	origin recognition complex subunit 4.  This subunit is responsible for the DNA binding of the complex, see Chuang et al, JBC 277, 16920-16927 (2002), Kong and DePamphilis, Mol Cell Biol 21, 8095-8103 (2001), also Stefanovic et al, JBC 278, 42737-42743 (2003)	0	25	7	78	1	5	15	117	31	217	0.000322866520651945	0.31708672093985			0	0	0	1	1	7	6	15	7	7	0	0	0	1	45		4	1	0	2	0	5	1	0	1	1
DDB_G0293214	orcE	max mid devel	max late G2	devel only	1.94	2.17		DNA replication/initiation	origin recognition complex subunit 5	5	113	9	123	16	132	37	356	266	525	0.568453103939695	0.999998877031569			1	2	12	41	57	46	93	48	38	25	9	25	24	22	443		9	7	6	14	14	30	7	8	2	7
DDB_G0284771	orcF	max mid devel	max late G2	growth&devel	2.63	2.38		DNA replication/initiation	origin recognition complex subunit 6	13	108	31	162	14	52	33	166	187	392	0.498115112889929	0.999998877031569			7	2	2	9	10	21	29	50	20	64	12	33	2	7	268		16	3	4	19	9	24	7	10	4	5
DDB_G0290331	ssrp1	"repair"	max late G2	growth&devel	2.08	5.17	DNA replication/chromatin disassembly	subunit of FACT complex, destabilizes nucleosomes and facilitates DNA-templated processes such as  transcription, replication and repair		224	2235	560	2109	59	315	452	1158	2833	4279	0.027690226421766	0.999998877031569			172	46	28	94	112	409	238	345	124	341	25	126	39	102	2201		416	175	120	459	249	954	291	258	92	239
DDB_G0289271	gins1	max mid devel	max late G2	growth&devel	3.98	15.45		DNA replication/replication fork	go ich ni san complex subunit 1, interacts with Cdc45 and the MCM complex	2	46	8	97	1	17	19	228	66	352	0.000490396303027045	0.946983903472693			1	0	0	0	2	5	6	10	4	11	0	3	1	1	44		5	4	2	10	5	22	3	3	0	2
DDB_G0293564	gins2	max mid devel	max late G2	growth&devel	4.74	16.09		DNA replication/replication fork	go ich ni san complex subunit 2,  interacts with Cdc45 and the MCM complex	1	54	6	88	1	14	14	265	70	373	0.0000744287663486653	0.999998877031569			0	0	0	0	1	7	4	6	9	5	0	1	0	0	33		2	0	4	6	5	17	1	0	0	2
DDB_G0291506	gins3	max mid devel	max late G2	growth&devel	5	3.64		DNA replication/replication fork	go ich ni san complex subunit 3,  interacts with Cdc45 and the MCM complex	2	45	10	120	4	52	16	254	103	400	0.0863834789295834	0.576530873988985			0	0	0	7	5	7	18	30	17	11	12	11	9	11	138		5	6	4	12	5	27	4	0	0	1
DDB_G0269324	gins4	max mid devel	too little data	growth&devel	2.9	4.52		DNA replication/replication fork	go ich ni san complex subunit 3,  interacts with Cdc45 and the MCM complex	1	20	1	46	0	4	1	42	25	90	0.0379521742964718	0.999998877031569			0	0	0	1	0	1	5	6	5	4	1	2	0	1	26		0	0	0	2	1	2	2	2	1	0
DDB_G0286623	mcm2	max mid devel	max late G2	growth&devel	4.13	23.46		DNA replication/replication fork	DNA helicase	123	673	560	1367	63	249	2148	4373	1108	8448	0.00000258780179535586	0.51012488409961			116	64	32	79	235	650	388	599	209	567	49	145	49	142	3324		225	182	88	306	139	611	130	147	60	109
DDB_G0280309	mcm3	growth and terminal	max late G2	growth&devel	5.07	13.35		DNA replication/replication fork	DNA helicase	31	295	165	768	115	320	1737	4956	761	7626	0.0000494986530932022	0.314619138937411			1707	919	552	1288	172	530	527	584	908	2106	976	3202	1125	4430	19026		109	72	46	145	63	252	52	55	24	42
DDB_G0275623	mcm4	max mid devel	max late G2	growth&devel	3.04	6.5		DNA replication/replication fork	DNA helicase	44	399	151	585	13	53	110	324	509	1170	0.00673384748371184	0.999998877031569			5	3	2	3	21	61	52	89	37	64	1	8	1	3	350		90	51	33	147	80	352	67	61	24	47
DDB_G0292958	mcm5	max mid devel	max late G2	growth&devel	2.99	12.74		DNA replication/replication fork	DNA helicase	81	522	257	793	37	160	667	1627	800	3344	0.000105233936336663	0.999998877031569			14	11	6	15	63	206	202	250	124	278	19	60	24	71	1343		158	78	58	238	139	540	110	96	45	68
DDB_G0272760	mcm6	max mid devel	max late G2	growth&devel	3.72	11.43		DNA replication/replication fork	DNA helicase	45	497	174	958	19	96	328	842	657	2302	0.000262671659497507	0.756878868018633			33	9	6	14	26	105	118	174	51	137	5	22	2	16	718		160	141	66	223	112	431	124	73	45	71
DDB_G0282933	mcm7	max mid devel	max late G2	growth&devel	3.57	22.36		DNA replication/replication fork	DNA helicase	102	695	413	1191	34	196	1172	2784	1027	5560	0.00000539282314761753	0.766169877351635			4	3	2	14	76	203	172	273	101	206	2	20	2	11	1089		163	147	94	166	95	446	103	103	61	79
DDB_G0287103	DDB_G0287103 (Timeless)	max mid devel	max late G2	growth&devel	3.19	13.72		DNA replication/replication fork	similar to timeless, component of unperturbed (undamaged) replisomes, thought to couple polymerase to helicase activity, knockdown leads to accumulation of ssDNA at replication forks, see Smith et al,  Journal of Cell Biology 187, 15-23 (2009)	17	801	73	1011	16	228	297	2742	1062	4123	0.000116159994529943	0.999998877031569			0	2	6	8	49	126	121	219	117	120	6	15	15	38	842		68	56	38	121	79	289	74	35	20	44
DDB_G0284731	DDB_G0284731	max mid devel	max late G2	growth&devel	3.28	12.1		DNA replication/replication fork	weakly similar (mutual best BLAST hit) to replication fork protection component Swi3 of fission yeast;  the yeast protein acts in a complex with Swi1, which is similar to Dictyostelium timeless 	11	187	28	381	9	47	122	668	254	1199	0.000142398697912549	0.986750301142353			2	3	1	10	16	49	53	67	34	46	5	16	6	14	322		20	20	10	39	11	61	11	12	4	13
DDB_G0282191	polA1	max mid devel	max late G2	growth&devel	4.66	23.7		DNA replication/replication fork	DNA polymerase alpha, catalytic subunit	161	969	810	2262	80	354	2770	6187	1564	12029	0.00000258780179535586	0.374959941644424			13	4	3	24	144	463	347	594	212	515	30	77	26	73	2525		314	199	121	480	223	931	229	233	66	147
DDB_G0282731	polA2	max mid devel	max late G2	growth&devel	3.81	27.2		DNA replication/replication fork	DNA polymerase alpha, B subunit	50	417	134	1035	15	136	569	3022	618	4760	0.00000246285356288029	0.766869313614828			5	2	4	12	41	110	77	149	65	116	4	19	4	17	625		54	31	29	116	49	256	39	44	17	31
DDB_G0270442	polA3	max mid devel	max late G2	growth&devel	3.04	14.97		DNA replication/replication fork	DNA polymerase alpha, primase large subunit	34	487	110	749	11	74	218	1012	606	2089	0.0000618549726174443	0.999998877031569			0	0	0	6	11	33	51	90	38	73	1	8	0	4	315		39	33	25	144	78	290	47	29	16	31
DDB_G0285853	polA4	max mid devel	max late G2	growth&devel	3.41	15.45		DNA replication/replication fork	DNA polymerase alpha, primase small subunit	20	399	79	640	5	57	120	546	481	1385	0.000253236273024119	0.999998877031569			1	1	0	5	11	40	27	66	28	57	2	8	1	4	251		58	44	21	99	49	198	52	38	24	29
DDB_G0285381	polD1	max mid devel	max late G2	growth&devel	4.07	13.47		DNA replication/replication fork	DNA polymerase delta, catalytic subunit	138	1243	644	2394	73	362	1390	3969	1816	8397	0.0000732758777499943	0.566158554064673			1	1	0	5	11	40	27	66	28	57	2	8	1	4	251		345	227	189	415	238	943	264	209	87	193
DDB_G0281641	polD2	mid + late devel	max late G2	growth&devel	3.15	8.42		DNA replication/replication fork	DNA polymerase delta, subunit 2	37	418	132	631	47	121	303	1657	623	2723	0.000963210082822998	0.999998877031569			5	5	2	14	35	77	72	102	43	84	30	81	23	62	635		78	57	35	101	56	245	48	43	25	31
DDB_G0294609	polD3	max mid devel	max late G2	growth&devel	2.94	8.63		DNA replication/replication fork	weakly similar to DNA polymerase delta subunit 3 of animals and plants; has conserved PIP domain	17	740	61	970	12	132	134	1069	901	2234	0.00161906201861506	0.999998877031569			1	1	8	6	22	85	63	102	55	80	5	18	13	24	483		47	26	18	105	53	224	34	31	11	29
DDB_G0272158	wrnip1	"repair"	max late G2	growth&devel	3.94	12.12		DNA replication/replication fork	Werner helicase interacting protein, modulator for initiation by DNA polymerase delta (Sidrova, DNA Repair (Amst) 7, 1776-1786 (2008))	27	162	126	293	26	62	388	775	277	1582	0.000109635585999639	0.599775578844902			32	56	10	19	36	130	103	140	36	100	3	15	9	23	712		63	55	41	85	42	181	32	44	18	20
DDB_G0283189	DDB_G0283189 (polymerase epsilon subunit A)	max mid devel	max late G2	growth&devel	6.2	11.9		DNA replication/replication fork	similar to DNA polymerase epsilon subunit A in yeast and vertebrates; thought to be involved in DNA replication, DNA repair, and cell cycle checkpoint control in eukaryotes (Dictybase)	37	628	280	1733	109	526	1617	6334	1300	9964	0.000110485980318848	0.174199622217677			4	2	19	39	181	381	354	564	337	506	59	147	68	186	2847		196	136	96	286	169	637	131	90	44	76
DDB_G0278367	DDB_G0278367 (polymerase epsilon subunitB)	max mid devel	max late G2	growth&devel	3.42	6.98		DNA replication/replication fork	putative DNA polymerase epsilon subunit B (Dictybase)	6	228	25	348	8	44	56	406	286	835	0.00337338954826548	0.999998877031569			0	2	0	8	7	35	32	59	30	29	4	5	2	5	218		31	22	17	33	22	101	12	19	3	12
DDB_G0284987	repG	"repair"	max late G2	growth&devel	2.63	13.31		DNA replication/replication fork	flap endonuclease	30	668	94	802	14	134	268	1369	846	2533	0.000185824291492599	0.999998877031569			41	34	9	20	43	108	84	124	57	111	7	22	7	27	694		79	70	59	176	93	385	91	60	31	51
DDB_G0274493	lig1	mid + late devel	max late G2	growth&devel	3.05	21.26		DNA replication/replication fork	replicative DNA ligase	151	1863	557	2546	91	584	2637	10727	2689	16467	0.00000450187385662992	0.999998877031569			12	13	15	35	86	214	229	356	203	336	62	137	120	253	2071		204	169	110	268	155	595	148	116	42	104
DDB_G0267916	mtmr15	max mid devel	max late G2	growth&devel	4.08	6.99		DNA replication/intrastrand crosslink repair	similar to myotubularin related-protein 15 of metazoans; renamed FAN1 in humans because it interacts with FANCD2 in repair of intrastrand crosslinks, see O'Donnel and Durocher, Molecular Cell 39, 167-169 (2010)	9	118	42	229	10	35	70	324	172	665	0.0039197355659298	0.714651503573401			0	2	4	9	21	29	33	55	35	44	5	10	9	10	266		28	10	11	36	13	57	17	3	2	10
DDB_G0270418	top2	"repair"	max late G2	growth&devel	4.72	9.18		DNA replication/topological resolution	Dicty has 4 topoisomerases;  only top2 and top2mt can introduce transient double-strand breaks and thus resolve topological problems. This activity is required to separate newly-replicated DNA molecules, see Nitiss, Nature Reviews Cancer 9, 327-337 (2009)	248	2535	1302	5830	147	545	1711	4906	3475	13749	0.000501872214779555	0.378459342306611			116	67	17	42	169	524	196	494	225	344	176	302	61	281	3014		574	507	333	906	518	2040	504	393	178	339
DDB_G0287607	pcna	"repair"	max late G2	growth&devel	4.23	8.31		DNA replication/replication fork	"sliding clamp"; binding platform for postreplication repair enzymes	410	4972	2192	8853	95	607	1210	3592	6084	15847	0.00184462498298609	0.524325434677187			100	120	23	47	113	354	421	540	247	495	23	64	13	49	2609		722	279	213	855	439	1768	628	504	262	476
DDB_G0285961	rfc1	max mid devel	max late G2	growth&devel	2.19	3.79		DNA replication/replication fork	PCNA/9-1-1 clamp loader complex	146	1107	327	1269	72	440	407	1158	1765	3161	0.139449145640835	0.999998877031569			30	20	40	139	234	588	242	382	147	249	57	114	128	321	2691		309	194	145	331	203	774	190	160	83	131
DDB_G0291868	rfc2	"repair"	max late G2	growth&devel	3.41	17.2		DNA replication/replication fork	PCNA/9-1-1 clamp loader complex	66	594	275	915	11	64	294	683	735	2167	0.0000462382205740443	0.889782542581923			22	6	6	5	22	71	72	96	38	131	1	12	5	8	495		160	67	47	190	97	369	84	94	36	77
DDB_G0293702	rfc3	max mid devel	max late G2	growth&devel	2.82	6.17		DNA replication/replication fork	PCNA/9-1-1 clamp loader complex	53	429	169	583	20	45	143	309	547	1204	0.00680063139163337	0.999998877031569			6	6	5	6	18	83	81	93	26	62	3	5	0	2	396		90	72	40	151	54	225	62	59	23	43
DDB_G0286027	rfc4	max mid devel	max late G2	growth&devel	2.84	26.81		DNA replication/replication fork	PCNA/9-1-1 clamp loader complex	45	410	143	562	8	57	296	1277	520	2278	0.00000267889915379522	0.999998877031569			3	2	2	13	26	78	70	84	39	80	23	45	8	27	500		69	28	19	122	67	278	53	46	24	31
DDB_G0282235	rfc5	max mid devel	max late G2	growth&devel	2.63	8.21		DNA replication/replication fork	PCNA/9-1-1 clamp loader complex	15	267	31	434	7	45	54	502	334	1021	0.00115758081047498	0.999998877031569			0	0	1	4	15	21	31	42	27	25	3	7	7	9	192		31	20	14	45	23	97	23	13	5	16
DDB_G0281907	DDB_G0281907 (ELG1)	max mid devel	max late G2	growth&devel	3.85	12.25		DNA replication/replication fork	putative AAA-ATPase weakly similar to  ELG1, an alternative replication factor C large subunit that is required for S-phase progression in yeast. Recent publications stress a role in double-strand break repair, see Ogiwara et al, NAR 35, 353-362 (2007), but the gene is strongly cell-cycle regulated, unlike most ds repair genes; developmental expression not typical of repair gene	10	172	42	349	8	57	123	690	247	1204	0.000193042932112259	0.778223695056695			3	0	3	9	10	38	14	54	26	17	2	4	3	12	195		20	18	2	36	16	73	14	11	3	10
DDB_G0267904	DDB_G0267904 (ctf4)	max mid devel	max late G2	growth&devel	4.5	17.98		DNA replication/replication fork	wd-40 repeat and HMG-box DNA-binding protein, similar (mutual best BLAST hit) to chromosome transmission fidelity protein 4, aka CTF4/AND-1.  AND-1 is a replisome protein involved in the initiation of DNA replication; it participates in loading MCM2-7 helicase 	65	586	314	1329	34	249	911	3127	934	5681	0.0000256301396017297	0.459521733171895			6	4	0	8	77	167	204	248	121	232	9	24	9	14	1123		128	60	47	260	148	513	131	95	37	71
DDB_G0271400	dcc1	max mid devel	max late G2	growth&devel	4.7	22.65		DNA replication/replication fork	orthologue of DCC1, which forms a complex with CTF8 and CTF18; this is involved in PCNA-loading or unloading and is required for sister chromatid cohesion; may allow the replication fork to pass through established cohesin rings; see Farina et al, Journal of Biological Chemistry 283, 20925-20936 	8	102	41	238	2	20	66	337	132	682	0.000039500692434149	0.492536516855637			0	0	0	2	15	13	18	27	11	12	3	2	2	1	106		10	2	3	19	20	31	9	2	6	3
DDB_G0287975	DDB_G0287975 (ctf8)	max mid devel	too little data	growth&devel	6.16	9.11		DNA replication/replication fork	similar to CTF8, which forms a complex with CTF18 and DCC1; which is involved in PCNA-loading or unloading and is required for sister chromatid cohesion; may allow the replication fork to pass through established cohesin rings; see Farina et al, Journal of Biological Chemistry 283, 20925-20936 (2008) 	1	60	10	105	0	19	15	87	80	217	0.0348947909409979	0.97977504912094			0	0	0	2	5	12	4	14	7	9	0	1	1	0	55		1	3	3	4	4	21	2	1	2	4
DDB_G0280397	DDB_G0280397	max mid devel	max late G2	growth&devel	5.07	15.64		DNA replication/post-replication repair	contains weak homology to prokaryotic SMC N-terminal domain found in RecF, RecN.  RecF is required for re-assembly of the replication fork after it stalls at a thymidine dimer, see Courcelle et al, PNAS 94, 3714-3719 (1997).  RecN is involved in double strand break repair in bacteria	13	294	99	497	12	91	275	1014	410	1885	0.0000756508358561478	0.559552794731648			1	1	0	5	11	40	27	66	28	57	2	8	1	4	251		43	27	11	46	25	114	30	17	7	16
DDB_G0284281	DDB_G0284281	mid + late devel	max late G2	growth&devel	2.52	8.46		DNA replication/post-replication repair	contains weak homology to prokaryotic SMC N-terminal domain found in RecF, RecN.  RecF is required for re-assembly of the replication fork after it stalls at a thymidine dimer, see Courcelle et al, PNAS 94, 3714-3719 (1997).  RecN is involved in double strand break repair in bacteria	11	225	32	290	28	154	166	2260	418	2748	0.000347190059533779	0.999998877031569			3	3	4	22	24	53	43	65	57	112	16	58	49	71	580		32	25	16	41	23	84	17	12	3	20
DDB_G0278935	rad18	max mid devel	too little data	growth&devel	2.15	6.97		DNA replication/post-replication repair	RAD18 orthologue, a E3 ubiquitin ligase required to ubiquitylate PCNA and allow bypassing of DNA lesions that are subsequently repaired by "postreplication repair"; the corresponding E2 (rad6) is not rblA-regulated	8	48	10	72	8	13	41	151	77	274	0.00930918073629183	0.999998877031569			5	4	5	3	11	25	23	45	25	21	3	2	5	10	187		6	3	2	8	3	17	5	6	1	7
DDB_G0275809	msh2	max mid devel	max late G2	growth&devel	2.72	9.14		DNA replication/post-replication repair	mutS homologue, misincorporated base recognition in methyl-activated repair immediately after replication, forms dimer with Msh3 or Msh6	69	675	200	932	31	189	394	1433	964	2959	0.00101236948757402	0.999998877031569			10	16	13	43	80	179	171	232	111	179	20	39	27	79	1199		154	106	67	243	130	557	98	75	42	82
DDB_G0281683	msh3	max mid devel	max late G2	growth&devel	3.29	5.47		DNA replication/post-replication repair	mutS homologue, misincorporated base recognition in methyl-activated repair immediately after replication, forms dimer with Msh2, has conserved PIP domain	130	1436	551	1969	49	225	353	1135	1840	4008	0.0137303162405214	0.979621085641725			18	11	10	46	133	323	207	303	104	202	6	36	10	18	1427		265	169	124	376	201	779	174	148	60	129
DDB_G0268614	msh6	max mid devel	max late G2	growth&devel	3.21	6.48		DNA replication/post-replication repair	mutS homologue, misincorporated base recognition in methyl-activated repair immediately after replication, forms dimer with Msh2, has conserved PIP domain	128	864	453	1379	78	249	692	1373	1319	3897	0.00445018058461314	0.979621085641725			44	49	47	112	200	552	283	440	146	357	24	71	37	90	2452		372	274	191	452	232	958	197	197	101	170
DDB_G0270764	myh	max mid devel	max late G2	growth&devel	2.37	7.78		DNA replication/post-replication repair	mutH homologue, glycosylase that removes misincorporated base in methyl-activated repair which occurs immediately after replication fork passage	11	169	19	257	8	68	87	433	256	796	0.004737621973903	0.999998877031569			3	0	2	11	16	55	30	50	30	62	1	10	6	7	283		18	13	16	56	29	95	14	14	10	20
DDB_G0279923	apnA	max mid devel	max late G2	growth&devel	3.64	16.91		DNA replication/post-replication repair	nuclease specific for abasic nucleotides, probable role in methyl-activated repair which occurs immediately after replication fork passage; has a canonical PIP domain that is conserved in the D. purpureum gene	27	254	91	519	28	130	472	2927	439	4009	0.0000100401666996739	0.829396312975294			2	2	1	17	60	116	91	148	67	148	8	47	35	68	810		41	20	21	57	43	129	37	29	11	16
DDB_G0289513	rfa1	"repair"	max late G2	growth&devel	4.47	11.08		double-strand break repair (HR)	DNA single-strand binding protein, coats DNA in unreplicated gap in first step of lesion repair by homologous recombination; note that putative error-prone polymerase polZ is not RblA-regulated and presumably plays no role in replication-coupled processes	198	1749	936	3980	87	424	1311	4061	2458	10288	0.000211699970407891	0.459521733171895			279	248	37	100	191	487	384	561	216	560	17	75	31	73	3259		377	312	271	616	342	1275	340	314	132	256
DDB_G0273139	rad51	growth phase + some throughout	min late G2	growth&devel	4.12	3.18		double-strand break repair (HR)	rad51 homologue; forms nucleoprotein filament together with ssDNA and Rfa1	128	669	595	1325	148	240	495	968	1185	3383	0.182597346001926	0.492536516855637			5605	1523	231	470	593	1394	905	829	373	1466	169	479	313	925	15275		162	115	52	57	13	117	83	79	34	85
DDB_G0269406	rad52	growth phase + some throughout	max late G2	growth&devel	4.68	4.67		double-strand break repair (HR)	rad52 orthologue, single-strand end binder which aids in duplex invasion during homologous recombination	27	168	164	337	14	40	84	180	249	765	0.0366847781640788	0.343936089492607			115	102	8	22	11	33	26	25	6	33	2	10	3	11	407		19	23	18	29	14	40	16	9	4	10
DDB_G0279167	topbp1	later development	max late G2	growth only	2.46	0.72		DNA-replication/replication-coupled repair?	putative orthologue of the vertebrate topoisomerase binding protein TopBP1, which recruits 9-1-1 to DNA lesions; weakly RblA regulated, included here for comparison	21	363	65	369	270	873	225	698	1527	1357	1	0.999998877031569			237	107	154	450	260	528	364	489	361	622	211	494	286	609	5172		86	41	20	60	33	141	46	34	16	48
DDB_G0274525	rad9	"repair"	max S-early G2	growth&devel	3.09	4.25		DNA replication/replication-coupled repair	9-1-1-checkpoint clamp complex subunit, loaded onto DNA at sites of DNA damage, activates ATR-mediated phosphorylation for replication fork stabilization and repair; function said to be replication-coupled (Lupardus et al, Genes Dev 16, 2327-2332 (2002)); rad1 and rad17 are also involved in this pathway but show much lower induction factors, suggesting that they also have other roles	46	283	159	425	27	75	151	294	431	1029	0.0488528632966839	0.999998877031569			337	106	11	28	29	93	62	78	30	77	5	29	10	17	912		39	13	15	28	10	69	29	20	10	24
DDB_G0285353	hus1	"repair"	max late G2	growth&devel	2.97	19.01		DNA replication/replication-coupled repair	9-1-1-checkpoint clamp complex subunit, loaded onto DNA at sites of DNA damage, activates ATR-mediated phosphorylation for replication fork stabilization and repair; function said to be replication-coupled (Lupardus et al, Genes Dev 16, 2327-2332 (2002)); rad1 and rad17 are also involved in this pathway but show much lower induction factors, suggesting that they also have other roles	4	155	16	182	1	36	34	219	196	451	0.00559607030089406	0.999998877031569			82	13	5	11	23	30	30	32	10	31	2	6	4	8	287		10	5	2	20	4	31	6	2	0	9
DDB_G0269378	rad1	max growth + some later	min late G2	growth only	2.31	0.78		DNA-replication/replication-coupled repair?	9-1-1-checkpoint clamp complex subunit, loaded onto DNA at sites of DNA damage, activates ATR-mediated phosphorylation for replication fork stabilization and repair; function said to be replication-coupled (Lupardus et al, Genes Dev 16, 2327-2332(2002)); rad1 and rad17 are also involved in this pathway but show much lower induction factors, suggesting that they also have other roles; included here for comparison	26	209	57	257	26	70	21	72	331	407	1	0.999998877031569			51	93	8	26	15	54	38	46	8	26	6	15	14	27	427		26	28	24	19	6	27	18	17	10	19
DDB_G0292504	rad17	terminal differentiation	flat	growth only	2.3	1.19		DNA-replication/replication-coupled repair?	interacts with Rfc2-5 to load 9-1-1-complex; not substantially regulated by RblA; included here for comparison	12	124	27	154	42	69	48	113	247	342	1	0.999998877031569			22	21	4	9	19	17	19	30	30	46	10	33	30	37	327		33	17	12	22	9	47	10	10	3	29
DDB_G0291380	atr1	max mid devel	max late G2	growth only	2.23	0.66		DNA-replication/replication-coupled repair?	ATR kinase, activated by 9-1-1 complex; interacts with Rfc2-5 to load 9-1-1-complex; not substantially regulated by RblA; included here for comparison	55	668	148	669	71	258	57	182	1052	1056	1	0.999998877031569			101	95	81	184	232	445	208	288	231	345	45	124	46	96	2521		86	41	20	60	33	141	46	34	16	48
DDB_G0293656	fhkA	"repair"	max late G2	growth&devel	10.07	10.93		DNA replication/replication-coupled repair	one of 5 Dictyostelium discoideum "fhkX" genes, the only one which is RblA-repressed.  Putative S/T kinase with phosphopeptide-interaction domain in Rad53/Chk2 family. Predicted to interact with phosphorylated Rad9	1	42	15	143	2	30	31	260	75	449	0.00233971644892731	0.184424782611907			8	13	6	9	21	32	43	75	50	48	3	14	6	6	334		6	6	3	12	15	27	3	3	1	2
DDB_G0286539	DDB_G0286539 (53BP1)	"repair"	max late G2	growth&devel	2.99	4.22		DNA-replication-replication-coupled repair?	BRCT domain-containing protein, mutual best BLAST hit with human p53-binding protein.  The homology is mainly in the BRCT domain, but other small islands of homology are recognized by Blast2Sequences over most of the length . p53-binding protein, aka 53BP1, is thought to be a mediator between sensors of double-strand breaks and effectors of repair processes, cell cycle arrest, and apoptosis, see Fitzgerald et al, Biochemical Society Transactions 37, 897-904 (2009). Strongly cell-cycle regulated	19	248	70	326	19	87	87	442	373	925	0.0466543149045054	0.999998877031569			35	11	3	7	23	62	42	67	31	44	1	5	6	11	348		51	39	28	101	52	215	30	45	18	26
DDB_G0287899	rif1	max mid devel	max late G2	growth&devel	7.96	17.21		DNA-replication-replication-coupled repair?	Orthologue of yeast telomere associated protein Rif1; in humans Rif1 does not accumulate at telomeres, thought to accumulate at double-strand breaks downstream of 53BP1 and inhibit DNA replication after DNA damage.  In Dictyostelium discoideum developmental expression pattern differs from most DNA repair genes, resembles normal S-phase genes	11	121	68	599	11	92	266	1282	235	2215	0.0000367436172579966	0.0679153676322319			7	2	2	9	60	117	101	196	122	144	12	21	9	18	820		19	8	13	52	34	110	18	11	5	15
DDB_G0278203	xrcc4	"repair"	max late G2	growth&devel	4.76	8.02		DNA-replication/replication-coupled repair?	associates with Lig4 and PNKP in "X4L4" ligation complex involved in DNA repair, thought to be involved in NHEJ but overexpression factor higher than most NHEJ components	2	18	3	69	0	6	11	41	26	124	0.0152468074752766	0.642173226353793			6	3	0	3	1	8	10	6	3	6	0	0	0	0	46		3	0	0	10	3	9	0	2	0	2
DDB_G0292760	lig4	"repair"	max late G2	growth&devel	3.75	7.44		DNA-replication/replication-coupled repair?	associates with Xrcc4 and PNKP in "X4L4" ligation complex involved in DNA repair, thought to be involved in NHEJ but overexpression factor higher than most NHEJ components	13	97	42	214	7	29	61	234	146	551	0.00392132833661926	0.838655565457967			53	19	3	17	17	36	27	50	34	48	3	4	6	7	324		27	16	7	30	20	67	22	14	5	11
DDB_G0281229	pnkp	mostly growth phase	max late G2	growth only	2.55	1.65	DNA-replication-replication-coupled repair?	associates with Lig4 and Xrcc4 in "X4L4" ligation complex involved in DNA repair, thought to be involved in NHEJ but overexpression factor higher than most NHEJ components 	associates with Lig4 and Xrcc4 in "X4L4" ligation complex involved in DNA repair, thought to be involved in NHEJ 	185	1691	541	2047	45	148	92	250	2069	2930	0.946014613520646	0.999998877031569			1072	465	51	118	61	170	87	108	20	76	24	97	30	99	2478		189	187	105	245	117	470	177	123	48	116
DDB_G0293866	DDB_G0293866 (aprataxin)	"repair"	max late G2	growth&devel	2.32	4.92		DNA-replication/replication-coupled repair?	aprataxin-like protein which may resolve abortive ligation intermediates, possible partner of the X4L4 complex	5	110	9	159	12	58	73	287	185	528	0.0310455136100562	0.999998877031569			23	20	11	10	11	45	44	32	18	27	6	9	7	5	268		14	12	5	16	5	26	8	4	3	15
DDB_G0286069	ku70	"repair"	flat	not substantially RblA regulated	1.37	0.61		NHEJ 	not strongly RblA regulated; involved in non-homologous end joining, like Xrcc4, Lig4, and PNKP but differently regulated	46	599	76	376	24	130	20	67	799	539	1	0.999998877031569			71	32	19	55	27	31	46	61	24	36	6	16	19	27	470		69	44	18	30	14	72	30	22	11	23
DDB_G0286303	ku80	"repair"	cold-activated?	not substantially RblA regulated	1.1	0.66		NHEJ 	not strongly rblA regulated; involved in non-homologous end joining, like Xrcc4, Lig4, and PNKP but differently regulated	44	559	47	335	16	111	15	58	730	455	1	0.999998877031569			25	39	6	35	15	33	38	47	21	50	3	12	16	17	357		58	62	43	63	35	126	32	23	8	33
DDB_G0284713	polB	near flat	near flat	not substantially RblA regulated	1.82	0.76		NHEJ	not strongly rblA regulated; involved in non-homologous end joining, like Xrcc4, Lig4, and PNKP but differently regulated	4	144	7	144	4	42	4	30	194	185	1	0.999998877031569			7	18	4	5	7	13	6	14	9	17	2	5	5	5	117		14	15	5	12	5	22	3	1	1	13
DDB_G0268512	wrn	later development	near flat	growth&devel	1.97	1.43		NHEJ	involved in non-homologous end joining, like Xrcc4, Lig4, and PNKP	9	120	23	98	48	146	63	295	323	479	1	0.999998877031569			25	28	3	24	42	54	79	50	43	42	47	57	53	113	660		30	16	9	23	5	42	13	9	4	18
DDB_G0278229	DDB_G0278229	"repair"	max late G2	growth&devel	2.21	5.69		DNA replication/replication-coupled repair?	no PARP domain but contains conserved MACRO (high-affinity ADP-ribose binding) domain and three conserved WWE domains common in proteins involved in ubiquitylation and ADP ribosylation. The gene is mildly cell cycle regulated.  Function likely downstream of ADP ribosylation, possibly in replication-coupled repair	65	523	136	641	24	101	165	611	713	1553	0.0104073604443293	0.999998877031569			377	128	14	19	29	61	79	95	65	108	9	18	17	68	1087		110	69	37	94	43	181	58	39	16	59
DDB_G0267682	ercc1	near flat	near flat	not substantially RblA regulated	1.17	0.42		base-excision repair comparison gene	base-excision repair pathway, not substantially RblA-regulated, included here for comparison	32	249	39	151	15	44	6	26	340	222	1	0.999998877031569			7	11	12	30	18	20	13	19	10	12	0	4	5	19	180		42	30	14	24	5	40	5	13	6	14
DDB_G0284419	ercc4/xpf	max mid devel	near flat	growth only	3.76	0.33		base-excision repair comparison gene	base-excision repair pathway, not substantially RblA-regulated, included here for comparison	2	167	14	119	11	85	4	33	265	170	0.938480096471342	0.999998877031569			6	5	7	46	29	42	35	65	24	30	7	22	18	19	355		13	12	16	13	7	43	12	3	7	8
DDB_G0281441	ercc6	"repair"	near flat	growth only	2.3	0.52		base-excision repair comparison gene	base-excision repair pathway, not substantially RblA-regulated, included here for comparison	50	325	115	398	248	670	144	407	1293	1064	1	0.999998877031569			179	111	61	162	190	344	350	412	223	454	122	315	147	335	3405		125	55	49	65	32	127	53	42	9	46
DDB_G0276481	ercc8	later development	near flat	not substantially RblA regulated	1.2	0.61		base-excision repair comparison gene	base-excision repair pathway, not substantially RblA-regulated, included here for comparison	37	229	42	153	75	262	62	143	603	400	1	0.999998877031569			32	12	28	115	24	50	74	83	57	119	30	110	39	124	897		23	31	21	17	18	51	20	18	3	15
DDB_G0278729	repB	later development	near flat	not substantially RblA regulated	1.24	0.7		base-excision repair comparison gene	base-excision repair pathway, not substantially RblA-regulated, included here for comparison	124	1233	166	766	591	1739	474	1415	3687	2821	1	0.999998877031569			158	174	105	384	242	600	539	686	360	869	193	773	316	956	6355		232	213	154	202	97	381	116	139	57	131
DDB_G0292296	rad4/xpc	max mid devel	near flat	not substantially RblA regulated	1.52	1.32		base-excision repair comparison gene	base-excision repair pathway, not substantially RblA-regulated, included here for comparison	48	668	67	570	31	221	53	270	968	960	1	0.999998877031569			45	56	40	93	105	247	170	209	142	230	38	82	43	91	1591		40	54	44	33	21	76	35	20	11	40
DDB_G0283857	lig3	"repair"	near flat	growth	2.05	0.98		base-excision repair comparison gene	base-excision repair pathway, not substantially RblA-regulated, included here for comparison	46	372	79	456	22	170	33	108	610	676	1	0.999998877031569			293	160	34	85	78	145	122	162	61	143	11	56	28	63	1441		79	41	37	51	32	142	43	36	24	47
DDB_G0275653	xrcc1	"repair"	near flat	not substantially RblA regulated	1.74	0.6		base-excision repair comparison gene	base-excision repair pathway, not substantially RblA-regulated, included here for comparison	24	471	42	428	19	112	14	71	626	555	1	0.999998877031569			89	64	5	21	22	46	57	75	24	70	8	26	10	24	541		55	45	27	45	20	94	38	15	9	25
DDB_G0284071	rnrA	max mid devel	max late G2	growth&devel	5.14	13.04		DNA replication/provision of nucleotides	ribonucleotide reductase large subunit	947	5811	5599	14100	962	3141	17903	32028	10861	69630	0.0000632090085624062	0.245790087591803			196	156	142	365	886	2956	2189	2394	921	3431	218	1137	307	1290	16588		1681	842	512	1843	846	3402	1253	1185	464	896
DDB_G0272616	rnrB-1	max mid devel	max late G2	growth&devel	3.2	8.73		DNA replication/provision of nucleotides	ribonucleotide reductase small subunit	53	1250	211	1746	95	767	1182	5226	2165	8365	0.00155493823943873	0.999998877031569			13	34	12	42	293	627	678	998	632	998	165	458	204	549	5703		125	62	37	279	166	700	145	122	49	125
DDB_G0288019	DDB_G0288019 (dCMP deaminase)	terminal differentiation	max late G2	growth&devel	2.18	4.59		DNA replication/provision of nucleotides	CMP/dCMP deaminase	50	494	142	448	81	86	342	559	711	1491	0.0471624336183038	0.999998877031569			12	33	5	11	16	43	62	69	21	51	10	45	174	412	964		93	48	34	81	41	205	53	19	13	47
DDB_G0280045	thyA	"repair"	max late G2	devel only	1.22	6.18	DNA replication/provision of nucleotides	DNA replication/provision of nucleotides	thymidylate synthase	649	6835	920	3935	180	605	1415	3669	8269	9939	0.00517417385933876	0.999998877031569			125	49	10	16	92	270	590	715	380	914	38	115	53	152	3519		1708	276	172	1996	1062	4040	1577	1431	605	1076
DDB_G0274161	DDB_G0274161	late development	flat	devel only	0.84	9.01		DNA replication/provision of nucleotides?	similar to NmrA-like family proteins of vertebrates. The human orthologue is NMRAL1 or HSCARG, and is said to act as a redox sensor, monitoring the ratio of NAD to NADPH (Zheng et al, PNAS 104, 8809-8814 (2007)). 	1	13	1	5	1	7	13	48	22	67	0.0239617072534825	0.999998877031569			1	2	1	3	0	1	0	5	4	1	5	13	4	6	46		6	3	2	0	0	5	1	0	2	3
DDB_G0274269	DDB_G0274269 (HAT1)	mid + late devel	max late G2	growth&devel	7.2	24.63		Chromatin replication/histone biosynthesis	highly similar to human HAT1, subunit of HAT1/HAT2 histone acetyl transferase complex; the Dictyostelium orthologue of HAT2 is RbbD.  Hat1 is a "type B" histone acetyl transferase;  originally thought to acetylate the tails of newly-synthesized H3 and H4 prior to their assembly into chromatin.  In yeast HAT1 is non-essential for growth but has a role in recombinational repair of DNA double-strand breaks, possibly by contributing to the assembly of newly-synthesized DNA into chromatin; see Qin and Parthun, Molecular and Cellular Biology 22, 8353-8365 (2002).	1	153	11	356	5	78	138	2254	237	2759	0.00000237422386587444	0.555048345560071			1	1	4	9	23	48	34	60	69	48	22	23	53	52	447		9	3	5	17	19	56	16	5	3	6
DDB_G0282529	rbbD	max mid devel	max late G2	growth&devel	2.69	25.66		Chromatin replication/histone biosynthesis	Histone binding protein, similar to mammalian RBBP4/7 aka  RbAp48  or Caf1p55 of Drosophila, Hat2 of yeast; participates in several complexes including Hat1/Hat2 histone acetylation complex,  CAF-1 chromatin assemby factor complex (possibly with DDB_G0287815 and DDB_G0269800), NURF complex.  Dicty has no recognizable homologues of NURF complex component BPTF but does have SMARAC1 (called isw).  May also be a member of LINC complex which regulates expression of G2/M genes, see Schmit et al, Cell Cycle 6, 1903-1913 (2007).  Expression level much higher than LINC complex members, thus main function may be with Hat complex	156	1780	525	2076	28	224	973	5017	2188	8591	0.00000246285356288029	0.999998877031569			17	11	10	104	84	261	154	252	72	208	8	41	8	28	1258		293	215	146	383	181	775	187	180	72	162
DDB_G0284195	DDB_G0284195 (COBRA1)	"repair"	max late G2	growth&devel	3.63	20.97		Chromatin replication/histone biosynthesis	Highly similar in N-terminal half to cofactor of BRAC1, also known as COBRA1. In mammals COBRA1 participates in a four-protein complex called NELF (Negative ELongation Factor) which negatively regulates steroid-hormone induced gene expresssion.  Cobra1 expression is correlated with good prognosis in breast cancer.  Other components of the complex are found in Dictyostelium; middling homologies for  NelfA and RDBP, strong homology for NELFC/C, aka THI; not one of these is significantly RblA regulated.    Recent work shows that NELF participates in 3' end processing of histone mRNAs in association with the SLBP, see Narita et al, Mol.Cell 26, 349-365 (2007) 	40	295	145	570	16	87	517	1160	438	2392	0.0000100401666996739	0.819164776245306			24	23	8	28	55	152	118	138	50	128	8	33	11	48	824		88	68	42	95	40	157	45	46	24	39
DDB_G0283113	eriA	mid + late devel	max late G2	growth&devel	3.18	7.09		Chromatin replication/histone biosynthesis	multifunctional RNA exonuclease; in mammalian cells EriA, under the pseudonym 3'hExo, stably associates with histone pre-mRNAs and participates in a complex that determines the site of their cleavage (see Yang et al, Mol Cell Biol 29, 4045-4056 (2009)).	4	217	19	232	3	67	34	268	291	553	0.0583678284899483	0.999998877031569			0	2	1	6	6	3	14	18	12	11	9	7	8	16	113		14	3	1	25	12	45	16	8	5	6
DDB_G0287815	DDB_G0287815 (CAF1a)	max mid devel	max late G2	growth&devel	2.78	3.91		DNA replication/chromatin assembly	chromatin assembly factor CAF1, subunit A (subunit C is rbbD, listed under "histone biosynthesis")	58	771	195	955	26	203	150	574	1058	1874	0.147669590475131	0.999998877031569			4	1	1	6	18	59	93	131	66	117	5	9	5	18	533		68	51	34	141	75	281	74	56	24	39
DDB_G0269800	DDB_G0269800 (CAF1b)	max mid devel	max late G2	growth&devel	3.16	8.79		DNA replication/chromatin assembly	chromatin assembly factor CAF1, subunit B (subunit C is rbbD, listed under "histone biosynthesis")	18	261	58	433	11	119	148	653	409	1292	0.00457865655772958	0.999998877031569			2	3	5	23	45	90	100	153	62	120	2	27	12	16	660		57	42	18	80	38	159	24	21	3	18
DDB_G0285513	asf1	mid + late devel	max late G2	growth&devel	3.97	28.33		DNA replication/chromatin assembly	"anti-silencing factor", conserved nucleosome assembly factor and histone chaperone protein	16	208	57	502	14	94	448	2786	332	3793	0.00000110181427088572	0.642173226353793			3	5	1	12	22	46	46	81	24	73	12	30	18	40	413		21	14	10	51	26	98	17	10	4	16
DDB_G0292444	DDB_G0292444 (SAP)	max mid devel	max late G2	growth&devel	6.92	15.7		Chromosome structure	contains SAP DNA-binding domain predicted to be involved in chromosomal organization, see Aravind and Koonin, Trends in Biochemical Sciences 25, 114-114 (2000).	1	59	6	239	2	29	27	686	91	958	0.0000219542077360377	0.172771371905485			0	0	0	1	3	13	21	18	13	14	4	8	3	6	104		4	1	2	6	5	21	7	1	2	3
DDB_G0277405	DDB_G0277405 (SAP)	"repair"	max late G2	growth&devel	4.71	14.01		Chromosome structure	contains SAP DNA-binding domain predicted to be involved in chromosomal organization, see Aravind and Koonin, Trends in Biochemical Sciences 25, 114-114 (2000).	471	6387	2694	13539	64	401	1263	4428	7323	21924	0.0000732758777499943	0.404199525262592			54	78	12	42	99	390	241	286	104	182	7	35	13	32	1575		685	535	369	939	511	1916	486	468	216	347
																		0	0
DDB_G0291752	smc1	"repair"	max late G2	growth&devel	4.41	16.93		DNA replication/sister-chromatid cohesion	cohesin complex, SMC subunit	53	698	241	1597	60	307	1332	4834	1118	8004	0.0000163040123578152	0.492536516855637			120	58	9	25	140	400	245	455	165	340	14	37	20	37	2065		191	156	104	310	164	631	148	113	31	83
DDB_G0276101	smc3	"repair"	max late G2	growth&devel	3.39	14.48		DNA replication/sister-chromatid cohesion	cohesin complex, SMC subunit	71	931	294	1661	67	494	1352	5895	1563	9202	0.0000631953299902737	0.78827390645666			70	40	17	46	157	505	245	494	274	482	10	58	16	38	2452		190	163	117	357	183	715	171	122	45	103
DDB_G0283681	DDB_G0283681 (STAG)	"repair"	max late G2	growth&devel	5	10.86		DNA replication/sister-chromatid cohesion	similar to STAG (STromal AntiGen) proteins, which are subunits of a protein complex required for sister chromatid cohesion in eukaryotes (Dictybase)	43	392	184	1155	61	302	926	2686	798	4951	0.000269201843913651	0.340174534421176			40	31	5	8	117	327	202	343	222	404	7	38	10	23	1777		142	86	47	193	117	419	111	79	32	70
DDB_G0283099	DDB_G0283099 (Nipped-B)	"repair"	max late G2	growth&devel	3.11	8.7		DNA replication/sister-chromatid cohesion	orthologue of nippedB/Scc2, which loads cohesin onto nascent DNA and is required for sister chromatid cohesion, see Dorsett, Chromosome Research 17, 185-200 (2009)	33	573	102	955	25	237	320	1496	868	2873	0.00249005759134834	0.999998877031569			59	63	33	90	148	260	189	362	233	290	34	68	50	101	1980		129	133	77	176	90	417	95	70	22	68
DDB_G0276977	rad21	"repair"	max late G2	growth&devel	3.62	19.86		DNA replication/sister-chromatid cohesion	orthologue of RAD21/SCC1, component of the cohesin complex	91	759	402	1216	53	214	1447	3579	1117	6644	0.00000539282314761753	0.747893683395964			157	153	32	60	189	556	342	498	164	435	27	91	29	80	2813		163	129	94	229	127	523	162	125	53	81
DDB_G0275929	pds5	"repair"	max late G2	growth&devel	4.18	5.47		DNA replication/sister-chromatid cohesion	premature dissociation of sisters, interacts with the chromosomal cohesion complex and modulates the cohesion of chromosomes and kinetochores (see Dorsett, Chromosoma 116, 1-13 (2007)).	42	636	176	1416	72	375	494	2060	1125	4146	0.0122308993787186	0.560874957542999			26	48	10	26	97	217	192	353	187	394	38	90	21	42	1741		145	81	52	238	132	581	131	94	38	137
DDB_G0279959	eco1	"repair"	max late G2	growth&devel	6.32	5.22		DNA replication/sister-chromatid cohesion	Establishment of COhesion homologue 2, an acetyltransferase required for the establishment of chromosome cohesion in S-phase (Kim et al, BBRC 372, 298-304 (2008))	343	3794	2399	11741	169	875	1174	4082	5181	19396	0.0178181990019936	0.13215712714221			292	152	146	479	85	245	486	907	308	796	22	145	20	87	4170		403	134	107	255	149	583	209	153	69	186
DDB_G0270814	nek2	max mid devel	max late G2	growth&devel	3.01	7.82		Centrosome	homologous to human Nek2 kinase; a NIMA-related kinase with centrosome nucleation function, see Graf et al, In Rev Cytol 241, 155-202 (2004).	23	536	68	897	5	80	57	465	644	1487	0.00959268648185904	0.999998877031569			1	1	2	1	18	33	40	83	50	40	1	7	1	1	279		38	28	20	91	58	201	36	31	6	26
DDB_G0285849	spc97	max mid devel	max late G2	growth&devel	2.36	4.85		Centrosome	centrosomal protein involved in microtubule nucleation, see Graf et al, In Rev Cytol 241, 155-202 (2004).	68	493	172	589	39	151	227	784	751	1772	0.0228123493254437	0.999998877031569			6	10	15	32	49	110	81	135	71	111	11	16	20	39	706		93	49	29	95	33	142	44	52	15	34
DDB_G0283909	spc98	max mid devel	max late G2	growth&devel	3.03	19.89		Centrosome	centrosomal protein involved in microtubule nucleation, see Graf et al, In Rev Cytol 241, 155-202 (2004).	17	157	57	233	7	67	231	646	248	1167	0.0000692188567645392	0.999998877031569			5	2	8	14	38	80	97	109	23	57	2	15	5	17	472		53	29	12	82	30	124	20	30	8	31
DDB_G0284031	spc24	max mid devel	too little data	growth&devel	6.999497496061251	8.83936285895697		Centrosome	putative centrosomal protein	0	7	2	52	0	5	3	72	12	129	0.00456695685423563	0.106670671130269			0	0	0	2	2	6	4	11	4	4	0	5	1	2	41		2	2	1	1	3	7	2	1	0	3
DDB_G0268212	DDB_G0268212	growth phase + some late	flat	growth&devel	4.32	16.96		Centrosome	putative DEAD-box helicase; centrosomal protein of unknown function (Reinders et al, J Proteome Research 5, 589-598 (2006))	10	156	39	379	4	31	76	617	201	1111	0.0000316900381607414	0.566019254096926			7	14	2	2	2	1	2	4	1	0	3	0	3	7	48		16	8	4	9	4	16	5	2	3	4
DDB_G0275495	DDB_G0275495 (CP91)	max mid devel	max late G2	growth&devel	3.16	7.98		Centrosome	CP91, centrosomal core protein, see Schulz et al, Cell Motil. Cytoskeleton 66, 915-928 (2009)	58	504	217	736	36	130	379	916	728	2248	0.00152946022693424	0.999998877031569			7	5	18	47	28	80	49	118	24	28	2	10	10	18	444		80	65	35	125	63	254	62	64	21	48
DDB_G0283111	DDB_G0283111 (CP75)	"repair"	max late G2	growth&devel	2.84	10.58		Centrosome	CP75, centrosomal core protein, see Schulz et al, Cell Motil. Cytoskeleton 66, 915-928 (2009)	24	188	55	326	10	49	145	443	271	969	0.000501872214779555	0.999998877031569			3	7	1	2	2	15	26	47	17	33	3	2	6	11	175		21	19	9	29	17	52	16	19	6	10
DDB_G0285313	DDB_G0285313	max mid devel	max late G2	growth&devel	2.92	10.69		Centrosome	Cep192, centrosomal interactor with polo like kinase, see Schulz et al, Cell Motil. Cytoskeleton 66, 915-928 (2009); 12-fold enriched in mitotic centrosomes; regulates recruitment of pericentrosomal matrix in centrosome duplication, see  Zhu et al, Current Biology 18, 136-141 (2008)	54	455	151	728	27	154	429	1163	690	2471	0.00046732840276533	0.999998877031569			12	5	13	25	75	137	132	192	95	143	3	14	12	27	885		71	61	36	89	52	190	45	31	7	45
DDB_G0285357	DDB_G0285357	"repair"	max late G2	devel only	1.49	8.6	centrosome	Putative peptidyl-prolyl isomerase; centrosomal protein of unknown function (Reinders et al, J Proteome Research 5, 589-98 (2006))		2743	12335	5418	7417	488	1197	5937	1163	16763	19935	0.000626596504949328	0.999998877031569			802	291	82	133	184	365	376	426	192	462	83	203	162	424	4185		2304	1287	760	1311	622	2545	1085	1328	487	976
DDB_G0284499	smc2	max mid devel	max late G2	growth&devel	4.16	24.88		Mitosis/chromosome condensation	condensin complex, SMC subunit	88	1057	390	2223	40	298	1409	5897	1483	9919	0.00000267889915379522	0.560874957542999			8	8	13	36	100	277	194	275	118	229	15	60	31	58	1422		170	146	113	376	225	918	153	149	65	98
DDB_G0286403	smc4	max mid devel	max late G2	growth&devel	5.02	14.86		Mitosis/chromosome condensation	condensin complex, SMC subunit	49	732	287	1714	37	239	827	2430	1057	5258	0.0000732758777499943	0.34643996093787			10	9	4	11	72	195	96	181	72	124	5	21	9	17	826		236	119	84	323	153	630	169	117	48	85
DDB_G0293984	ncapD2	max mid devel	max late G2	devel only	1.78	36.02		Mitosis/chromosome condensation	non-SMC condensin I subunit D2	18	442	30	440	9	136	435	4362	605	5267	0.000000577701011488139	0.999998877031569			0	2	4	11	7	21	40	75	48	53	9	13	20	17	320		36	28	20	66	35	158	29	15	8	25
DDB_G0275129	ncapGb	mid + late devel	too little data	growth&devel	7.16	5.01		Mitosis/chromosome condensation	similar to non-SMC condensin I complex, subunit G	2	32	20	86	9	40	55	204	83	365	0.0315607817922011	0.29052920670834			12	7	2	8	16	29	21	27	46	50	13	26	22	44	323		0	3	1	5	2	5	2	2	0	2
DDB_G0281419	ncapGa	max mid devel	max late G2	growth&devel	5.04	15.15		Mitosis/chromosome condensation	similar to non-SMC condensin I complex, subunit G	2	228	15	390	4	49	79	695	283	1179	0.000100457125995213	0.999998877031569			0	0	0	0	8	10	33	48	35	34	0	6	2	0	176		22	7	9	32	17	63	14	7	4	11
DDB_G0292066	ncapH	mid + late devel	max late G2	growth&devel	2.09	2.13		Mitosis/chromosome condensation	similar to non-SMC condensin I subunit H of plants, also known as "barren".	36	302	74	341	36	236	107	414	610	936	0.794284866574293	0.999998877031569			14	13	21	78	82	197	128	162	80	158	27	71	46	101	1178		83	47	28	59	38	156	43	28	11	32
DDB_G0278701	ncapD3	max mid devel	max late G2	growth&devel	4.03	23.75		Mitosis/chromosome condensation	non-SMC Condensin II complex, subunit D3	80	723	389	1307	54	185	1639	4248	1042	7583	0.00000237422386587444	0.579994646127989			3	1	5	19	64	194	93	230	79	144	10	21	8	23	894		161	101	65	246	109	485	102	90	35	78
DDB_G0288819	ncapG2	max mid devel	max late G2	growth&devel	19.71	18.03		Mitosis/chromosome condensation	non-SMC condensin II subunit G2	1	160	40	388	18	128	433	2077	307	2938	0.0000168911305967821	0.230697785786267			0	0	2	4	62	100	119	216	121	172	6	13	2	9	826		36	22	16	59	36	148	36	19	6	18
DDB_G0291894	ncapH2	max mid devel	max late G2	growth&devel	4.67	20.15		Mitosis/chromosome condensation	non-SMC condensin II complex subunit H2	13	259	58	665	15	80	297	2175	367	3195	0.00000388919961623071	0.457383486394951			1	1	3	4	30	89	79	101	54	95	8	31	15	34	545		40	11	15	69	40	129	22	11	8	16
DDB_G0288873	pich	mid + late devel	max late G2	growth&devel	3.43	7.19		Mitosis/chromosome decatenation	Helicase originally thought to be intrakinetochore tension sensor (Baumann et al, Cell 128, 101-114 (2007)), role now seems more likely to be in decatenating sister chromatids in preparation for mitosis	73	1169	289	1891	50	223	437	1679	1515	4296	0.00244829886990909	0.936663356381459			28	20	15	24	33	126	130	243	112	213	94	208	60	112	1418		161	170	98	290	160	524	110	122	34	65
DDB_G0290919	smc5	max mid devel	max late G2	growth&devel	3.17	13.32		Mitosis/cohesin removal	role in separase-independent removal of cohesin, see Outwin et al, Molecular and Cellular Biology 29, 4363-4375 (2009); its widely known role in homologous recombination may reflect this function	78	629	259	1026	58	327	1159	3000	1092	5444	0.000110555079740676	0.999998877031569			14	6	11	42	109	360	219	400	146	291	17	60	17	45	1737		206	174	98	221	93	448	124	83	47	101
DDB_G0288993	smc6	max mid devel	max late G2	growth&devel	2.16	5.39		Mitosis/cohesin removal	role in separase-independent removal of cohesin, see Outwin et al, Molecular and Cellular Biology 29, 4363-4375 (2009); its widely known role in homologous recombination may reflect this function	151	1560	341	1729	118	681	908	2855	2510	5833	0.0194763180183512	0.999998877031569			83	40	238	679	313	843	512	739	286	673	56	208	102	224	4996		252	209	106	217	101	507	171	112	59	144
DDB_G0287689	tubA	growth and early development	flat	not substantially RblA regulated	1.51	1.26		Mitosis/spindle	not substantially RblA-regulated; included here for comparison	5322	36679	9565	25148	951	2530	1591	2900	45482	39204	1	0.999998877031569			3908	4388	3387	8023	1277	3585	1314	1678	214	978	78	435	124	616	30005		7051	5120	3333	3985	1940	7995	3063	3035	1183	3462
DDB_G0281889	tubA2	max aggregation	max late G2	devel only	1.92	3.73		Mitosis/spindle	second alpha tubulin gene, regulation suggests possible mitotic role	81	1333	165	1304	200	1033	780	4846	2647	7095	0.0638481607540574	0.999998877031569			40	48	214	921	136	279	168	236	200	360	75	137	64	164	3042		150	216	150	199	141	464	108	89	45	111
DDB_G0269196	tubB	growth and early development	flat	not substantially RblA regulated	1.23	1.42		Mitosis/spindle	not substantially RblA-regulated; included here for comparison	4227	19029	6232	10598	827	1356	1474	1948	25439	20252	1	0.999998877031569			1381	2072	2102	4791	359	820	871	894	190	710	67	424	106	512	15299		4947	2762	1819	2655	1306	5216	2200	2180	955	2562
DDB_G0271738	tubC	max mid devel	max late G2	growth&devel	4.53	17.43		Mitosis/spindle	gamma tubulin, required for microtubule nucleation at centrosome	78	322	351	781	21	72	559	821	493	2512	0.0000171715757798709	0.456246821830309			22	8	14	43	69	219	165	176	35	137	5	30	4	32	959		161	91	54	181	72	332	91	104	38	67
DDB_G0268516	tbcD	"repair"	flat	growth only	2.04	2.78		Mitosis/spindle	tubulin folding cofactor D, centrosomal protein required for anchoring of the gamma tubulin ring complex, see Cunningham and Kahn, J Biol Chem 283, 7155-7165 (2008)	11	110	26	105	3	64	15	54	188	200	1	0.999998877031569			12	23	12	24	35	39	42	47	31	55	16	20	9	18	383		40	27	16	28	16	51	11	11	5	19
DDB_G0271286	clasp	max mid devel	max late G2	growth&devel	4.24	4.48		Mitosis/spindle	plus-end tracking protein required for spindle assembly (Pereira et al, Mol Biol Cell 17, 4526-4542 (2006))	12	219	47	523	7	57	94	430	295	1094	0.0017384274087583	0.569824541284444			1	0	0	3	6	24	31	48	19	40	2	2	2	1	179		23	21	19	39	31	112	25	18	9	21
DDB_G0283607	eb1	max aggregation	max late G2	growth&devel	2.33	6.44		Mitosis/spindle	microtubule end binding protein required for spindle assembly (Rehberg and Graf, Mol Biol Cell 13, 2301-2310 (2002))	159	1161	391	1396	47	115	291	1015	1482	3093	0.00457865655772958	0.999998877031569			36	19	42	114	12	29	36	43	15	31	6	25	10	38	456		175	81	51	257	108	498	99	99	48	74
DDB_G0267396	kif2	max mid devel	max late G2	growth&devel	2.41	5.16		Mitosis/spindle	putative mitotic kinesin, see Kollmar and Glöckner, BMC Genomics 4, 47 (2003)	34	458	89	550	18	133	130	562	643	1331	0.0333471916168812	0.999998877031569			0	3	3	13	23	50	101	157	97	190	17	56	18	59	787		63	48	29	146	80	279	71	51	22	36
DDB_G0285101	kif4	max mid devel	max late G2	growth&devel	3.04	6.37		Mitosis/spindle	putative mitotic kinesin, see Kollmar and Glöckner, BMC Genomics 4, 47 (2003)	38	526	111	878	28	289	259	1382	881	2630	0.0141464536625646	0.999998877031569			1	2	2	6	35	138	152	270	192	277	7	19	2	6	1109		120	62	50	195	102	402	82	68	26	61
DDB_G0267404	kif6	max mid devel	max late G2	growth&devel	2.37	7.03		Mitosis/spindle	putative mitotic kinesin, see Kollmar and Glöckner, BMC Genomics 4, 47 (2003)	110	826	240	1099	52	188	495	1146	1176	2980	0.00316669491260838	0.999998877031569			7	12	31	78	65	185	118	191	57	125	9	39	24	58	999		191	80	69	228	90	389	93	71	41	79
DDB_G0293198	kif10	max mid devel	max late G2	growth&devel	5.44	8.92		Mitosis/spindle?	possible mitotic kinesin, see Nag et al, BMC Cell Biology 9, 21 (2008)	14	214	90	536	13	72	154	584	313	1364	0.00122155025203709	0.303121230178081			0	1	1	2	35	80	78	132	41	83	3	10	1	6	473		29	16	15	59	32	126	20	24	7	12
DDB_G0291039	kif11	mid + late devel	max late G2	growth&devel	2.61	3.83		Mitosis/spindle	putative mitotic kinesin, see Kollmar and Glöckner, BMC Genomics 4, 47 (2003)	23	250	62	339	10	58	52	194	341	647	0.138076780108479	0.999998877031569			23	11	5	15	42	94	81	99	35	38	11	55	13	57	579		51	54	51	55	31	126	27	20	11	23
DDB_G0268258	kif12	max mid devel	max late G2	growth&devel	3.05	2.24		Mitosis/spindle	putative mitotic kinesin, see Kollmar and Glöckner, BMC Genomics 4, 47 (2003)	57	1010	215	1351	57	330	168	685	1454	2419	0.648133247240981	0.999998877031569			1	0	2	9	30	95	101	273	123	203	10	14	7	16	884		144	73	44	228	109	460	99	70	30	65
DDB_G0288361	kif13	max mid devel	max late G2	growth&devel	4.68	15.73		Mitosis/spindle	putative mitotic kinesin, see Kollmar and Glöckner, BMC Genomics 4, 47 (2003)	50	781	303	1522	40	231	929	2780	1102	5534	0.0000374287026278935	0.482771354237405			6	3	3	6	44	167	157	317	127	214	8	19	4	11	1086		158	82	71	275	136	595	140	122	55	71
DDB_G0283771	DDB_G0283771	max mid devel	max late G2	growth&devel	3.78	7.14		Mitosis/spindle?	weakly similar to kinesin family proteins; regulation suggests cell cycle role	157	1195	621	2327	106	594	1084	3285	2052	7317	0.00394852166642903	0.677517898017611			35	45	16	40	208	517	390	555	247	568	16	66	11	47	2761		254	167	128	393	194	751	214	185	75	168
DDB_G0293620	DDB_G0293620 (Cenp68)	max mid devel	max late G2	growth&devel	5.68	42.5		Mitosis/centromere	Cenp68, localizes to centromeres (Schulz et al, Cell Motil Cytoskeleton 66, 915-928 (2009))	6	123	26	438	2	26	108	1085	157	1657	0.000000546644809029963	0.224309440567317			0	0	0	0	9	25	24	32	21	22	2	1	0	2	138		17	11	7	36	20	96	10	19	5	16
DDB_G0284013	spc25	max mid devel	max late G2	growth&devel	5.99	35.13		Mitosis/centromere	conserved kinetochore protein, forms heterotrimer with Ndc80 and Nuf2	2	32	9	121	0	15	53	357	49	540	0.00000500456240002335	0.314619138937411			0	0	0	2	2	13	24	20	10	9	1	2	1	0	84		3	6	7	8	4	9	0	2	0	2
DDB_G0279553	nuf2	max mid devel	max late G2	growth&devel	8.57	16.88		Mitosis/centromere	conserved kinetochore protein, forms heterotrimer with Spc25, and Ndc80	4	105	43	388	4	53	100	638	166	1169	0.000125495632307047	0.0976024148708387			11	0	18	32	14	54	44	64	23	33	2	5	3	4	307		20	16	8	39	10	73	10	17	7	23
DDB_G0284003	ndc80	max mid devel	max late G2	growth&devel	5.16	38.36		Mitosis/centromere	conserved kinetochore protein, forms heterotrimer with Spc25 and Nuf2	16	119	61	391	5	35	282	958	175	1692	0.000000881695869915618	0.375394319022356			1	0	2	2	8	29	17	41	20	20	1	3	1	6	151		33	21	15	45	22	78	16	12	6	10
DDB_G0286465	DDB_G0286465 (Haspin)	max mid devel	max late G2	growth&devel	4.24	14.05		Mitosis/centromere	weakly similar (but mutual best BLAST hit) to Haspin, which phosphorylates centromeric histone H3 on Threonine3 in prophase; this is required to maintain centromeric cohesion in early mitosis, see Dai et al, Genes and Development 19, 472-488 (2005)	27	459	126	961	14	104	256	1367	604	2710	0.0000744287663486653	0.579994646127989			4	0	0	2	18	45	41	84	29	38	4	4	2	1	272		52	24	22	50	39	111	34	22	7	23
DDB_G0283381	DDB_G0283381 (SPC105)	max mid devel	max late G2	growth&devel	9.78	27.11		Mitosis/centromere	related to spc105 of budding yeast, kinetochore component	9	75	61	480	16	148	661	2640	248	3842	0.0000036359410947188	0.0297239817617549			1	1	3	8	76	141	118	196	124	151	5	18	4	13	859		50	14	8	56	37	94	31	21	4	13
DDB_G0288223	anapc3	max mid devel	max late G2	growth&devel	4.26	17.62		Mitosis/spindle checkpoint	anaphase promoting complex subunit 3; TPR repeat containing protein; in yeast, interacts with APC6, mutants of both overreplicate DNA, suggesting a role in proteolysis of Cdc6 	30	283	160	521	10	62	297	707	385	1685	0.0000367436172579966	0.524235920804028			0	0	3	13	23	63	84	153	51	115	3	18	5	18	549		77	62	39	107	74	242	55	49	29	28
DDB_G0286233	anapc6	mid + late devel	max late G2	growth&devel	3.5	1.94		Mitosis/spindle checkpoint	anaphase promoting complex subunit 6; TPR repeat containing protein; in yeast, interacts with APC3, mutants of both overreplicate DNA, suggesting a role in proteolysis of Cdc6	28	181	103	324	170	461	356	1091	840	1874	0.765787184732688	0.879898549182371			6	4	1	16	88	168	196	196	157	266	88	199	88	199	1672		32	14	13	61	38	147	23	18	12	14
DDB_G0280113	anapc5	max mid devel	too little data	growth&devel	5.43	21.29		Mitosis/spindle checkpoint	anaphase promoting complex subunit 5; the human protein, together with APC7, interacts with the transcriptional coactivator CBP/p300; Dictyostelium does not have obvious CBP or p300 	3	71	26	114	0	30	39	169	104	348	0.00347751819537853	0.71852174746101			0	5	5	18	19	65	55	77	21	37	3	7	6	6	324		18	7	4	13	9	40	8	4	3	8
DDB_G0292470	anapc7	max mid devel	max late G2	growth&devel	2.33	12.01		Mitosis/spindle checkpoint	anaphase promoting complex subunit 7; absent in yeast; the human protein, together with APC5, interacts with the transcriptional coactivator CBP/p300; Dicty does not have obvious CBP or p300  	14	280	41	281	6	35	91	402	335	815	0.000288551936623979	0.999998877031569			0	2	1	7	8	25	31	62	20	34	1	5	1	3	200		22	14	6	48	24	80	14	10	10	17
DDB_G0285223	anapc4	max mid devel	max late G2	devel only	1.43	2.18		Mitosis/spindle checkpoint	anaphase promoting complex subunit 4; yeast null is non-viable, no conditional phenotype beyond heat sensitivity; suggested to bind core APC ubiquitylating machinery to TPR-repeat subunits which mediate interaction with Cdh1, see Vodermeier et al, Current Biology 13, 1459-1468 (2003)	2	64	3	47	1	38	4	22	105	76	1	0.999998877031569			1	2	3	3	7	19	11	27	20	22	2	10	4	8	139		11	5	3	19	4	31	4	4	0	7
DDB_G0272775	anapc8	max mid devel	max late G2	devel only	1.74	6.07		Mitosis/spindle checkpoint	anaphase promoting complex subunit 8; TPR-repeat protein; in yeast binds to the N-terminus of Clb2p, but not if D-box is mutated, see Meyn et al, Arch Biochem Biophys 407, 189-195 (2002)	68	651	137	532	20	90	182	376	829	1227	0.0122308993787186	0.999998877031569			13	10	38	82	44	128	85	85	31	80	4	14	11	25	650		199	117	98	180	96	365	99	122	38	94
DDB_G0268992	anapc10	max mid devel	max late G2	growth&devel	6.02	10.4		Mitosis/spindle checkpoint	anaphase promoting complex subunit 10; the yeast protein Doc1 is nonessential but required for the association of D-box or KEN box-containing substrates with the APC, considered a processivity factor for the corresponding ubiquitylation reactions; see Passmore et al, EMBO Journal 22, 786-796 (2003)	1	23	6	74	3	16	41	154	43	275	0.00154375502964228	0.492536516855637			1	1	0	0	5	19	15	33	24	25	2	4	0	3	132		5	3	2	7	5	30	6	2	1	4
DDB_G0273201	DDB_G0273201 (Mad2)	max mid devel	max late G2	growth&devel	4.01	16.38		Mitosis/spindle checkpoint	highly similar to Mad2, part of the spindle checkpoint signalling pathway	11	201	40	459	3	14	43	341	229	883	0.000049105045125853	0.642173226353793			2	0	1	0	5	11	10	31	19	26	10	6	1	1	123		18	6	6	38	22	91	22	12	6	12
DDB_G0292676	bub1	max mid devel	max late G2	growth&devel	2.97	5.3		Mitosis/spindle checkpoint	orthologue of budding yeast Bub1,  which is recruited to the kinetochores and necessary for the spindle checkpoint 	103	610	293	998	32	217	263	707	962	2261	0.0384686071015585	0.999998877031569			2	3	2	15	34	120	161	255	95	183	5	22	5	11	913		146	91	78	268	153	544	107	91	56	86
DDB_G0292134	bub3	"repair"	max late G2	growth&devel	2.57	4.95		Mitosis/spindle checkpoint	orthologue of budding yeast Bub3,  which is recruited to the kinetochores and necessary for the spindle checkpoint 	76	598	266	610	25	75	175	297	774	1348	0.0238932758927753	0.999998877031569			17	19	6	20	27	92	102	151	30	157	4	38	8	38	709		137	64	25	143	86	334	119	86	41	72
DDB_G0285601	cdc20	"repair"	max late G2	growth&devel	4.71	16.09		Mitosis/spindle checkpoint	anaphase promoting complex substrate recognition subunit	290	1991	1733	3991	113	371	2600	4648	2765	12972	0.0000181088314425612	0.314619138937411			49	23	11	32	121	380	382	443	181	585	13	63	6	39	2328		421	147	72	438	178	795	252	294	99	186
DDB_G0278775	ube2c	max mid devel	max late G2	growth&devel	2.92	9.26		Mitosis/spindle checkpoint	putative orthologue of S. pombe Ubc11 and H. sapiens UBE2C, aka UbcH10, involved in the destruction of cyclins during mitosis	22	507	91	578	12	82	146	652	623	1467	0.00121104102209969	0.999998877031569			0	1	4	21	16	56	55	89	34	67	10	26	11	31	421		49	23	17	76	36	176	29	32	11	29
DDB_G0280995	mps1	max mid devel	max late G2	growth&devel	4.17	21.48		Mitosis/spindle checkpoint	homologous to monopolar spindle 1 of budding yeast, a dual-specificity kinase required for spindle pole body (SPB) duplication and spindle checkpoint function (Graf et al, Int Rev Cytol 241,155-202 (2004); see also Stucke et al, Chromosoma 113, 1-15 (2004)	39	350	180	716	23	81	639	1658	493	3193	0.00000388919961623071	0.556956126851167			4	5	2	4	32	75	66	114	47	69	6	22	7	11	464		80	37	23	89	44	170	50	38	16	37
DDB_G0290325	espl1	max mid devel	max late G2	growth&devel	3.36	2.77		Mitosis/spindle checkpoint	highly similar to separase (aka separin), a peptidase which cleaves cohesin at the metaphase-anaphase junction.  The separase inhibitor securin cannot be recognized in Dictyostelium discoideum.  In yeast Esp1 is also involved in spindle morphogenesis (Jensen et al, JCB 152, 27-40 (2001))	21	324	76	548	14	135	56	285	494	965	0.567908723226863	0.999998877031569			1	6	11	25	65	115	72	101	94	116	19	40	22	50	737		35	29	17	78	40	159	37	24	15	24
DDB_G0278987	DDB_G0278987	near flat	cold-activated?	devel only	1.69	4.6301033416153	?		similar to Pin2-interacting protein X1; microtubule-binding protein essential for faithful chromosome segregation. 	10	341	23	225	5	28	24	166	384	438	0.042190808391542	0.999998877031569			0	0	3	7	3	2	4	3	2	6	2	3	3	5	43		38	30	22	33	19	65	6	9	2	9
DDB_G0280249	DDB_G0280249 (ase1)	max mid devel	max late G2	growth only	3.01	1.92		Mitosis/cytokinesis	similar to protein regulator of cytokinesis PRC1, aka Ase1, midbody resident microtubule bundling protein required for spindle elongation, see Khmelinki and Schiebel, Cell Cycle 7, 283-286 (2008)	40	318	113	533	13	113	37	154	484	837	1	0.999998877031569			0	0	2	3	9	32	50	91	46	83	1	3	0	2	322		74	38	22	112	49	264	66	37	12	44
DDB_G0285931	dlpB	mid + late devel	max late G2	growth&devel	2.77	9.56		Mitosis/cytokinesis	dynamin-like protein which locates to cleavage furrow and is required for cytokinesis, see Miyagishima et al, Proc Natl Acad Sci USA 105, 15202–15207 (2008)	158	1011	398	1591	852	1290	6224	20080	3311	28293	0.000707109069285621	0.999998877031569			23	48	134	414	397	777	667	716	279	549	226	644	337	773	5984		297	211	151	392	209	848	199	195	73	134
DDB_G0271628	dlpC	max mid devel	max late G2	growth&devel	8.8	6.93		Mitosis/cytokinesis	dynamin-like protein which locates to cleavage furrow and is required for cytokinesis, see Miyagishima et al, Proc Natl Acad Sci USA 105, 15202–15207 (2008)	17	200	162	869	64	187	454	1669	468	3154	0.00268206402842525	0.0356725173779182			133	146	78	222	198	466	301	387	317	656	111	216	77	175	3483		59	24	17	31	30	81	47	26	17	44
DDB_G0267690	icpA (INCENP)	max mid devel	max late G2	growth&devel	4.34	14.13		Mitosis/cytokinesis	INCENP, chromosomal passenger complex component which recruits aurora kinase to the kinetochores and later to the central spindle, involved in kinetochore function and cytokinesis	48	954	251	1869	38	481	850	3961	1521	6931	0.000281046753967282	0.579994646127989			3	0	4	4	45	138	156	283	172	283	6	31	2	17	1144		167	96	60	267	141	606	111	99	41	88
																		 	 
DDB_G0290551	rblA	max mid devel	max late G2	growth&devel	13.77	22.32		Cell-cycle transcriptional network	single Dictyostelium retinoblastoma-family gene	5	140	97	713	10	93	312	1558	248	2680	0.00000978892969318214	0.0108622163635055			2	0	2	14	58	106	74	102	80	70	10	21	10	26	575		15	21	15	32	34	96	17	13	7	8
DDB_G0284129	e2f	max mid devel	weak max late G2	growth only	2.25	1.15		Cell-cycle transcriptional network	not substantially RblA-regulated; included here for comparison	41	200	90	243	118	307	181	322	666	836	1	0.999998877031569			49	27	73	96	195	452	346	451	162	435	41	181	88	237	2833		72	62	32	65	29	95	39	38	5	32
DDB_G0276799	tfdp2	max mid devel	max late G2	growth&devel	3.89	5.83		Cell-cycle transcriptional network	DP, dimerization partner of E2F; upregulation in rblA disruptant presumably reflects positive feedback activation of RblA target genes; E2F itself is only slightly (1.5-fold) upregulated in the rblA disruptant	5	121	26	188	39	153	243	1106	318	1563	0.00688302611848095	0.999998877031569			4	1	11	22	32	75	79	137	101	107	16	55	50	68	758		22	16	11	32	13	60	8	3	3	7
DDB_G0281519	lin54	max mid devel	max late G2	growth&devel	2.53	15.36		Cell-cycle transcriptional network	similar to lin54, subunit of LINC/DREAM complex which regulates expression of G2/M genes, see Schmit et al, FEBS Journal 276, 5703-5716 (2009)	13	74	27	113	6	63	153	469	156	762	0.000389105710061822	0.999998877031569			9	6	11	17	40	84	65	87	40	101	7	26	27	55	575		31	18	9	35	20	60	31	9	5	16
DDB_G0286665	lin9	max mid devel	max late G2	growth&devel	2.34	3.75		Cell-cycle transcriptional network	similar to lin9, implicated in activation of G2/M genes in mammals, see Osterloh et al, EMBO Journal 26, 144-157 (2007)	11	143	26	197	23	129	104	509	306	836	0.0962353622197538	0.999998877031569			6	15	17	44	40	88	46	76	40	64	12	29	25	43	545		26	23	6	41	17	63	21	12	3	14
DDB_G0290787	mybO	mid + late devel	max late G2	growth&devel	2.57	2.45		Cell-cycle transcriptional network?	myb-domain containing putatative transcription factor; possible interactor with LINC/DREAM complex (see above).  Mutual best BLAST hit with DUO3, a conserved protein in land plants, which in Arabidopsis male germ cells promotes entry into mitosis independently of CyclinB	48	375	122	519	154	546	424	1577	1123	2642	0.416420978448391	0.999998877031569			6	8	64	137	125	295	327	389	209	371	107	269	156	346	2809		65	41	26	83	40	146	47	37	18	46
DDB_G0277331	DDB_G0277331 (SMYD3)	max mid devel	too little data	devel  only	1.61	6.78		Cell-cycle transcriptional network	similar (mutual best BLAST hit) to a widely conserved histone lysine methyl transferase called ASHR1 in plants, but known as the Set and MYnd Domain-containing protein SMYD3 in metazoans (there are no convincing orthologues in yeast).   In humans SMYD3 is upregulated in the majority of colorectal and hepatic cancers; it is thought to participate in transactivating genes involved in cell cycle regulation (see Hamamoto et al, Nature Cell Biology 6, 731-740 (2004)). The protein binds to the motif CCCTCC, which is found in the promoters of many such genes in humans and many cell cycle genes in Dictyostelium (manuscript in preparation)	2	20	0	30	0	6	9	37	28	76	0.0307168105758886	0.999998877031569			0	1	0	2	5	8	1	16	9	10	1	0	1	1	55		2	0	1	3	2	0	2	1	1	3
DDB_G0280079	DDB_G0280079 (ada2)	mid + late devel	flat	growth&devel	2.41	2.4		Cell-cycle transcriptional network	similar (BLASTS both ways) to histone acetyltransferase complex SAGA/ADA, subunit ADA2 of yeast, which shows weak S-phase specific expression (Spellman et al, Mol Biol Cell 9, 3273-3297 (1998)); ADA2 interacts with the typeA histone acetyl transferase GCN5, which is conserved in Dictyostelium.	23	296	74	284	44	208	131	483	571	972	0.520273835434725	0.999998877031569			4	8	20	44	69	118	107	133	65	132	18	57	51	69	895		81	22	23	38	25	120	38	40	4	32
DDB_G0274491	DDB_G0274491 (pcc1)	max mid devel	too little data	growth&devel	2.16	8.82		Cell-cycle transcriptional network	similar to Cancer-Testis Antigen 1, also known as L-antigen or CTAG1/NY-ESO1, which is strongly expressed in fetal testis, adult spermatogonia, and premeiotic spermatocytes, and overexpressed in a wide variety of cancers (see Scanlan et al, Immunological Reviews 188, 22-32 (2002)); yeast homologue PCC1 is a transcription factor which recruits SAGA histone acetylase co-activator (Kisseleva-Romanova et al, EMBO Journal 25, 3576-3585 (2006).	1	32	2	39	1	4	6	51	38	98	0.0126862540428411	0.999998877031569			0	1	1	6	1	3	5	8	7	7	2	3	3	1	48		3	4	2	4	4	19	6	0	0	4
DDB_G0285869	DDB_G0285869 (C/EBP)	max mid devel	max late G2	growth&devel	4.3	11.94		Cell-cycle transcriptional network?	weakly similar to C/EBP epsilon of mouse. C/EBP (enhancer-binding protein) recognizes the sequence CCAAT. This sequence is significantly enriched  in the promoters of RblA-repressed genes (manuscript in preparation). C/EBP proteins are well known to synergize with Rb during terminal differentiation in vertebrates, see Charles et al, J Cell Biochem 83, 414-425 (2001).	72	450	370	877	21	152	400	1028	695	2675	0.00046732840276533	0.46444558638696			5	5	1	3	16	62	76	157	69	121	6	10	6	13	550		67	56	33	104	67	243	59	81	28	22
DDB_G0279459	DDB_G0279459 (Ki-67)	max mid devel	max late G2	growth&devel	3.72	20.44		Cell-cycle transcriptional network	forkhead-associated domain containing protein; the conserved domain is similar to that in the KI-67 antigen, a widely used clinical tumour proliferation marker, or the related protein chmadrin.  In humans KI-67 is present in proliferating cells during all phases of the cell cycle but it is absent in G0 cells.  Human KI-67 may be involved in the transcription of ribosomal RNA genes (Bullwinkel et al, J Cell Physiology 206, 624-635 (2005); Rahmanzadeh et al, Cell and Molecular Biology 40, 422-430 (2007)).  Expression profile of Dictyostelium gene is not similar to that of ribosomal proteins or ribosomal intersegments	177	1997	791	3388	120	634	3062	13068	2928	20309	0.00000413433875468428	0.73445707638835			43	19	38	92	213	753	346	615	367	706	34	96	12	50	3384		407	261	183	437	265	1021	267	235	90	201
DDB_G0269732	DDB_G0269732	max mid devel	max late G2	growth&devel	2.19	6.6		Cell-cycle transcriptional network?	apparent Dictyostelium-specific protein containining  dnaJ chaperone domain and putative methylated-histone-binding chromo domain, possibly involved in chromatin assembly or disassembly.  No similar domain structure in CDART database.  	197	1335	519	1324	87	369	813	1934	1988	4590	0.0047856858734872	0.999998877031569			34	19	52	85	19	85	53	105	44	84	16	21	27	64	708		294	150	83	258	127	546	177	147	67	156
DDB_G0290591	DDB_G0290591	peak late development	max late G2	growth&devel	2.71	4.24		Cell-cycle transcriptional network?	winged helix domain-containing putative transcription factor	45	550	132	743	807	2038	2122	15660	3440	18657	0.0427027843775474	0.999998877031569			19	27	130	410	271	500	430	605	460	588	327	578	630	1274	6249		148	98	53	116	83	311	96	65	27	86
DDB_G0280685	DDB_G0280685	max mid devel	too little data	growth&devel	4.86	88.59		Cell-cycle transcriptional network?	resembles the piwi domain of Dictyostelium argonaut proteins AgnA and AgnB; possible role in RNA interference	0	162	4	281	2	43	36	1793	207	2114	0.000000030709974568655	0.618071717574939			0	0	0	0	0	1	2	3	5	8	1	0	0	0	20		1	0	1	1	2	6	2	0	1	1
DDB_G0267958	DDB_G0267958	"repair"	max late G2	growth&devel	4.52	18.16		Cell-cycle transcriptional network?	bromodomain and forkhead-associated domain containing protein, similar to DDB_G0278469; the domain architecture is unique to Dictyostelium; some similarity to human protein polybromo of unknown function, but not the best hit for polybromo in the Dictyostelium proteome	43	682	225	1437	15	117	367	1858	857	3887	0.0000181088314425612	0.492536516855637			35	19	4	4	30	85	68	109	70	74	1	9	4	3	515		129	68	48	182	101	367	99	70	32	66
DDB_G0278469	DDB_G0278469	max mid devel	max late G2	growth&devel	3.11	5.73		Cell-cycle transcriptional network?	bromodomain and forkhead-associated/SMAD domain containing protein, similar to DDB_G0267958	31	486	79	915	10	77	68	471	604	1533	0.0127858422246699	0.999998877031569			2	2	1	2	6	20	18	29	21	22	2	4	2	5	136		56	38	20	106	59	216	42	41	12	22
DDB_G0285837	DDB_G0285837	mid + late devel	max late G2	growth&devel	2.47	5.5		Cell-cycle transcriptional network?	bromodomain containing, wd40 containing, similar to human wdr9, broadly expressed and dynamic during development, also to Drosophila ramshackle/brwd3 necessary for eye development, Mech Dev 123, 591-604 (2006), interacts with the Brahma-related gene 1, brg1	104	836	310	933	186	591	1152	3795	1717	6190	0.00953361397491115	0.999998877031569			113	39	167	307	166	313	253	375	256	389	85	210	169	364	3206		173	103	78	176	105	366	111	85	29	87
collective-DDB0215067_RTE					39.89	59.94		Collective retransposable element Skipper-GAG-PRO		275	963	8122	24423	97	230	6506	16202	1565	55253	0.00000000725961726579851	0.00000411049081860895
collective-DDB0216820_TE					9.53	61.34		Collective transposable element Tdd-4		108	1404	1036	7094	46	323	3814	17395	1881	29339	0.00000000862171034884488	0.0191886388562116
DDB_G0290955	transposon Tdd-5	max mid devel	too little data	growth&devel	85.14	44.83		Transposable element	putative transposase/transposition inhibitor gene from DNA transposon Tdd-5, this appears to be a single copy transposon which is related to Tdd-4	0	2	37	129	0	3	46	174	5	386	0.000000883148872766134	0.00000218899810017087			0	2	4	21	8	29	26	35	14	22	0	2	0	1	164		4	1	1	2	2	9	4	0	0	1
DDB_G0280011	DDB_G0280011_TE	flat	too little data	growth&devel	31.5964339768578	18.15		Transposable element	transposable element	0	2	1	58	0	1	1	46	3	106	0.00168704763178296	0.00627798677889858			2	0	4	1	2	1	1	2	4	0	2	0	1	1	21		0	2	1	0	0	0	0	1	0	1
DDB_G0277765	DDB_G0277765 (GAG)	flat	cold-activated?	growth&devel	20.05	8.58		Transposable element	retrotransposon skipper, diverged gag gene	1	8	26	41	3	2	20	20	14	107	0.00680063139163337	0.00534676781066014			7	15	7	14	3	13	9	1	1	15	0	11	2	15	113		1	5	1	0	0	1	0	0	0	0
DDB_G0277779	DDB_G0277779 (GAG)	peak early development	cold-activated?	growth&devel	10	6.28		Transposable element	retrotransposon skipper, diverged gag gene	9	49	69	296	7	13	35	120	78	520	0.0137299814946024	0.0294282885736798			9	49	36	39	20	33	35	25	9	22	7	18	6	34	342		2	7	8	2	1	4	3	0	1	0
DDB_G0267306	DDB_G0267306_RTE	flat	no data	growth&devel	24.928158376575727	6.68		Transposable element	retrotransposon skipper	0	1	3	22	0	0	5	11	1	41	0.00688302611848095	0.00534676781066014			0	2	0	0	0	0	0	0	0	0	0	2	0	0	4		0	0	0	0	0	0	0	0	0	0
DDB_G0287323	DDB_G0287323	early development	flat	devel only	1.19	6.53		Sexual differentiation	oxidoreductase, biosynthetic protein overexpressed in Dictyostelium gametes, see Muramoto et al, Mech Devel 120, 965-975 (2003)	9	73	13	39	31	108	254	706	221	1012	0.00457865655772958	0.999998877031569			11	13	8	11	9	8	6	8	5	2	1	6	4	8	100		7	5	2	7	5	20	8	5	2	8
DDB_G0269322	DDB_G0269322	growth phase + some throughout	max mid G2	growth&devel	8.66	6.55		Bacterial interaction	EGF domain containing protein	118	1750	948	8510	45	237	336	1786	2150	11580	0.00376630496832099	0.0292782197312934			29	41	10	33	5	7	23	8	13	11	8	12	8	19	227		248	106	75	111	43	247	137	72	23	189
DDB_G0283395	DDB_G0283395	late development	no data	devel only	1.07	5.83		HssA family	HssA family, see Vincente et al, BMC Microbiology 8, 1 (2008); most HssA family members are downregulated in rblA disruptant.	0	1	0	1	12	14	46	137	27	184	0.0307059140410749	0.999998877031569			0	0	0	0	0	0	0	0	11	53	6	78	5	14	167		0	0	0	0	0	0	0	0	0	0
DDB_G0287791	gcvH3	flat	flat	growth&devel	6.44	10.407585819155479		lycine cleavage system H-protein		1	16	5	64	1	2	4	44	20	117	0.0139753624469841	0.380793480011472			0	0	1	2	2	5	1	3	0	2	1	0	0	0	17		4	0	0	3	1	5	0	0	1	2
DDB_G0272688	DDB_G0272688	late development	cold-repressed?	growth&devel	3.32	12.09	development/proportioning	padA, required for expression of pstA, mutants express ecmB abnormally		1	29	5	32	0	14	21	137	44	195	0.00431946352645394	0.999998877031569			1	1	1	2	1	2	1	0	2	5	22	28	16	33	115		22	2	3	4	0	9	7	5	1	7
DDB_G0271916	rtoA	later development	irregular	growth&devel	2.67	2.34		development/proportioning	protein thought to regulate vesicle fusion, may link cell cycle to differentiation pathway choice (Wood et al Development 122, 3677-3685 (2006))	103	577	262	859	40	97	102	273	817	1496	0.518398906430179	0.999998877031569			34	116	91	220	64	89	158	165	78	196	62	296	89	287	1945		60	168	116	73	42	189	128	101	31	135
DDB_G0271232	DDB_G0271232_ps	max mid devel	flat	growth&devel	1.96	13.09	pseudogene	pseudogene	pseudogene similar to putative extracellular matrix protein	17	95	32	102	1	7	23	33	120	190	0.0118615626008305	0.999998877031569			1	1	2	4	3	7	15	14	1	13	0	4	1	3	69		21	15	7	8	9	26	11	3	0	9
DDB_G0280687	arrI_ps	flat	flat	growth&devel	2.63	16.079507822334804		pseudogene	pseudogene similar to ADP-ribosylation factor family protein	0	29	2	48	0	6	17	121	35	188	0.000136076743702294	0.999998877031569			0	0	1	2	0	0	1	1	1	0	0	0	0	0	6		0	0	1	0	3	1	1	2	1	0
DDB_G0283257	samkE_ps2	max mid devel	max late G2	growth only	7.83	4.31		pseudogene	pseudogene with resemblances to protein kinase genes	0	8	7	36	1	3	2	26	12	71	0.218270211144183	0.156613134529483			0	1	0	0	1	3	3	5	2	1	0	2	0	1	19		2	1	0	2	2	3	1	1	1	0
DDB_G0276451	DDB_G0276451 (SIVA)	max mid devel	max late G2	growth&devel	5.43	10.57		Conserved protein, cell-cycle role not understood	possible orthologue of SIVA, an E2F-induced proapoptotic protein in vertebrates, see Fortin et al, JBC 279, 28706-28714 (2004)	3	91	26	235	5	28	57	378	127	696	0.000394249558473038	0.393101819001763			0	0	1	1	4	7	16	20	10	18	1	3	1	3	85		14	7	4	20	14	44	8	9	1	7
DDB_G0267830	gemA	max mid devel	max late G2	growth&devel	3.82	9.23		Conserved protein, cell-cycle role not understood	mitochondrial anchored GTPase,  metazoan orthologue is called miro mitchondrial rho and is thought to be involved in the control of mitochondrial trafficking; Dictyostelium knockout shows no change in mitochondrial localization (Vlahou et al, European Journal of Cell Biology 90, 342-355 (2011))	28	220	79	535	7	62	93	439	317	1146	0.00233971644892731	0.79027748528771			2	4	13	60	30	75	49	109	40	69	8	9	9	20	497		82	61	51	124	70	270	47	44	26	57
DDB_G0290799	DDB_G0290799 (CARKD)	max mid devel	max late G2	growth&devel	3.41	8.18		Conserved protein, cell-cycle role not understood	highly similar (BLASTS both ways)  to conserved carbohydrate kinase-domain containing protein CARKD, unknown function.  	31	384	125	596	49	223	502	1817	687	3040	0.00121104102209969	0.958858852232473			6	8	8	22	63	186	161	205	75	196	15	56	27	100	1128		62	61	46	126	66	271	52	39	23	32
DDB_G0288129	DDB_G0288129	max mid devel	max late G2	growth&devel	2.55	5.14		Conserved protein, cell-cycle role not understood	BLASTs both ways to ACRC acidic repeat containing protein, conserved protein of unknown function (Nolte et al, Neurogenetics 3, 207-213 (2001)), Dictyostelium protein lacks acid repeats	94	790	269	966	25	131	178	523	1040	1936	0.028586771836661	0.999998877031569			2	8	7	19	8	33	30	52	15	28	1	9	4	20	236		84	78	46	117	66	251	53	66	12	38
DDB_G0267526	DDB_G0267526	max mid devel	max late G2	growth&devel	3.67	6.54		Conserved protein, cell-cycle role not understood	DNAJ family protein, orthologue of dnaj-23 of Caenorhabditis or dnaj-6 of maize, function unknown	12	779	60	1033	12	175	98	1065	978	2256	0.00849303560004693	0.999998877031569			3	1	13	55	21	78	31	49	33	37	5	10	6	13	355		57	31	23	112	46	235	40	37	15	26
DDB_G0284263	DDB_G0284263	max mid devel	max late G2	growth&devel	2.29	11.85		Conserved protein, cell-cycle role not understood	DNAJ heat shock N-terminal domain-containing protein, orthologue of human DNAJ (Hsp40) C11	58	990	157	1043	21	132	314	1553	1201	3067	0.000151906742961232	0.999998877031569			9	2	6	17	36	81	78	118	73	131	17	35	15	27	645		140	51	53	222	123	466	111	95	53	95
DDB_G0269968	DDB_G0269968	max mid devel	max late G2	growth&devel	2.26	12.18		Conserved protein, cell-cycle role not understood	similar (first BLAST hit, both ways) to Armadillo Repeat Containing Protein 1 (ARMC1) of Rattus norvegicus, D. purpureum orthologue also contains cell cycle element	160	1958	427	2008	38	198	581	2270	2354	5286	0.000121826684237657	0.999998877031569			10	15	22	73	100	313	202	297	101	265	21	59	24	66	1568		182	127	84	250	149	572	128	147	62	108
DDB_G0291838	DDB_G0291838 (xpo2)	max mid devel	max late G2	growth&devel	2.4	11.08		Conserved protein, cell-cycle role not understood	conserved in eukaryotes, similar to Cse1, which is required for accurate chromosome segregation in yeast; re-exports importin alpha	129	1377	346	1613	14	201	267	903	1721	3129	0.00456678202417197	0.999998877031569			32	13	13	39	83	198	181	261	124	229	6	41	15	54	1289		366	253	169	475	276	1024	236	203	95	174
DDB_G0285805	DDB_G0285805	max mid devel	max late G2	growth&devel	1.93	5.02		Conserved protein, cell-cycle role not understood	weakly similar (first BLAST hit, both ways) to an Oryza protein of unknown function, also found in other plants	19	170	27	209	10	53	64	260	252	560	0.0315607817922011	0.999998877031569			1	0	1	7	16	28	32	70	28	54	3	13	8	12	273		20	7	12	27	20	75	16	13	5	10
DDB_G0277011	DDB_G0277011 (Shaker)	"repair"	max late G2	growth&devel	2.09	11.89		Conserved protein, cell-cycle role not understood	BLASTs both ways to shaker-related voltage gated potassium channel.  This channel is strongly expressed in several classes of neurons in adult mammals, appears to regulate excitability; also expressed during embryonic development where it shows a correlation with proliferation (see Hallows and Tempel, Journal of Neuroscience 15, 5682-5691 (1998)).  Older work shows clear correlation between expression and proliferation in cultured Schwann cells, and blockade of potassium conduction inhibits proliferation (reviewed in op cit)	138	1080	342	1039	19	85	323	718	1322	2422	0.000213227666451732	0.999998877031569			11	23	9	11	18	69	91	120	45	127	3	9	1	15	552		230	136	74	279	127	448	161	126	43	89
DDB_G0289021	ube2s	"repair"	max late G2	growth&devel	2.22	21.04		Conserved protein, cell-cycle role not understood	Ube2s in humans is better known by the name of "E2-EPF UCP". The normal role of E2s is to provide activated ubiquitin moieties to protein-ubiquitin ligases (E3s) which use these to target intracellular proteins for proteasomal breakdown. E2-EPF UCP subverts this pathway, targeting an E3 it associates with for destruction. This protein is the von Hippel-Lindau tumour suppressor VHL, which normally targets the hypoxia-associated transcription factor HIFalpha. HIFalpha is required for tumour angiogenesis. Accordingly, overexpression of E2-EPF, which indirectly upregulates HIF-alpha, is associated with poor prognosis in certain cancers, and forced overexpression boosts tumour proliferation (see Jung et al, Nature Medicine 12, 809-816 (2006) or Ohh, Cancer Cell 10, 95-97 (2006)).  It is clear enough that tumour angiogenesis pathways cannot be conserved in Dictyostelium. However, an intriguing association of this E2 with proliferative activity remains	31	542	65	706	3	48	97	628	624	1496	0.0000732758777499943	0.999998877031569			6	1	1	1	5	19	31	35	16	29	1	3	1	1	150		72	14	11	85	38	198	31	19	16	20
DDB_G0278797	DDB_G0278797	"repair"	max late G2	devel only	1.65	8.21	conserved protein, cell cycle role not understood	NAD-dependent epimerase		81	535	149	431	18	53	194	404	687	1178	0.00142729292458006	0.999998877031569			54	41	23	40	51	170	87	109	29	81	4	39	10	38	776		100	81	52	107	57	209	65	76	28	68
DDB_G0274813 (vid27)	DDB_G0274813 (Vid27)	mid + late devel	max late G2	growth&devel	2.98	3.42		Conserved protein, cell-cycle role not understood	Highly similar (BLASTs both ways) to vacuolar import and degradation protein Vid27 of S. pombe, required for fructose utilization, also found in fungi and plants, no known role in cell cycle	178	1629	578	2415	250	1070	1087	3588	3127	7668	0.129269764248148	0.999998877031569			35	43	210	567	286	522	637	652	242	365	103	310	182	393	4547		274	216	138	333	201	714	207	182	70	140
DDB_G0289257	set1	later development	max late G2	devel only	1.91	4.52		Conserved protein, cell-cycle role not understood	orthologue of conserved histone H3K4 methyl transferase, see Chubb et al, Developmental Biology 292, 519 (2006), knockout of which leads to premature development; this phenotype is seen in the rblA disruptant, and the upregulation of this gene in the rblA- suggests a negative feedback attempt to restore normal development 	83	633	142	696	46	195	282	773	957	1893	0.0385180035933929	0.999998877031569			33	18	35	81	30	78	51	104	41	80	15	43	63	98	770		148	116	80	155	77	333	88	65	38	80
DDB_G0277693	DDB_G0277693	max mid devel	cold-activated?	growth&devel	7.63	6.31		Conserved protein, cell-cycle role not understood	similar (first identifiable BLAST hit, both ways) to cyr0, a putative cyanobacterial biosynthetic regulator, see Mihali et al, Applied and Environmental Microbiology 74, 716-722 (2008)	5	71	48	233	5	45	41	257	126	579	0.016324547187699	0.121932208883491			0	0	2	1	4	17	28	34	8	32	2	1	0	2	131		16	21	21	15	7	25	8	6	4	9
DDB_G0278369	DDB_G0278369	max mid devel	too little data	growth&devel	2.13	8.81		Conserved protein, cell-cycle role not understood	similar to (mutual best BLAST hit) to the tellurite-resistance gene tehB of various bacteria.  The bacterial protein apparently utilizes S-adenosyl methionine in the reduction of tellulrite to metallic tellurium.  There are only a handful of hits to eukaryotic sequences and these are significantly more diverged from the Dictyostelium gene than the bacterial sequences; possible laterally transferred gene.	2	58	4	69	1	9	15	34	70	122	0.0783852878417356	0.999998877031569			1	0	0	0	0	4	6	6	7	8	2	3	1	0	38		7	1	3	5	3	14	2	3	1	1
DDB_G0284191	DDB_G0284191	max mid devel	too little data	devel only	1.74	24.35		Conserved protein, cell-cycle role not understood	similar to the "Poxvirus D5 DNA primase" from Trichomonas vaginalis; but first BLAST hit is to the highly derived D5-like helicase-primase from Marseille Virus, which infects free-living amoebae (Proc. Natl. Acad. Sci. USA 106, 21848-21853 (2009)). Codon usage is typical of Dictyostelium.  Appears likely have entered Dictyostelium by horizontal transmission  but adopted role in Dictyostelium cell cycle.  The promoter contains the E2F-like element AATGGCGC (manuscript in preparation) 	0	9	1	11	1	7	36	122	17	170	0.000105233936336663	0.999998877031569			0	0	0	1	6	6	15	13	11	10	2	3	1	5	73		1	0	0	0	0	0	1	0	0	1
DDB_G0288015	arrH	mainly growth phase	too little data	devel only	2.05	12.55		Conserved protein, cell-cycle role not understood	ADP ribosylation factor, similar to human arf1, which is involved in golgi partitioning	1	14	3	10	0	12	22	55	27	90	0.0110627658186862	0.999998877031569			14	22	1	5	2	2	3	2	3	5	2	1	1	3	66		1	0	0	0	0	3	0	1	0	1
DDB_G0280621	arrJ	mainly growth phase	too little data	devel only	1.74	6.57		Conserved protein, cell-cycle role not understood	ADP ribosylation factor, similar to human arf1, which is involved in golgi partitioning	0	19	3	8	4	15	21	156	38	188	0.00801648351396749	0.999998877031569			14	15	1	4	1	2	2	1	2	3	1	0	1	0	47		0	0	0	0	0	1	0	0	0	2
DDB_G0280633	arrK	growth phase + some throughout	flat	devel only	1.21	14		Conserved protein, cell-cycle role not understood	ADP ribosylation factor, similar to human arf1, which is involved in golgi partitioning	0	22	1	16	1	23	23	163	46	203	0.00562887635880237	0.999998877031569			2	5	0	3	1	0	1	3	1	2	0	3	0	3	24		1	1	0	0	0	1	1	0	0	0
DDB_G0291822	wdr92	early and mid devel	flat	devel only	1.29	10.91		Conserved protein, cell-cycle role not understood	the one upregulated gene among 25 WDRs in Dictybase, in humans also known as Monad, which potentiates apoptosis in response to TNF-alpha, see Saeki et al Biochem Biophys Res Commun 342, 568-572 (2006); mechanism unknown; Monad interacts with RPAP3 which independently interacts with reptin	9	114	11	81	1	20	17	134	144	243	0.0119225320436202	0.999998877031569			4	3	4	25	7	11	5	17	4	8	1	0	1	2	92		30	10	5	10	6	40	16	7	3	11
																																						0
DDB_G0295827	DDB_G0295827	max mid devel	max late G2	growth&devel	3.11	10.05		Protein with conserved domain 	contains leucine rich repeats similar (BLAST both ways) to those found in NLR family proteins, which are innate immune response proteins in plants and animals; these proteins also contain other domains but these domains are not obviously present	30	145	88	251	8	44	126	260	227	725	0.0014640498531049	0.999998877031569			0	10	3	7	20	55	53	45	18	49	2	15	3	12	292		67	19	8	70	31	109	41	37	6	38
DDB_G0292880	DDB_G0292880	"repair"	max late G2	growth&devel	3.08	7.78		Protein with conserved domain 	dual specificity protein phosphatase similar to DDB_G0285449, which is 2.5-fold upregulated in the rblA disruptant.   	24	367	86	527	4	39	44	242	434	899	0.00833337836937013	0.999998877031569			7	1	0	2	8	9	20	40	14	34	0	0	1	0	136		63	34	24	96	44	207	34	28	16	22
DDB_G0290469	pks28	terminal differentiation	max late G2	growth&devel	4.14	16.85		Protein with conserved domain 	polyketide synthase expressed mainly in terminal differentiation	3	20	3	69	1	9	26	96	33	194	0.00115710604249299	0.966644555480039			0	1	0	2	3	8	7	12	16	14	4	0	105	167	339		9	5	3	18	8	29	2	3	1	9
DDB_G0275475	DDB_G0275475	mainly growth phase	cold-repressed?	growth&devel	13.04	6.8		Protein with conserved domain 	tenascin relative family	41	343	519	2435	9	43	88	225	436	3267	0.00833337836937013	0.00404587461851823			196	102	18	23	5	13	9	13	3	4	2	6	1	3	398		61	18	9	15	9	45	29	12	4	39
DDB_G0272368	DDB_G0272368	max mid devel	max late G2	growth&devel	4.37	5.94		Protein with conserved domain 	contains 3 SMC domains (chromosome separation ATPases) but is otherwise unique to Dictyostelium	9	367	42	812	12	211	97	1086	599	2037	0.0263815611853364	0.999998877031569			0	0	1	2	13	36	53	86	109	88	5	8	4	1	406		40	22	20	82	60	222	40	14	7	25
DDB_G0289761	DDB_G0289761	"repair"	max late G2	growth&devel	3.86	6.8		Protein with conserved domain 	BRCT and SMC-domain-containing protein, no significant homologies	30	393	144	663	52	232	418	1725	707	2950	0.0032757357241378	0.738976110208298			195	75	64	141	191	370	317	517	395	577	80	158	81	163	3324		75	46	33	78	47	167	55	34	11	37
DDB_G0290373	DDB_G0290373	max mid devel	max late G2	growth&devel	4.5	15.33		Protein with conserved domain 	contains leucine rich repeat domain, ribonuclease inhibitor subfamily, no significant homology	2	26	5	83	2	14	36	237	44	361	0.000156070096955913	0.642173226353793			0	0	1	6	8	15	18	36	39	14	2	3	0	3	145		6	2	1	17	4	15	4	6	0	1
DDB_G0278431	DDB_G0278431	max mid devel	max late G2	growth&devel	3.32	17.66		Protein with conserved domain 	contains conserved domain DUF2418 of unknown function; no significant homology; D. purpureum gene contains smyd3 binding site	24	303	90	483	10	122	290	1094	459	1957	0.000137924556145377	0.999998877031569			1	4	13	39	40	70	33	71	34	37	4	11	13	35	405		42	29	19	74	26	107	32	22	10	15
DDB_G0270072	DDB_G0270072	mid + late devel	max late G2	growth&devel	3.22	29.06		Protein with conserved domain 	possible SMC, see Dictybase annotation; has conserved mitotic destruction box	3	109	10	182	3	81	128	1716	196	2036	0.00000539282314761753	0.999998877031569			1	0	0	2	17	32	36	50	22	30	4	7	36	20	257		14	8	4	17	5	22	13	5	1	2
DDB_G0286625	DDB_G0286625	later development	too little data	growth&devel	10.74	32.12		Protein with conserved domain 	similar to aprA, a negative regulator of cell proliferation	0	1	5	8	0	4	42	120	5	175	0.00000498416274492394	0.155697635321218			0	0	0	2	2	2	1	0	3	0	2	4	0	1	17		0	1	0	1	0	0	1	0	0	0
DDB_G0270652	DDB_G0270652	flat	too little data	growth&devel	3.22	14.9501176213337		Protein with conserved domain 	contains molybdenum cofactor sulfurase domain	0	7	0	21	0	0	7	30	7	58	0.000218995165644416	0.999998877031569			1	0	1	1	0	0	1	0	0	0	0	0	0	0	4		2	0	0	1	0	2	2	0	0	0
DDB_G0295701	DDB_G0295701	minimum  in aggregation	too little data	growth&devel	18.23	4.09		Protein with conserved domain 	similar to P. pallidum cell surface glycoprotein	0	10	18	98	0	7	7	12	17	135	0.60463087846853	0.00373846859551694			53	47	8	17	53	37	52	45	25	33	15	45	42	90	562		0	0	0	1	0	0	1	0	0	1
DDB_G0279501	DDB_G0279501	max mid devel	max late G2	growth&devel	2.46	11		?	conserved in Polysphondylium; no further significant homology	30	293	60	437	17	85	266	737	425	1500	0.000347257942735167	0.999998877031569			7	5	8	26	65	143	96	126	64	142	16	52	18	41	809		55	37	22	81	32	167	23	21	15	31
DDB_G0285183	DDB_G0285183	max mid devel	max late G2	growth&devel	6.42	6.38		?	conserved in Polysphondylium; no further significant homology	30	238	256	665	43	151	343	924	462	2188	0.00502019180555455	0.11409050519088			2	3	2	5	19	59	45	104	46	74	5	9	7	2	382		81	33	21	100	44	198	34	40	14	28
DDB_G0284147	DDB_G0284147	max mid devel	too little data	growth&devel	2.64	8.413237047435382		?	conserved in Polysphondylium; no further significant homology	0	7	0	21	0	0	7	30	7	58	0.000218995165644416	0.999998877031569			1	0	1	0	4	4	10	10	2	6	0	1	1	1	41		4	1	0	2	1	7	0	0	0	0
DDB_G0278989	DDB_G0278989	flat	too little data	growth&devel	2.85	20.431270748641737		?	conserved in Polysphondylium; no further significant homology	0	4	1	9	0	0	8	43	4	61	0.0000564937225472565	0.999998877031569			0	0	0	1	0	0	0	0	0	0	0	0	0	0	1		0	0	0	1	1	2	0	0	0	0
DDB_G0287675	DDB_G0287675	max mid devel	too little data	growth&devel	13.97	11.367633435569344		?	conserved in Polysphondylium; no further significant homology	0	2	0	26	0	3	1	85	5	112	0.000738955309301313	0.135889460376665			0	0	0	0	1	3	4	5	8	2	0	1	0	0	24		1	1	0	2	0	1	0	0	0	0
DDB_G0295701	DDB_G0295701	minimum  in aggregation	too little data	growth&devel	18.230000377938737	4.0912067421187155		?	conserved in Polysphondylium; no further significant homology	0	10	18	98	0	7	7	12	17	135	0.60463087846853	0.00373846859551694			54	48	8	17	55	37	54	48	28	34	18	46	43	92	582		0	0	0	1	0	0	1	0	0	1
DDB_G0279499	DDB_G0279499	max mid devel	max late G2	growth&devel	2.31	5.44		?	no significant homology	13	172	32	201	11	67	83	358	263	674	0.0188870026228852	0.999998877031569			6	1	3	7	30	33	41	52	14	48	9	19	22	16	301		17	8	4	41	22	95	7	15	5	14
DDB_G0292292	DDB_G0292292	max mid devel	max late G2	growth&devel	7.53	11		?	no significant homology	15	108	77	530	12	82	181	752	217	1540	0.000436074727761996	0.0980431347134713			1	1	3	1	10	32	19	41	11	23	3	10	4	10	169		21	5	3	19	20	56	10	11	5	5
DDB_G0270920	DDB_G0270920	max mid devel	max late G2	growth&devel	2.4	7.37		?	no significant homology	49	1095	142	1181	51	285	416	2499	1480	4238	0.00166356597092734	0.999998877031569			3	2	5	11	16	44	74	121	87	107	26	40	32	43	611		90	81	58	183	71	309	69	54	25	40
DDB_G0272921	DDB_G0272921	max mid devel	max late G2	growth&devel	3.64	20.81		?	no significant homology	2	58	3	162	0	18	18	562	78	745	0.00000414649162498288	0.691645145636488			0	0	0	0	0	4	5	5	2	4	0	0	1	0	21		3	0	3	20	5	19	5	4	1	3
DDB_G0287115	DDB_G0287115	max mid devel	max late G2	growth&devel	4.69	19.82		?	no significant homology	10	182	53	410	3	46	94	494	241	1051	0.000150813645100879	0.498050027832424			0	2	1	6	13	19	32	56	21	31	3	7	4	10	205		33	16	7	40	26	78	17	21	7	7
DDB_G0291806	DDB_G0291806	max mid devel	max late G2	growth&devel	7.52	24.67		?	no significant homology	21	226	175	833	24	149	813	3127	420	4948	0.00000258780179535586	0.0747603205223514			0	0	2	7	28	72	57	115	35	66	3	5	0	4	394		41	24	14	80	23	108	33	27	14	16
DDB_G0286379	DDB_G0286379	max mid devel	max late G2	growth&devel	3.56	22.17		?	no significant homology	47	496	219	722	18	74	434	1983	635	3358	0.00000284683902342628	0.83821148051417			5	1	12	16	38	133	85	113	31	73	4	14	5	18	548		72	55	48	139	49	268	46	48	22	32
DDB_G0278073	DDB_G0278073	max mid devel	max late G2	growth&devel	4.36	10.1		?	no significant homology	49	381	190	959	26	148	344	1387	604	2880	0.000430255563852425	0.511445515598739			3	0	1	2	16	50	55	81	46	86	29	35	15	35	454		70	36	29	98	34	167	46	35	11	25
DDB_G0278825	DDB_G0278825	max mid devel	max late G2	growth&devel	2.47	5.91		?	no significant homology	286	4155	775	5072	104	825	814	4516	5370	11177	0.0100060147812041	0.999998877031569			4	5	2	18	52	254	151	299	171	277	7	37	3	17	1297		335	193	126	554	297	1140	276	267	98	176
DDB_G0283999	DDB_G0283999	max mid devel	max late G2	growth&devel	3.8	11.68		?	no significant homology	34	154	128	314	15	38	253	385	241	1080	0.000151906742961232	0.717792344747203			3	3	1	5	5	18	23	24	17	35	3	1	0	4	142		35	27	20	66	26	123	22	28	13	9
DDB_G0286591	DDB_G0286591	max mid devel	max late G2	growth&devel	5.16	11.54		?	no significant homology	9	70	35	228	10	52	135	666	141	1064	0.000197862105850897	0.397289289182609			2	0	3	13	35	57	51	62	36	44	4	13	5	10	335		12	3	7	27	5	45	11	5	2	14
DDB_G0286745	DDB_G0286745	max mid devel	max late G2	growth&devel	4.49	18.94		?	no significant homology	9	398	46	822	14	83	213	2337	504	3418	0.00000414649162498288	0.641679202043591			0	2	1	10	10	29	18	32	25	36	8	20	6	15	212		39	22	14	82	56	216	35	27	8	29
DDB_G0288059	DDB_G0288059	max mid devel	max late G2	growth&devel	3.72	20		?	no significant homology	26	387	115	658	5	53	145	798	471	1716	0.0000318473672307214	0.829396312975294			0	0	0	1	7	16	28	32	11	23	0	1	0	2	121		20	13	9	51	26	86	12	11	5	13
DDB_G0288125	DDB_G0288125	max mid devel	max late G2	growth&devel	4.97	26.56		?	no significant homology	20	401	110	970	12	75	338	2431	508	3849	0.00000121604465130494	0.393101819001763			0	0	0	3	13	53	47	97	35	59	1	8	4	2	322		27	17	13	94	49	204	29	29	11	23
DDB_G0282123	DDB_G0282123	max mid devel	max late G2	growth&devel	3.76	29.66		?	no significant homology	5	140	20	267	2	37	97	566	184	950	0.0000271152387643893	0.909437852863691			3	4	1	5	11	24	23	33	8	19	4	10	5	4	154		21	5	2	24	11	48	9	13	5	13
DDB_G0289673	DDB_G0289673	max mid devel	max late G2	growth&devel	3.57	25.91		?	no significant homology	17	307	76	473	5	77	206	1145	406	1900	0.000017342622636276	0.987472477550626			0	0	1	4	25	59	69	108	51	100	1	10	5	12	445		39	18	16	55	26	141	34	20	10	18
DDB_G0287431	DDB_G0287431	max mid devel	max late G2	growth&devel	6.67	8		?	member of Dictyostelium family, no significant homology outside Dictyostelium	0	31	10	69	0	1	3	17	32	99	0.0384686071015585	0.492536516855637			0	0	0	0	0	0	1	0	2	3	0	0	1	0	7		3	1	0	5	2	16	1	3	0	1
DDB_G0285671	DDB_G0285671	max mid devel	max late G2	growth&devel	6.57	26.68		?	no significant homology	3	63	14	268	1	18	38	354	85	674	0.0000367436172579966	0.163232253630978			0	0	0	3	1	3	9	13	7	11	0	0	0	0	47		6	5	2	16	10	40	5	6	1	5
DDB_G0269180	rcdP	max mid devel	max late G2	growth&devel	5.74	12.57		?	no significant homology, named for "random cDNA clone"	46	531	309	1415	60	313	984	3794	950	6502	0.0000922467997224515	0.205060494090265			9	1	7	5	48	150	260	375	148	255	29	66	42	100	1495		98	56	44	180	122	431	97	68	27	57
DDB_G0267750	DDB_G0267750	max mid devel	max late G2	growth&devel	5.59	7.64		?	member of small Dictyostelium gene family, no significant homology outside Dictyostelium	0	103	10	126	1	20	11	117	124	264	0.0348947909409979	0.999998877031569			0	1	1	2	0	1	8	12	11	14	1	1	1	0	53		10	14	9	14	8	41	4	4	1	5
DDB_G0274947	DDB_G0274947	max mid devel	max late G2	growth&devel	4.18	32.51		?	similar to DDB_G0290019, which is not significantly RblA-regulated, otherwise no significant homology	20	577	91	1200	14	195	639	5126	806	7056	0.00000110181427088572	0.641679202043591			0	0	4	3	47	98	94	144	82	115	14	17	22	26	666		35	19	16	94	48	177	37	22	11	21
DDB_G0288329	DDB_G0288329	mid + late devel	max late G2	devel only	1.9	16.43		?	no significant homology	70	1010	149	930	55	190	848	4451	1325	6378	0.0000123933464832284	0.999998877031569			11	10	6	46	27	106	68	120	79	128	59	73	80	140	953		97	79	50	120	76	262	80	57	30	57
DDB_G0290301	DDB_G0290301	mid + late devel	max late G2	growth&devel	2.3	6.75		?	no significant homology	81	510	156	703	26	122	232	755	739	1846	0.00457865655772958	0.999998877031569			12	4	12	15	36	129	70	139	58	117	11	59	45	112	819		134	117	52	140	61	258	63	62	23	52
DDB_G0284115	DDB_G0284115	"repair"	max late G2	growth&devel	4.51	9.73		?	no significant homology	14	371	72	800	5	33	60	387	423	1319	0.000670822247900684	0.55158530969996			18	11	4	12	23	40	17	42	17	40	0	7	3	3	237		24	25	16	35	19	90	21	18	5	17
DDB_G0287991	DDB_G0287991	growth + mid devel	max late G2	growth&devel	4.65	26.92		?	no significant homology	73	993	424	2040	19	144	768	2943	1229	6175	0.00000246285356288029	0.447397075124132			472	68	14	49	60	153	100	151	60	157	2	21	12	24	1343		138	112	71	218	130	486	119	93	51	106
DDB_G0274435	DDB_G0274435	max mid devel	cold-activated?	growth&devel	3.23	13.98		?	conserved in Polysphondylium; no further significant homology	3	96	15	98	2	35	45	269	136	427	0.00190201502120057	0.999998877031569			0	0	1	2	9	21	25	26	20	27	3	7	5	14	160		13	17	9	9	8	25	9	10	0	9
DDB_G0275487	DDB_G0275487	max mid devel	cold-activated?	devel only	2.15	4.36		?	no significant homology	0	12	0	24	76	690	354	3731	778	4109	0.0263815611853364	0.999998877031569			3	4	265	863	250	340	1341	2066	1053	1369	184	331	119	251	8439		0	2	6	3	0	7	2	0	0	0
DDB_G0290897	DDB_G0290897	max mid devel	too little data	growth&devel	6.28	9.11		?	member of small Dictyostelium gene family, no significant homology outside Dictyostelium	0	16	7	49	0	4	8	54	20	118	0.00352983346233389	0.340174534421176			0	0	3	1	1	3	6	15	5	9	0	1	0	1	45		2	2	0	2	1	9	2	1	1	0
DDB_G0278513	DDB_G0278513	max mid devel	too little data	devel only	0	14.72		?	member of small Dictyostelium gene family, no significant homology outside Dictyostelium	0	0	0	0	3	22	35	169	25	204	0.00395888130343853	0.999998877031569			0	0	0	0	2	1	16	32	30	56	9	17	5	10	178		0	0	0	1	0	0	0	0	0	1
DDB_G0272642	DDB_G0272642	mid + late devel	too little data	devel only	1.61	4.58		?	member of Dictyostelium gene family, no significant homology outside Dictyostelium	0	6	0	9	51	149	230	918	206	1157	0.0285499912954058	0.999998877031569			0	0	21	49	190	110	180	123	190	73	88	68	191	142	1425		0	0	0	1	1	0	0	0	0	0
DDB_G0272640	DDB_G0272640	mid + late devel	cold-activated?	growth&devel	7.73	11.92		?	no significant homology	0	15	5	78	6	32	66	544	53	693	0.000139678192593128	0.120275187728038			0	2	3	10	15	14	24	26	15	11	15	10	17	15	177		5	5	3	3	3	15	4	4	1	0
DDB_G0273351	DDB_G0273351	max mid devel	max late G2	growth&devel	13.06	8.018770708244515		?	no significant homology	1	4	1	47	0	4	5	58	9	111	0.00447337601992428	0.155141033723052			0	0	0	2	13	3	8	14	10	2	3	1	0	0	56		1	0	0	2	1	2	0	0	0	1
DDB_G0286807	DDB_G0286807	max mid devel	max late G2	growth&devel	2.92	4.175890102432663		?	no significant homology	0	14	0	38	0	5	2	49	19	89	0.0384686071015585	0.999998877031569			0	0	0	0	0	1	3	7	3	2	0	1	0	0	17		2	0	0	2	3	4	2	0	0	1
DDB_G0285173	DDB_G0285173	max mid devel	flat	growth&devel	3.57	8.769313297659327		?	no significant homology	0	13	1	38	0	10	7	139	23	185	0.00115758081047498	0.829323516307711			0	0	1	1	3	9	7	8	13	9	2	1	0	3	57		0	0	0	1	1	2	1	0	0	1
DDB_G0271468	DDB_G0271468	growth + mid devel	too little data	growth&devel	6.35	7.741705882755071		?	no significant homology	0	7	3	33	0	3	3	49	10	88	0.00642566922240321	0.29052920670834			11	0	0	0	5	2	0	1	11	5	0	0	1	0	36		1	4	2	5	0	5	1	0	0	0
DDB_G0279531	DDB_G0279531	max mid devel	cold-repressed?	growth&devel	12.09	11.2		?	no significant homology	0	13	7	110	1	5	10	82	19	209	0.00260314539962057	0.0189243192018842			0	0	0	0	1	1	3	0	0	0	0	0	0	0	5		3	0	0	2	1	6	1	1	1	3
DDB_G0278865	DDB_G0278865	growth phase + some throughout	cold-repressed	growth&devel	3.97	4.52		?	member of small Dictyostelium gene family, no significant homology outside Dictyostelium	66	276	300	520	77	130	431	602	549	1853	0.0286003419825015	0.535214729740974			85	196	33	39	23	54	60	28	7	54	7	114	19	78	797		99	7	7	59	25	78	33	31	16	46
DDB_G0273289	DDB_G0273289	minimum early devel	too little data	growth&devel	4.28	4.34		?	no significant homology	3	11	6	35	19	89	99	409	122	549	0.0471624336183038	0.999998877031569			26	58	12	31	13	17	53	14	24	38	16	92	27	55	476		3	0	0	2	0	6	1	1	0	4
DDB_G0293954	DDB_G0293954	flat	too little data	devel only	1.88	53.62		?	no significant homology	1	23	1	31	1	3	22	327	28	381	0.000000883148872766134	0.999998877031569			0	2	1	2	0	4	3	3	2	2	1	3	0	3	26		1	3	0	1	3	6	0	3	0	1
DDB_G0285929	DDB_G0285929	late development	too little data	devel only	3.76	11.94		?	no significant homology	0	2	0	7	1	3	9	55	6	71	0.00394852166642903	0.999998877031569			0	0	0	0	0	0	2	1	3	1	2	0	2	9	20		0	0	0	2	0	0	0	0	0	0
DDB_G0288279	DDB_G0288279	weak max mid devel	too little data	growth&devel	3.64	8.1		?	no significant homology	6	15	21	30	0	3	10	25	24	86	0.921958696089969	0.0123809545235683			0	2	1	2	3	3	3	3	2	2	0	1	0	2	24		1	1	0	4	0	1	1	4	0	1
DDB_G0292936	DDB_G0292936	flat	too little data	devel only	1.94	9.68		?	no significant homology	3	7	5	8	1	0	8	15	11	36	0.0176270563207201	0.999998877031569			3	3	5	8	2	11	6	5	1	16	1	13	1	3	78		0	0	0	0	0	1	1	0	0	0
DDB_G0287885	DDB_G0287885	max mid devel	max late G2	growth&devel	2.64	6.76003504689654		?	no significant homology	5	119	12	179	0	9	9	55	133	255	0.0289606700358308	0.999998877031569			1	0	0	0	2	5	8	8	2	8	0	1	0	2	37		1	2	2	8	5	11	2	1	0	2
ribosomal intersegments
rDNA intersegment 1		near flat	cold-repressed		1.31	1.61		non-rblA regulated comparison sequence	terminal segment of Dictyostelium rDNA palindrome	49737	19075	55244	14750	13614	1389	14009	3994	83815	87997	1	0.999998877031569			71161	38925	143770	47853	51844	76476	86625	24860	39514	68033	33366	28151	64258	71137	845973		53892	22187	11744	33243	13787	55897	34838	25218	9508	39792
rDNA_intersegment_2		growth and early development	near flat	growth&devel	3.36	4.91			rDNA between 26s and 5.8s genes	1483	1033	3253	2324	171	94	1077	446	2781	7100	0.0132158936693759	0.999998877031569			1738	1164	3658	909	925	852	998	335	509	572	311	327	583	666	13547		1809	1192	624	796	302	1265	689	617	224	729
rDNA_intersegment_3		too little data	too little data	growth&devel	10.6	7.27			rDNA between 5.8s and 17s genes	1	3	3	26	0	0	7	10	4	46	0.00394852166642903	0.296423508773886			0	2	9	10	4	5	2	3	3	7	1	2	3	5	56		0	1	1	1	0	5	0	0	0	0
rDNA_central_segment		growth and early development	near flat	growth&devel	4.008859394568475	20.644533372399298			central section of rDNA palindrome, normally little transcribed, transcription strongly upregulated in rblA disruptant	1207	1285	3442	3335	242	276	5685	6559	3010	19021	0.00000374456714657185	0.999998877031569			1605	1683	4359	720	1075	1782	1537	487	732	928	529	510	687	798	17432		1088	1348	371	548	113	580	513	565	131	620
DDB_G0279085	cycA	max mid devel	max late G2	not subst. regulated	1.49	0.36		comparison sequence	putative cell-cycle cyclin	86	818	155	558	111	407	52	141	1422	906	0.9017230559421	0.999998877031569			74	42	53	169	141	438	352	480	113	396	31	176	63	172	2700		103	96	79	92	62	198	84	65	19	66
DDB_G0275493	cycB	max mid devel	max late G2	not subst. regulated	1.81	0.53		comparison sequence	putative cell-cycle cyclin	408	1950	761	1835	166	474	122	212	2998	2930	1	0.999998877031569			39	37	24	52	39	163	477	969	341	1192	14	123	26	66	3562		945	740	441	1130	498	2131	963	722	260	487
DDB_G0277439	cycD	mid + late devel	cold-activated	growth only	2.57	0.53		comparison sequence	putative cell-cycle cyclin	2	92	7	92	239	1615	170	763	1948	1032	1	0.999998877031569			2	6	51	191	403	578	566	681	339	522	120	280	186	323	4248		2	8	7	5	1	5	3	1	2	4
DDB_G0283617	cdc25	max mid devel	max late G2	not subst. regulated	1.59	0.32		comparison sequence	CDK activating phosphatase	118	671	205	535	322	895	130	286	2006	1156	0.783340293531111	0.999998877031569			63	29	32	63	60	86	186	276	558	966	105	228	118	268	3038		232	84	53	236	105	483	118	100	44	78
DDB_G0287259	cdh1	later development	near flat	not subst. regulated	1.42	0.95		comparison sequence	APC specificity subunit, in metazoans active in mitotic exit and G1	110	513	155	391	1028	1690	1000	2095	3341	3641	1	0.999998877031569			153	111	149	307	165	317	516	405	273	652	190	576	236	783	4833		95	51	39	39	28	104	42	42	15	47
DDB_G0277503	amtA	later development	min late G2	not subst. regulated	1	0.86		comparison sequence	ammonia permease, exports ammonium from cells	919	4770	1048	2289	406	1006	482	723	7101	4542	1	0.999998877031569			452	359	112	294	703	1038	1225	941	370	997	275	1117	709	2432	11024		1860	1242	840	439	206	843	666	411	150	820
DDB_G0289243	fnip	"repair"	max late G2	not subst. regulated	1.1834063049026677	0.6821290879710811		comparison sequence	putative folliculin-interacting protein	27	357	31	230	21	110	18	75	515	354	1	0.999998877031569			11	15	11	28	33	69	71	108	54	103	8	36	19	30	596		50	35	19	70	26	105	13	20	5	19
DDB_G0269388	corB	max early devel	max late G2	not subst. regulated	1.0069802821367615	0.7568661779660192		comparison sequence	orthologue of human coronin 7	368	2708	413	1328	73	199	67	159	3348	1967	1	0.999998877031569			118	125	110	340	49	119	73	137	49	92	47	113	38	125	1535		558	302	215	454	211	937	300	265	106	245
DDB_G0271134	celA	terminal differentiation	cold-repressed?	rblA-activated during develop.	0.8461581341899433	0.21444967068127			cellulase 270-6	39	261	46	83	315	161	29	71	776	229	0.0164133909217916	0.999998877031569			39	7	9	33	7	7	10	15	8	18	8	50	626	1480	2317		194	58	24	29	14	65	38	31	12	47
DDB_G0271178	DDB_G0271178	terminal differentiation	too little data	rblA-activated during develop.	3.2243604717229695	0.10091609244205503				0	0	0	3	452	698	19	146	1150	168	0.00199269952558908	0.889782542581923			0	1	0	0	0	0	0	0	6	11	135	354	537	1320	2364		0	0	0	0	0	0	0	0	0	0
DDB_G0271196	DDB_G0271196	terminal differentiation	too little data	rblA-activated during develop.	5.373934119538283	0.07080841508791004				0	0	0	5	263	736	10	100	999	115	0.00113159139765314	0.492536516855637			0	0	0	0	1	1	0	2	8	15	117	261	510	1089	2004		0	2	1	0	0	3	0	0	0	0
DDB_G0271314	iliH	terminal differentiation	too little data	rblA-activated during develop.	1.3253793768812412	0.050531099167063914			Endo-1,4-beta-glucanase family protein, glycoside hydrolase family 9 protein	2	11	3	7	59	15	3	1	87	14	0.000387511422136371	0.999998877031569			9	3	7	8	7	2	5	5	1	2	2	5	511	1328	1895		1	0	1	1	0	3	0	0	0	0
DDB_G0273155	DDB_G0273155	terminal differentiation	too little data	rblA-activated during develop.	0	0.25053499278375096			zinc-containing alcohol dehydrogenase (ADH)	0	2	0	0	236	77	3	49	315	52	0.000505687194153467	0.999998877031569			0	0	0	0	9	2	2	2	14	56	22	88	1766	3632	5593		0	0	0	1	0	0	0	0	0	0
DDB_G0274335	DDB_G0274335	terminal differentiation	too little data	rblA-activated during develop.	1.3949242250526521	0.0690718787691065				1	10	1	9	311	179	3	30	501	43	0.00000859674812131498	0.999998877031569			0	0	0	0	8	0	0	0	1	2	1	29	2653	5132	7826		3	0	0	0	0	0	0	0	0	0
DDB_G0287561	DDB_G0287561	terminal differentiation	too little data	rblA-activated during develop.	2.149573647815313	0.09597308189382026			NADH:flavin oxidoreductase/NADH oxidase domain-containing protein	0	1	0	2	46	27	1	6	74	9	0.00161366779594262	0.999998877031569			0	0	0	0	3	1	1	0	0	0	1	5	685	1302	1998		0	0	0	0	0	0	0	0	0	0
DDB_G0288489	DDB_G0288489	terminal differentiation	too little data	rblA-activated during develop.	0.6141638993758037	0.04976493014230602				0	7	0	4	58	54	0	7	119	11	0.000140812124917535	0.999998877031569			2	0	0	0	1	2	0	6	2	2	3	14	1445	2462	3939		0	0	0	0	0	0	0	0	0	0
DDB_G0267566	DDB_G0267566	max mid devel	too little data	rblA-activated during develop.	0.5373934119538283	0.09378911663457062			putative protein serine/threonine kinase, ankyrin repeat-containing protein	0	6	0	3	8	130	1	11	144	15	0.015835365068975	0.999998877031569			0	0	1	6	238	488	285	309	36	39	5	12	11	13	1443		0	1	0	0	0	0	0	0	0	0
DDB_G0268284	tgrO4	max mid devel	too little data	rblA-activated during develop.	1.0747868239076566	0.14093118361899495			immunoglobulin E-set domain-containing protein	0	0	0	1	94	306	21	25	400	47	0.0348947909409979	0.999998877031569			5	1	33	58	560	1027	445	359	57	113	16	49	28	39	2790		0	0	0	0	0	0	0	0	0	0
DDB_G0268304	tgrO3	max mid devel	too little data	rblA-activated during develop.	0.5373934119538283	0.14387465696108226			immunoglobulin E-set domain-containing protein	0	4	0	2	25	218	5	26	247	33	0.0418191580723006	0.999998877031569			0	0	19	84	188	241	115	76	23	33	2	6	7	8	802		0	0	0	0	0	0	0	0	0	0
DDB_G0270086	DDB_G0270086	max mid devel	too little data	rblA-activated during develop.	1.1239288730484365	0.04685225893564385			lipocalin family protein	1	92	2	23	87	853	4	54	1033	83	0.000236317476392619	0.999998877031569			35	23	60	536	154	374	266	465	161	404	45	223	36	184	2966		0	0	1	0	1	4	0	0	0	0
DDB_G0270342	DDB_G0270342	max mid devel	too little data	rblA-activated during develop.	12.608533421155869	0.11798686502660866				0	3	1	34	530	3039	83	326	3572	444	0.000755639868959447	0.00993043925684411			1	2	12	18	935	1825	5014	4492	2664	6167	212	898	152	498	22890		2	0	0	1	0	0	0	0	0	0
DDB_G0270600	DDB_G0270600	max mid devel	too little data	rblA-activated during develop.	1.0747868239076566	0.02357286164635548			unknown	0	3	0	3	10	114	0	7	127	10	0.00249005759134834	0.999998877031569			0	0	0	0	1	7	23	27	19	47	12	29	13	26	204		0	0	0	0	0	0	0	0	0	0
DDB_G0271770	DDB_G0271770	max mid devel	too little data	rblA-activated during develop.	1.2686117147283134	0.07797872338840114			unknown	2	13	4	5	189	179	16	17	383	42	0.0014640498531049	0.999998877031569			67	113	1984	2980	40	55	23	9	5	23	2	14	9	25	5349		0	4	3	6	2	3	0	0	0	0
DDB_G0271908	mrrA	max mid devel	too little data	rblA-activated during develop.	1.6741173662392606	0.14215585549800336			transmembrane protein	2	21	4	16	188	756	36	95	967	151	0.0346821391959648	0.999998877031569			21	15	1808	5648	715	789	272	221	51	110	12	78	21	65	9826		1	4	2	2	0	5	4	1	1	3
DDB_G0272042	DDB_G0272042	max mid devel	too little data	rblA-activated during develop.	2.1780467195732536	0.10049181531755963				1	13	2	16	309	1628	44	130	1951	192	0.00376630496832099	0.999998877031569			0	1	1	3	62	134	1012	985	612	1603	159	573	128	406	5679		0	0	0	0	0	0	0	0	0	0
DDB_G0272316	DDB_G0272316	max mid devel	too little data	rblA-activated during develop.	3.2243604717229695	0.017718502600117748			unknown	0	1	0	3	4	65	0	3	70	6	0.00557536404121249	0.999998877031569			0	0	0	0	94	177	130	196	25	35	3	13	8	9	690		0	0	0	0	0	0	0	0	0	0
DDB_G0273367	DDB_G0273367	max mid devel	too little data	rblA-activated during develop.	0.5373934119538283	0.13453612208068247				0	4	0	2	51	144	9	18	199	29	0.0466807295803766	0.999998877031569			0	1	0	2	28	84	303	333	142	488	45	215	48	225	1914		0	0	0	0	0	0	0	0	0	0
DDB_G0275439	cad2	max mid devel	too little data	rblA-activated during develop.	1.1187438860838812	0.14726025952308602			putative adhesion molecule	12	192	16	98	307	1373	57	201	1884	372	0.041479643220909	0.999998877031569			520	174	3605	12076	1146	1973	310	284	24	63	20	103	41	123	20462		10	10	12	4	3	14	7	5	0	6
DDB_G0279337	DDB_G0279337	max mid devel	too little data	rblA-activated during develop.	2.197064252649571	0.06820920446799329				3	16	14	3	1014	1139	102	56	2172	175	0.00106973874695542	0.999998877031569			26	11	7934	5237	1644	2585	298	164	14	72	12	71	44	158	18270		0	5	1	0	0	0	0	0	0	0
DDB_G0280547	comH	max mid devel	max early G2	rblA-activated during develop.	0.014365025215599599	0.02860909109380999			putative GATA-binding transcription factor, GATA zinc finger domain-containing protein 2	52	175	1	1	120	151	7	0	498	9	0.0000000015128596302599	0.0000318473672307214			16	16	578	817	618	1771	437	267	22	104	6	41	19	61	4773		74	59	34	62	24	87	121	138	53	163
DDB_G0280643	DDB_G0280643	max mid devel	too little data	rblA-activated during develop.	0.4960554571881492	0.0014486826025253503			putative protein serine/threonine kinase, protein kinase, CMGC group	0	13	0	6	52	795	0	3	860	9	0.00000000725961726579851	0.999998877031569			0	0	109	566	345	637	655	795	393	693	105	290	119	197	4904		1	0	1	1	0	2	0	0	0	1
DDB_G0280717	DDB_G0280717	max mid devel	too little data	rblA-activated during develop.	1.6218236656553795	0.13232151267430337			putative protein serine/threonine kinase, protein kinase, AGC group, MAST family protein kinase	0	9	1	10	5	157	1	14	171	26	0.0196650279160875	0.999998877031569			0	0	4	25	129	196	163	242	123	219	24	43	18	15	1201		0	0	1	1	1	1	0	0	0	1
DDB_G0280847	2C	max mid devel	too little data	rblA-activated during develop.	4.726763379166387	0.10608971210628314			coiled-coil family protein, hssA/2C/7E family protein	0	0	1	4	1012	905	120	113	1917	238	0.0062829659797107	0.461402286898053			0	1	0	0	23	54	538	504	482	1371	77	322	113	516	4001		0	0	0	0	0	0	0	0	0	0
DDB_G0280859	DDB_G0280859	max mid devel	too little data	rblA-activated during develop.	1.0747868239076566	0.03317662009487068				0	1	0	1	1	81	0	7	83	8	0.0350829147072795	0.999998877031569			0	0	0	0	24	20	53	69	41	39	4	7	6	5	268		0	0	0	0	0	0	0	0	0	0
DDB_G0280871	DDB_G0280871	max mid devel	too little data	rblA-activated during develop.	4.299147295630626	0.07469031615651722			coiled-coil family protein, hssA/2C/7E family protein	0	0	0	4	489	523	43	43	1012	90	0.00108230074171911	0.642173226353793			0	0	0	0	21	14	679	442	445	1196	98	415	141	515	3966		0	0	0	0	0	0	0	0	0	0
DDB_G0280873	DDB_G0280873	max mid devel	too little data	rblA-activated during develop.	3.2243604717229695	0.07816896410807861			coiled-coil family protein, hssA/2C/7E family protein	0	1	0	3	389	442	31	45	832	79	0.00106973874695542	0.999998877031569			0	0	0	0	8	7	105	109	314	701	59	210	90	279	1882		0	0	0	0	0	0	0	0	0	0
DDB_G0280949	DDB_G0280949	max mid devel	too little data	rblA-activated during develop.	1.0747868239076566	0.14030442158038267			coiled-coil family protein, hssA/2C/7E family protein	0	0	0	1	438	393	59	76	831	136	0.0212916650087802	0.999998877031569			0	0	1	0	12	26	207	127	104	218	14	43	20	92	864		0	0	0	0	0	0	0	0	0	0
DDB_G0280953	DDB_G0280953	max mid devel	too little data	rblA-activated during develop.	8.598294591261253	0.14241935396756117			coiled-coil family protein, hssA/2C/7E family protein	0	0	0	8	753	985	93	210	1738	311	0.0219023085646882	0.223206764663401			0	0	2	10	8	16	101	73	168	565	25	125	32	191	1316		0	0	0	0	0	0	0	0	0	0
DDB_G0281689	DDB_G0281689	max mid devel	too little data	rblA-activated during develop.	0.5373934119538283	0.06276328851350396			unknown	3	24	0	12	13	92	1	6	132	19	0.0062829659797107	0.999998877031569			2	2	1	8	158	394	187	310	13	49	4	21	7	7	1163		1	0	0	0	0	1	0	0	0	0
DDB_G0282551	DDB_G0282551	max mid devel	too little data	rblA-activated during develop.	1.0747868239076566	0.18331527597115235			weakly related to hssA/2C/7E family protein	1	5	0	5	22	352	7	25	380	37	0.0118596729088979	0.999998877031569			0	0	0	0	2	2	20	41	19	23	10	9	10	12	148		0	0	0	0	0	0	0	0	0	0
DDB_G0283613	cbpC	max mid devel	too little data	rblA-activated during develop.	0.42991472956306265	0.13326023334525486			calcium-binding protein	0	30	0	12	80	725	15	78	835	105	0.0158357328571419	0.999998877031569			4	11	1307	5238	805	782	169	101	60	92	4	31	13	25	8642		0	2	0	0	1	0	0	0	0	0
DDB_G0284527	DDB_G0284527	max mid devel	too little data	rblA-activated during develop.	1.0747868239076566	0.08402516255782898				0	1	0	1	23	134	3	7	158	11	0.00408929547232	0.999998877031569			4	0	88	421	91	94	14	15	0	0	2	4	1	1	735		0	0	0	0	0	0	0	1	0	0
DDB_G0284529	DDB_G0284529	max mid devel	too little data	rblA-activated during develop.	1.0417799829115648	0.03643775441157063				0	7	1	4	23	127	1	5	157	11	0.000424545961680965	0.999998877031569			3	2	800	3715	12	13	2	3	1	4	0	5	2	5	4567		0	0	0	0	0	0	0	0	0	0
DDB_G0284983	colD	max mid devel	too little data	rblA-activated during develop.	2.6869670597691413	0.13276113524079772			colossin D, Cna B-type domain-containing protein	0	4	0	10	194	1112	42	77	1310	129	0.00998824008177635	0.999998877031569			0	0	0	2	844	1292	1409	1456	303	605	10	43	6	42	6012		0	0	0	0	0	0	0	0	0	0
DDB_G0285697	DDB_G0285697	max mid devel	too little data	rblA-activated during develop.	1.4572690105918684	0.032778294664016897				5	56	13	18	32	903	2	5	996	38	0.000000125908858563046	0.999998877031569			1	0	1	7	327	1323	634	372	239	786	27	195	9	21	3942		3	2	1	2	0	6	1	0	1	1
DDB_G0286685	sigN175	max mid devel	too little data	rblA-activated during develop.	0.6514819880832691	0.16783990134673935			related to the hssA genes	3	88	2	30	161	1475	41	165	1727	238	0.0299632320809843	0.999998877031569			0	0	0	1	22	11	159	186	38	67	10	27	13	35	569		0	0	0	0	0	0	0	0	0	0
DDB_G0286689	DDB_G0286689	max mid devel	too little data	rblA-activated during develop.	0.6035327442557389	0.04177843239470953			unknown	3	27	2	8	63	866	4	24	959	38	0.0000163040123578152	0.999998877031569			0	0	0	0	3	4	80	121	15	50	9	20	7	17	326		0	0	0	1	0	0	0	0	0	0
DDB_G0286781	sigN173	max mid devel	too little data	rblA-activated during develop.	0.30708194968790187	0.2227529310241657			hssA/2C/7E family protein	0	7	0	2	22	243	9	14	272	25	0.0296374169202984	0.999998877031569			0	0	0	0	171	685	228	520	55	140	27	108	24	93	2051		0	0	0	0	0	0	0	0	0	0
DDB_G0287863	DDB_G0287863	max mid devel	too little data	rblA-activated during develop.	2.2135639493181523	0.0915642373389047			unknown	3	30	10	22	348	837	50	46	1218	128	0.00392132833661926	0.999998877031569			9	4	25	59	1781	3942	2004	2502	372	1450	9	129	8	76	12370		3	0	0	3	1	11	1	4	0	3
DDB_G0288869	DDB_G0288869	max mid devel	too little data	rblA-activated during develop.	1.2743560873128916	0.1136002618277275			EGF-like domain-containing protein	7	48	4	46	45	170	4	31	270	85	0.0386146338501033	0.999998877031569			4	5	428	942	24	4	8	8	10	10	3	7	2	12	1467		1	4	2	5	5	12	2	0	0	2
DDB_G0288967	DDB_G0288967	max mid devel	too little data	rblA-activated during develop.	6.78399675780881	0.12557367557574925				1	12	10	28	706	1669	96	256	2388	390	0.0188580639664505	0.524235920804028			7	1	2	2	30	57	386	460	482	810	181	408	239	732	3797		0	1	0	0	0	1	0	0	0	2
DDB_G0290847	DDB_G0290847	max mid devel	too little data	rblA-activated during develop.	1.0747868239076566	0.048103733168999255				0	1	0	1	19	155	1	9	175	11	0.00149256788348436	0.999998877031569			0	0	4	2	125	166	55	62	31	78	4	13	3	9	552		0	0	0	0	0	0	0	0	0	0
DDB_G0290855	DDB_G0290855	max mid devel	too little data	rblA-activated during develop.	0.716524549271771	0.10345165913888998				1	6	0	4	23	148	4	7	178	15	0.00538224989189485	0.999998877031569			0	0	0	0	84	188	51	38	30	59	3	6	2	8	469		1	0	0	1	0	1	1	0	0	0
DDB_G0291514	tgrA4	max mid devel	too little data	rblA-activated during develop.	0.3838524371098774	0.07501594126063633			transmembrane protein, immunoglobulin E-set domain-containing protein	0	14	0	5	8	84	1	3	106	9	0.00381696922860874	0.999998877031569			0	0	13	25	120	221	53	85	8	19	0	5	2	4	555		0	0	0	0	0	0	0	0	0	0
DDB_G0291516	DDB_G0291516	max mid devel	too little data	rblA-activated during develop.	1.4642780339525276	0.048566293671488			putative protein kinase, ankyrin repeat-containing protein	2	30	4	17	58	651	4	25	741	50	0.0000744287663486653	0.999998877031569			2	2	82	182	719	1432	1085	1091	203	546	26	111	29	95	5605		1	7	3	5	2	2	2	1	0	1
DDB_G0291604	DDB_G0291604	max mid devel	too little data	rblA-activated during develop.	0.15354097484395093	0.08035134900053398			beta-ketoacyl synthase family protein	0	7	0	1	23	43	0	9	73	10	0.0209332644443876	0.999998877031569			0	0	2	3	36	30	29	34	41	35	11	29	26	17	293		0	0	0	0	0	0	0	0	0	0
DDB_G0291664	DDB_G0291664	max mid devel	cold-activated?	rblA-activated during develop.	1.1080243688665754	0.08105086550133167			putative protein kinase, ankyrin repeat-containing protein	60	479	50	335	281	3096	35	161	3916	581	0.000322866520651945	0.999998877031569			2	8	231	623	2314	4964	4150	4177	504	1594	93	350	88	348	19446		86	172	117	63	31	145	46	48	16	39
DDB_G0291668	tgrA3	max mid devel	too little data	rblA-activated during develop.	0.5373934119538283	0.17903804749461777			immunoglobulin E-set domain-containing protein	0	8	0	4	18	287	5	32	313	41	0.0384686071015585	0.999998877031569			0	0	13	33	100	111	116	87	46	60	4	18	7	21	616		0	0	0	0	0	0	0	0	0	0
DDB_G0293112	DDB_G0293112	max mid devel	too little data	rblA-activated during develop.	0.5373934119538283	0.17317723043099956			unknown	0	2	0	1	14	151	4	13	167	18	0.0384686071015585	0.999998877031569			0	0	0	0	6	17	174	262	23	50	4	21	8	26	591		0	0	0	0	0	0	0	0	0	0
DDB_G0293638	DDB_G0293638	max mid devel	too little data	rblA-activated during develop.	1.6121802358614847	0.1537734524301793			unknown	0	2	0	3	188	1148	36	179	1338	218	0.0526102984658588	0.999998877031569			0	0	0	1	80	139	343	450	473	739	59	167	52	124	2627		0	0	0	0	0	0	0	0	0	0
DDB_G0293822	DDB_G0293822	max mid devel	too little data	rblA-activated during develop.	0	0.007527468424886625				2	3	0	0	3	51	0	1	59	1	0.00235991876763446	0.708740968781197			0	0	2	0	17	32	36	29	7	14	0	6	1	5	149		0	0	0	0	0	0	0	0	0	0
									total mappable reads 	3208678	21432352	3826379	9970513	5720809	10815100	5811380	14085797	41176939	33694069					3248410	2820853	4712062	5373309	2600425	4624360	5129256	3776273	2429810	5153928	1776805	3816780	2651815	5937874			4849631	3342896	2060722	2755790	1312264	5289980	2110911	1944835	768136	2503229
this gene:
DDB_G0276451 (SIVA)
confidence level      (0 to 1) 	0.95
Click  on Gene Name,
(column B) then press:
cntrl g	graph this line
cntrl n	graph next line
cntrl p	graph previous line
macros must be activated!
sorry, Windows only!
[MacIntosh users can copy line
 of data to line 10 on next page]
	insert rad1 and rad17 for comparison with rad9; line 76
	insert H4a
	ADD collectiive IDs for centrosomal proteins
	check for collectivfes for arrH,J,and K
... (truncated at 50 columns)

## Sheet: graph_plotter

																										0	0
Dictybase ID	Gene name								Function	mappable reads wt growth phase biological 1	mappable reads wt growth phase biological 2	mappable reads rblA disruptant growth phase biological 1	mappable reads rblA disruptant growth phase biological 2	mappable reads wt late devel. biological 1	mappable reads wt late devel. biological 2	mappable reads rblA disruptant late devel. biological 1	mappable reads rblA disruptant late devel. biological 2			adjusted p-value (development)	adjusted p-value (growth)			mappable reads 0h biological 1	mappable reads 0h biological 2	mappable reads 4h biological 1	mappable reads 4h biological 2	mappable reads 8h biological 1	mappable reads 8h biological 2	mappable reads 12h biological 1	mappable reads 12h biological 2	mappable reads 16h biological 1	mappable reads 16h biological 2	mappable reads 20h biological 1	mappable reads 20h biological 2	mappable reads 24h biological 1	mappable reads 24h biological 2	total mappable reads		mappable reads asynchronous	mappable reads T0	mappable reads T0 replicate	mappable reads T1	mappable reads T1 replicate	mappable reads T1 replicate	hits T2	mappable reads T3	mappable reads T3 replicate	mappable reads T4
DDB_G0276451	DDB_G0276451 (SIVA)	max mid devel	max late G2	growth&devel	5.43	10.57		Conserved protein, cell-cycle role not understood	possible orthologue of SIVA, an E2F-induced proapoptotic protein in vertebrates, see Fortin et al, JBC 279, 28706-28714 (2004)	3	91	26	235	5	28	57	378	127	696	0.000394249558473038	0.393101819001763			0	0	1	1	4	7	16	20	10	18	1	3	1	3	85		14	7	4	20	14	44	8	9	1	7
TOTALS										3208678	21432352	3826379	9970513	5720809	10815100	5811380	14085797	41176939	33694069					3248410	3248410	2820853	4712062	5373309	2600425	4624360	5129256	3776273	2429810	5153928	1776805	3816780	2651815	5937874		4849631	3342896	2060722	2755790	1312264	2110911	2110911	1944835	768136	2503229
												mappable reads wt growth phase biological1	mappable reads rblA disruptant growth phase biological1	mappable reads wt late devel. biological1	mappable reads rblA disruptant late devel. biological1	total reads wildtype	total hits rblA disruptant	Sum of all reads														mappable reads 0h	mappable reads 4h	mappable reads 8h	mappable reads 12h	mappable reads 16h	mappable reads 20h	mappable reads 24h	total
confidence level:	0.95
upper p  boundary	0.975										total mappable reads for the experiment	3208678	3826379	5720809	5811380	8929487	9637759													total mappable reads for the experiment		6496820	7532915	7973734	9753616	6206083	6930733	6468595
lower p boundary	0.025000000000000022										total mappable reads for a particular gene	3	26	5	57	8	83	91												total mappable reads for a particular gene		0	2	11	36	28	4	4	85
Z value	1.9599639845400536										lower confidence limit	0.6186721228956023	16.984063215596343	1.6234863901184198	43.17124336292474	3.453832176748501	66.10903669780309													lower confidence limit		0	0.24220927854396515	5.491160367236838	25.213957417315232	18.60579665585753	1.0898653736263253	1.0898653736263253
											upper confidence limit	8.767273069742325	38.09602408312502	11.668332079322669	73.85011140296281	15.763189220193315	102.89098779726608													upper confidence limit		3.6888794541139354	7.224687667723959	19.682038513301954	49.839174489236186	40.46779594326819	10.241588675403698	10.241588675403698
											mappable reads per million per kilobase-pairs of exon (RPKM)	1.7410884416482268	12.653511901464778	1.6275647315997177	18.265068089962806	1.66835778397899	16.037170426513352	34.28723316467553	17.705528210492343											mappable reads per million per kilobase-pairs of exon (RPKM)		0	0.49441614379656473	2.568955939859562	6.873256661452675	8.401680577243681	1.0747477318336458	1.1515312941519138	20.56458834833804
											lower confidence limt	0.35905429411450146	8.26569407822225	0.528465838157776	13.83378420266974	0.7202784745794324	12.773516725967395													lower confidence limt		0	0.05987608874472764	1.2824135492849538	4.813944466115167	5.582855728130844	0.2928325845772305	0.3137535210358204
											upper confidence limit	5.088199268834043	18.540326697473514	3.7981931537798346	23.664514267130777	3.287329929480402	19.88048562237424													upper confidence limit		1.0573513968773318	1.7860011084053384	4.596571795208339	9.515484390540133	12.142767685014894	2.7517810498158193	2.948377465389801
											normalized profile (to give a total of 1)	0.050779496650723795	0.36904441489029444	0.047468535118678476	0.5327075533403034	0.09422807182845394	0.9057719281715461	1	1											normalized profile (to give a total of 1)		0	0.024042112364312012	0.12492134033245436	0.334227777625714	0.40855087565721565	0.05226205910999931	0.055995834910304756	1
											lower confidence limit	0.010471952997491138	0.24107206430228945	0.01541290414480655	0.4034674987109201	0.040680993304260386	0.7214422848112363													lower confidence limit		0	0.0029116113452164784	0.062360283005061655	0.23408902646496269	0.27147908985895314	0.014239652144600122	0.015256980384009333
											upper confidence limit	0.1483992378269871	0.5407355737462862	0.11077572621674614	0.6901844238487922	0.18566686576073577	1.1228405832362014													upper confidence limit		0.05141612265546679	0.08684837635223935	0.2235187846869698	0.4627121257843773	0.5904697667335621	0.1338116281835619	0.14337157717178806
											error up	0.09761974117626332	0.1716911588559918	0.06330719109806766	0.1574768705084888	0.09143879393228183	0.21706865506465534													error up		0.05141612265546679	0.06280626398792734	0.09859744435451545	0.12848434815866333	0.18191889107634646	0.08154956907356259	0.0873757422614833
											error down	0.04030754365323266	0.127972350588005	0.03205563097387193	0.12924005462938332	0.053547078524193555	0.18432964336030977													error down		0	0.021130501019095534	0.0625610573273927	0.1001387511607513	0.1370717857982625	0.038022406965399186	0.04073885452629542
											x axis	wt growth	rblA- growth	wt     devel	rblA- devel	wt     total	rblA- total													x axis		0	4	8	12	16	20	24
												mappable reads wt growth phase biological2	mappable reads rblA disruptant growth phase biological2	mappable reads wt late devel. biological2	mappable reads rblA disruptant late devel. biological2	total reads wildtype	total hits rblA disruptant	Sum of all reads
											total mappable reads for the experiment	21432352	9970513	10815100	14085797	32247452	24056310
											total mappable reads for a particular gene	91	235	28	378	119	613	732
											lower confidence limit	73.26752349683106	205.91239175125284	18.60579665585753	340.8497575976469	98.58163317723229	564.4735715605867
											upper confidence limit	111.72783491654769	267.04521775001626	40.46779594326819	418.0945713023997	142.40124187018748	662.5176736125597
											mappable reads per million per kilobase-pairs of exon (RPKM)	7.906736636093514	43.89106030322188	4.82117844512415	49.97307674710487	6.871906957421452	47.45229014184625	106.5920521315444	54.3241970992677
											lower confidence limt	6.366011123826777	38.45835405759526	3.2036380639851187	45.061669565236244	5.692805133652034	43.6958624716104
											upper confidence limit	9.707720501165669	49.87616066362971	6.967945197263112	55.27373571228236	8.223261216413414	51.28545003648316
											normalized profile (to give a total of 1)	0.07417754399113992	0.4117667258066884	0.04523018694840748	0.4688255432537643	0.12649808601615	0.87350191398385	1	1
											lower confidence limit	0.05972313128909961	0.3607994525720746	0.030055130752446105	0.4227488697715097	0.10479317574173175	0.8043535809975078
											upper confidence limit	0.09107358669842897	0.46791631895854613	0.0653702134251438	0.5185540066727441	0.15137381968824837	0.9440627340109276
											error up	0.016896042707289047	0.056149593151857746	0.020140026476736322	0.049728463418979796	0.02487573367209836	0.07056082002707753
											error down	0.014454412702040308	0.05096727323461381	0.015175056195961378	0.04607667348225458	0.02170491027441826	0.06914833298634226
											x axis	wt growth	rblA- growth	wt     devel	rblA- devel	wt     total	rblA- total
											Average	0.06247852032093186	0.39040557034849144	0.04634936103354298	0.5007665482970338	0.11036307892230197	0.889636921077698
											error up	0.05725789194177618	0.11392037600392477	0.04172360878740199	0.1036026669637343	0.05815726380219009	0.14381473754586643
											error down	0.027380978177636482	0.0894698119113094	0.023615343584916654	0.08765836405581895	0.037625994399305904	0.12673898817332602
... (truncated at 50 columns)
